# Discovery of hybrid chemical synthesis pathways with DORAnet

**DOI:** 10.1039/d5dd00229j

**Published:** 2025-09-26

**Authors:** Quan Zhang, William W. Sprague, Shivani S. Kozarekar, Stefan C. Pate, Taylor Uekert, Linda J. Broadbelt

**Affiliations:** a Department of Chemical and Biological Engineering, Northwestern University 2145 Sheridan Road Evanston Illinois 60208 USA broadbelt@northwestern.edu; b Strategic Energy Analysis Center, National Renewable Energy Laboratory 15013 Denver West Parkway Golden Colorado 80401 USA

## Abstract

Developing efficient tools for discovering novel synthesis pathways is essential to advance chemical production methods that maximize the use of resources and energy. We introduce DORAnet (Designing Optimal Reaction Avenues Network Enumeration Tool), an open-source computational framework that addresses key limitations in current computer-aided synthesis planning (CASP) tools. DORAnet integrates both chemical/chemocatalytic (*i.e.*, non-enzymatic) and enzymatic transformations, enabling the discovery of hybrid synthesis pathways. With 390 expert-curated chemical/chemocatalytic reaction rules and 3606 enzymatic rules derived from MetaCyc, it provides extensive flexibility for synthetic chemists and biotechnologists. The framework features customizable network expansion strategies, advanced filtering, and pathway search, ranking, and visualization tools. Validated against known reaction data, DORAnet successfully identified both established and novel synthesis routes for key industrial chemicals. In a case study involving 51 high-volume targets, DORAnet frequently ranked known commercial pathways among the top three results, demonstrating its practical relevance and ranking accuracy, while also uncovering numerous alternative (hybrid) synthesis pathways that were highly ranked.

## Abstract 2

DORAnet is a flexible tool that integrates chemocatalytic and enzymatic transformations to explore chemical space, uncover novel routes, and rank diverse synthesis pathways.

## Graphical abstract

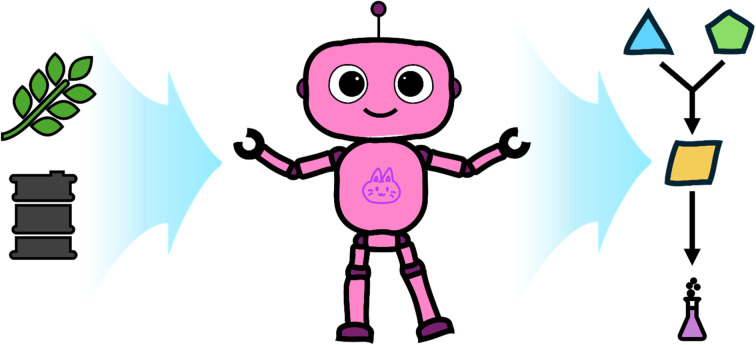

## 1. Introduction

The discovery of novel synthesis pathways remains a fundamental challenge in chemistry, particularly in the pursuit of production methods from unconventional (non-fossil) resources.^1^ Historically reliant on expert insight, the field has seen rapid advancements with the emergence of computer-aided synthesis planning (CASP) tools, which provide powerful platforms for designing novel synthetic routes.^2–11^ These tools typically employ a reaction network generator to predict possible reactions from a set of starter molecules. By recursively applying this process to both the starter molecules and their products, a reaction network is constructed, enabling pathway discovery.^12^

Comprehensive reviews of reaction network generators and their areas of application can be found in the literature.^9,12–14^ Based on their approach to reaction prediction, these generators can be classified into two basic categories:^9,15^ template-based and template-free. Template-based methods^16^ rely on predefined reaction rules (templates) that are either manually curated or extracted through data-mining of reaction databases. Template-free methods^17^ leverage generative models, such as neural networks, to directly predict products. Each approach presents distinct advantages and limitations depending on the application and constraints. Our research focuses on the template-based approach. While more restricted than template-free methods, the use of templates produces accurate, explainable predictions, enables direct control over outputs (*e.g.*, by adding custom rules), and is not susceptible to hallucinations or training data biases inherent in generative models.^9,18^

Despite the capabilities of existing CASP frameworks, they often allow only limited customization^18^ and provide constrained analytical capabilities.^19^ Additionally, while current tools specialize in either chemocatalytic^16^ or enzymatic^10,19^ reaction pathway discovery, few can predict both types of reactions and explore hybrid pathways.^8,20^

In this work, we introduce DORAnet (Designing Optimal Reaction Avenues Network Enumeration Tool),^21,22^ an open-source template library-based computational framework. DORAnet (formerly known as Pickaxe-Generic) was designed to overcome the software and distribution limitations of NetGen^16^ and Pickaxe,^19^ two network expansion tools previously developed by our group. Unlike many computational frameworks, which focus exclusively on either chemical/chemocatalytic or enzymatic transformations, DORAnet integrates both, enabling hybrid pathway discovery. As an open-source platform, it provides full control over reaction rules, expansion strategies, analysis methods, data output, and custom functionalities, offering a comprehensive solution for researchers and industrial chemists.

A key motivation behind DORAnet is the increasing demand for chemical synthesis routes that utilize alternative feedstocks. Traditional petrochemical-based processes, while efficient from an atom-economy perspective, often exhibit high energy consumption.^23^ Enzymatic pathways, by contrast, offer highly selective and benign transformations but are often limited in scope.^24,25^ By enabling hybrid pathway exploration that leverages both chemical/chemocatalytic and enzymatic transformations, DORAnet unlocks new opportunities in biomass utilization and energy efficient manufacturing. This paper presents an overview of DORAnet, including its architecture, reaction rule curation, network expansion strategies, and ranking methodologies. Its performance is evaluated through validation against known reaction datasets and a case study on alternative synthesis routes for 51 key industrial chemicals.

## 2. DORAnet

### 2.1 Development

Two computational tools for exploring complex chemical pathways and discovering new reactions have been previously developed and utilized by Broadbelt and colleagues,^14,16,18–20,26–29^ culminating in the recent release of DORAnet. The evolution began with NetGen,^16^ an early tool for automated network generation. Written in C and C++, NetGen employs graph-based algorithms to represent molecules and their transformations, enabling systematic exploration of reaction pathways from a set of initial reactants and reaction rules. It also incorporates a Benson group additivity scheme to estimate reaction thermodynamics. Despite its continued use in mechanistic reaction kinetics studies,^13^ NetGen has several drawbacks. Its custom C algorithms limit user accessibility compared to modern Python tools and design practices. The customization of reaction rules requires tailored user expertise, hindering the development of comprehensive rule sets. Finally, its closed-source licensing restricts community contributions and enhancements.

Building on the concepts of NetGen, Pickaxe^19^ was released as an open-source tool in 2023, specifically designed for metabolic pathway exploration. Written in Python, Pickaxe enhances accessibility and customization. It employs SMILES arbitrary target specification (SMARTs) for reaction rule formulation, simplifying rule creation through Python-based scripting. Prepackaged with enzymatic reaction rules derived from reactions in MetaCyc,^14,30^ Pickaxe also allows users to incorporate custom rules. However, its specialization in biochemical transformations limits its applicability in chemical/chemocatalytic chemistry more broadly. Additionally, it has limited support for bimolecular reactions and offers only a small menu of network expansion strategies.

DORAnet represents the latest advancement, incorporating significant improvements while maintaining the strengths of its predecessors. Like Pickaxe, it is open-source and written in Python, but with a more streamlined design and an emphasis on transferability, customization, and user experience. DORAnet includes approximately 390 expert-curated chemical/chemocatalytic reaction rules for transformations of molecules and intermediates containing C, H, O, N, and S atoms from organic chemistry literature, along with 3606 enzymatic reaction rules^14,30^ derived from MetaCyc reactions. It supports reaction network generation using either chemical/chemocatalytic or enzymatic rules, or both, facilitating hybrid pathway discovery. The interface accommodates both novice and advanced users, offering a simplified workflow for routine tasks while allowing in-depth customization of reaction rules, filters, and expansion strategies. This flexibility is particularly valuable when computational resources are constrained, as custom filters and expansion strategies can reduce memory usage during network expansions. Comprehensive GitHub tutorials and interactive exercises on Colab further enhance accessibility (see Data Availability).

### 2.2 Architecture and workflow

**Fig. 1:**
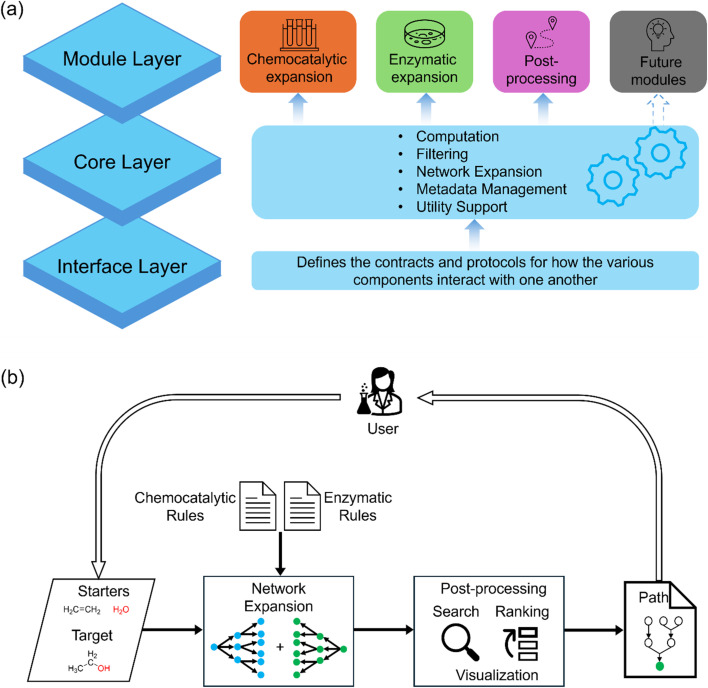
The architecture and workflow of DORAnet. (a) DORAnet's layered architecture, illustrating its modular design, which comprises three primary layers. The module layer supports a range of functionalities by invoking core routines through accessible functions and methods, thereby simplifying user operations and facilitating the integration of new features without overhauling the core routines. The core layer houses the primary logic and implements the underlying computational processes. The interface layer defines standardized contracts for component communication, ensuring seamless and reliable interactions between layers. (b) Overview of the DORAnet workflow. Users start by specifying starter and target molecules and setting run parameters. DORAnet then automatically expands the reaction network using pre-packaged or custom-defined reaction rules, identifying and ranking candidate pathways based on customizable criteria. The final output is a comprehensive PDF file with all identified pathways, visually mapped and ranked for easy interpretation and selection.

DORAnet is structured as a modular, object-oriented computational platform. It leverages RDKit for molecule manipulation and SMARTS for reaction rule representation. The core design philosophy prioritizes customizability, scalability, and seamless integration of chemical/chemocatalytic and enzymatic pathways within a unified framework. The architecture balances computational efficiency with flexibility, making it suitable for exploring complex chemical spaces. From a programmer's perspective, DORAnet's architecture consists of three primary layers, as shown in Fig. 1(a). From a user's perspective, the DORAnet workflow consists of three primary steps: curation of reaction rules (if not using prepackaged rules), network expansion, and post-processing. The workflow is illustrated in Fig. 1(b).

## 3. Method

### 3.1 Reaction rules

**Fig. 2:**
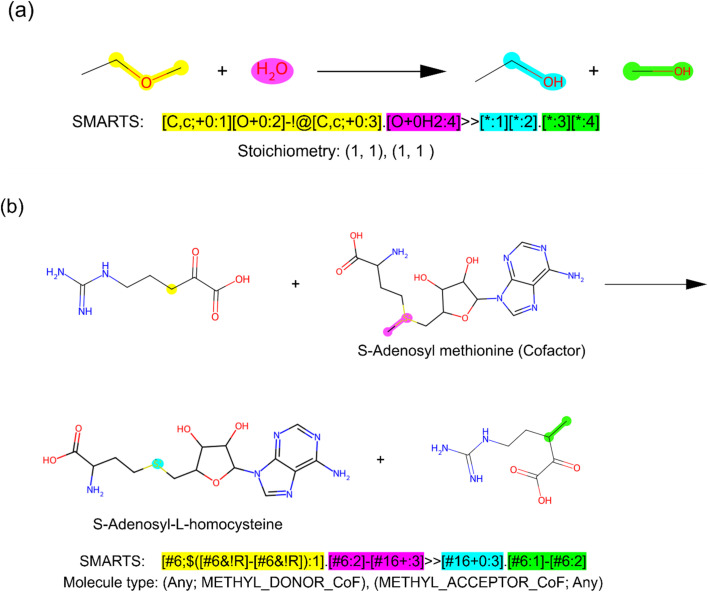
Examples of chemical/chemocatalytic and enzymatic reaction rules. (a) Chemical/chemocatalytic reaction rule for the hydrolysis of ethers. This rule identifies an ether functional group, where two carbon atoms (mapped as 1 and 3) are connected by an oxygen atom (mapped as 2). The transformation breaks one of the C–O bonds and adds the oxygen atom from water (mapped as 4) to the remaining carbon atom (mapped as 3), forming two alcohols. Stoichiometric information is included with each chemical/chemocatalytic rule to support atom economy calculations and ensure mass balance. (b) Enzymatic reaction rule for rule0043_12. This rule transfers the sulfur-bound methyl group (mapped as 2) from the cofactor *S*-adenosyl methionine to the carbon atom (mapped as 1) of the reactant 5-guanidino-2-oxopentanoic acid. Because enzymatic reactions often involve specific cofactors, each enzymatic rule is labeled with the molecule type of the reactants and products, and DORAnet ensures the correct cofactor is applied. This particular reaction is associated with UniProt ID D5FKJ3.

Reaction rules (operators) define how molecules transform through chemical or biological reactions, serving as templates for reactions. These rules identify specific moieties within reactants and apply a graph transformation to a reaction center. They must be general enough to predict new reactions yet specific enough to avoid generating unrealistic transformations.^4^ In DORAnet, reaction rules are encoded using SMARTS, a precise and flexible notation for defining molecular patterns. Two example reaction rules are shown in Fig. 2.

DORAnet includes approximately 390 manually curated chemical/chemocatalytic reaction rules covering C, H, O, N, and S transformations and 3605 enzymatic reaction rules (JN3604IMT), derived from MetaCyc data mining. The chemical/chemocatalytic rules were carefully compiled by the authors based on organic chemistry textbooks and peer-reviewed literature^31^ (Table S6). In this work, we use the term chemical/chemocatalytic broadly to refer to reactions from general organic chemistry, as distinct from enzymatic (biocatalytic) transformations. These reactions can, but do not necessarily, involve the use of a catalyst. Each chemical/chemocatalytic reaction rule is paired with a corresponding retrosynthetic rule, enabling both forward and reverse network expansion. To simplify reaction representations, oxygen molecules are universally used as oxidizing agents, and hydrogen molecules as reducing agents. For ionic reactions, ions are represented in their molecular forms (*e.g.*, Cl^−^ as HCl). Each rule is labeled with reaction type, stoichiometry, a customizable thermodynamic correction value, and relevant metadata for network expansion. The reaction name enhances explainability and helps users assess pathways effectively. Users can also define custom reaction rules.

The enzymatic rule set^14,30^ functions bidirectionally, as the data mining process did not distinguish reaction direction. To ensure correct reaction directionality during an enzymatic network expansion, a thermodynamic filter is recommended (discussed below). Each enzymatic rule is labeled with a reaction ID, cofactors (common co-reactants acting as currency molecules in enzymatic reactions^14^), and the UniProt ID of the MetaCyc reaction from which the rule was derived. The corresponding MetaCyc reactions provide potential enzymes for the predicted reactions.^19^ During network expansion, necessary cofactors are automatically incorporated, removing the need for their manual inclusion in starting molecules.

### 3.2 Network expansion

#### 3.2.1 Expansion strategies

**Fig. 3:**
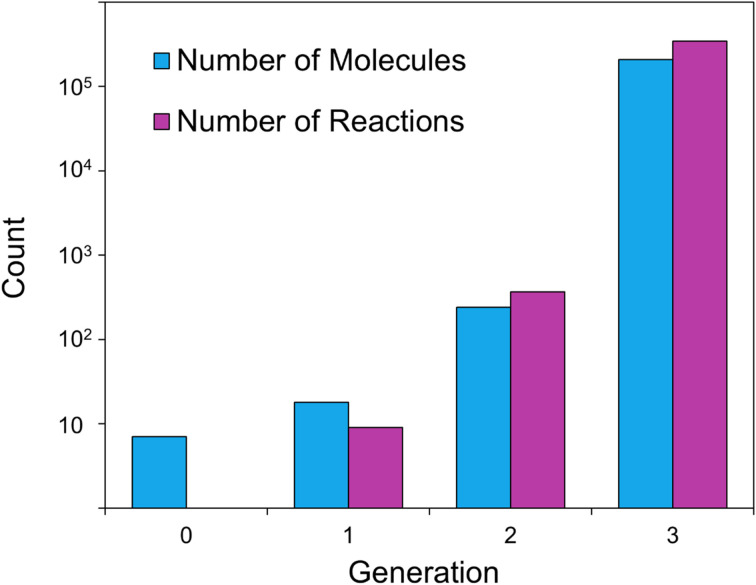
Growth of molecules and reactions during chemical/chemocatalytic network expansion. The logarithmic scale highlights the exponential trend in both counts. Starter molecule: alanine. Helper molecules: H_2_O, O_2_, H_2_, CO, HCl, Cl_2_. A reaction enthalpy threshold of 40 kcal mol^−1^ was applied.

Network expansion involves recursively applying reaction rules to reactants and their products, generating a reaction network. A key feature of DORAnet is its ability to explore chemical/chemocatalytic and enzymatic reaction spaces through various network generation strategies.^32^ These strategies are crucial in mitigating the combinatorial explosion that occurs as the number of reactions and molecules grows exponentially with each additional generation. They ensure that seeking longer, more complex pathways is feasible with limited computational resources. Fig. 3 illustrates the rapid increase in molecules and reactions during expansion. Starting with alanine and six helper molecules (which do not react with each other), only three generations of expansion can be achieved before exhausting available memory. The three-generation run produced 208 919 molecules and 342 700 reactions, requiring 2.4 GB of memory. This corresponds to an average storage requirement of approximately 12.0 KB per molecule, including its associated reactions. Alanine is simply an example here that was chosen for its simplicity and relevance in both synthetic chemistry and biology. A summary of computational performance and memory usage for typical runs for other compounds is provided in Table S1.

A fundamental strategy is forward expansion, which begins with a set of starter molecules (*e.g.*, biomass-derived feedstocks) and iteratively applies reaction rules to generate successive generations of products. This approach is well-suited for exploring chemical space and identifying novel downstream molecules, as it mirrors the natural progression of chemical synthesis, allowing systematic exploration from simple precursors. If a target molecule is not identified within computational limits, users can select promising intermediates using custom heuristics for subsequent expansion cycles. This iterative strategy extends the search space while controlling computational cost, aiding in the discovery of more complex chemicals.

The opposite approach, retro expansion, works backward from a target molecule to determine possible precursors. This method, commonly used in synthetic planning, starts with fewer molecules (the target and a few common by-products), mitigating combinatorial explosion and often allowing deeper exploration than forward expansion if the number of feedstock molecules is large. Retro expansion guarantees the inclusion of the target molecule but may miss pathways if necessary by-products are not predefined.

**Fig. 4:**
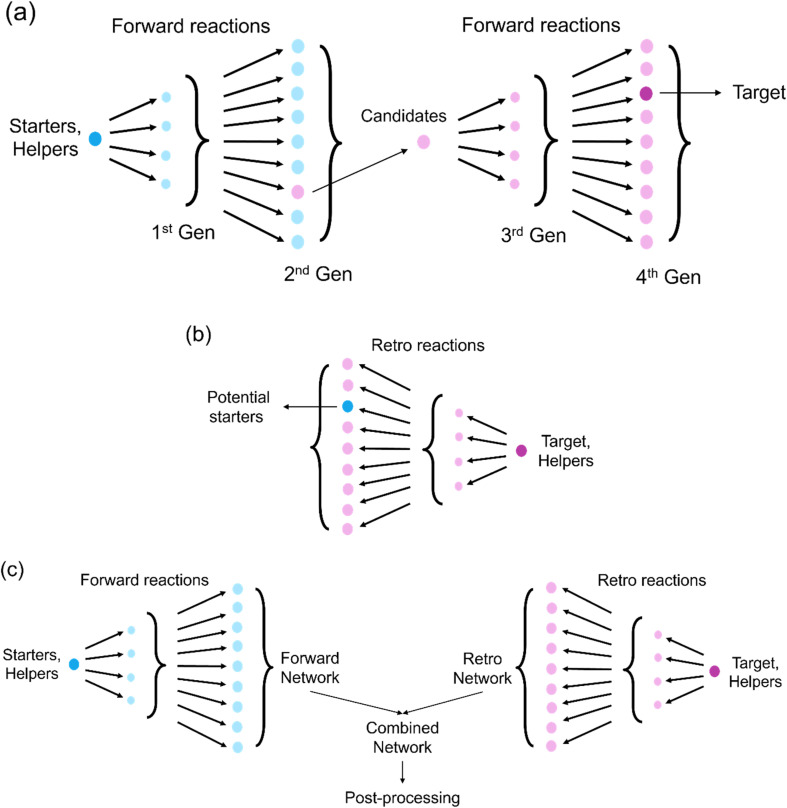
Expansion strategies in DORAnet: (a) forward expansion, (b) retro expansion, (c) combined approach. Helpers are common reagents (*e.g.*, water, oxygen) that react with starters, targets, and intermediates but not with other helpers.

The combined strategy integrates forward and retro expansion, allowing for more generations to be explored and enabling longer pathways. This hybrid approach improves computational efficiency while maintaining pathway diversity. Users can also customize strategies by running separate forward expansions from distinct feedstocks and merging the results with a retro expansion to maximize synthetic route diversity. Fig. 4 illustrates different expansion strategies.

Recent advances in retrosynthetic planning have introduced dynamic search strategies for constructing compact, task-specific subnetworks, such as Monte Carlo Tree Search (MCTS)^5^ and Retro*.^33^ Although DORAnet does not currently implement these strategies natively, users can emulate them by using DORAnet as a one-step reaction generator in combination with custom expansion strategies that determine which molecule to expand next during on-the-fly network generation. This approach mirrors the staged expansion workflows shown in Fig. 4(a) and enables more efficient exploration of large chemical spaces.

#### 3.2.2 Filters

DORAnet provides filtering mechanisms during network expansion to constrain network size, effectively mitigating combinatorial explosion. Various types of filters can be interjected at various points during the expansion, and multiple filters can be combined using logical operators. DORAnet includes several ready-to-use filters, which are listed in Table S2. Filter thresholds are user-defined, allowing users to tailor the filtering behavior to specific needs. The impact of filtering strategies on network growth and computational performance is demonstrated in Table S1.

Advanced users can also code custom filters, adding flexibility and customizability to DORAnet. For example, a filter could be designed to allow only reactions that produce smaller molecules or those where products meet specific property criteria. With Python programming and the RDKit package, a wide range of filter concepts can be implemented.

It is important to note that DORAnet does not include a built-in thermodynamic calculator. Users must integrate their own thermodynamic calculator that runs in a Python environment to utilize the thermodynamic filter. For the chemical/chemocatalytic reactions in this work, the authors estimated the standard enthalpy of formation using an in-house code based on the Benson group contribution method^34^ that is released here open-source, pathermo, on GitHub (see Data Availability). Pathermo includes the full set of C, H, O, N, S, and halogen groups used in this study, and can be used to reproduce the results presented here.

### 3.3 Post-processing

Once network expansion is complete, DORAnet applies post-processing to identify the most viable pathways through three steps: pathway search, ranking, and visualization.

#### 3.3.1 Pathway search

Pathway search systematically explores the reaction network to identify all distinct routes from the starting molecules to the target molecule. Instead of focusing on a single “optimal” pathway, the authors chose to obtain all possible pathways at this stage. This approach provides a comprehensive set of results, allowing for easy ranking and down-selection. It is particularly well-suited for offering multiple pathway options to experimentalists and others looking to implement novel pathways in practice, enabling them to choose from the top-ranked candidates.

While this is fundamentally a pathfinding problem, chemical reactions introduce additional complexity.^35,36^ Traditional pathfinding algorithms assume a single start and end node, with paths following only one branch at a junction rather than multiple branches simultaneously.^37^ In contrast, chemical reactions often involve multiple reactants, making the pathways leading to each reactant integral to the overall pathway. Consequently, a final path may contain forks and parallel sub-paths. Furthermore, when an intermediate can be produced through multiple reactions, each reaction forms a distinct pathway, leading to a combinatorial explosion of pathways. Fig. S1 illustrates the complexity of pathways in a reaction network.

DORAnet introduces a specialized backward pathfinding algorithm tailored for chemical reaction networks. The proposed algorithm leverages domain-specific heuristics to efficiently navigate non-linear, multi-reactant pathways. Starting from the target molecule, the algorithm recursively identifies reactions that can produce it and replaces the target with its precursor molecules, all while tracking the generation (reaction depth) of each species. The algorithm imposes generation limits, step limits, and atom economy thresholds (eqn (1)) to prune unpromising branches. A key innovation is the dynamic reordering of candidate nodes based on the number of available producers, which prioritizes the expansion of molecules that are synthetically challenging. This facilitates the early pruning of challenging intermediates, improving overall search efficiency. The algorithm combines breadth-first queue management with depth-first prioritization of recent branches. This hybrid strategy enables a more targeted and computationally efficient approach, well-suited to the complex, branched nature of chemical synthesis pathways. A high-level pseudocode of the pathway finder is presented in Algorithm 1 in the SI.1
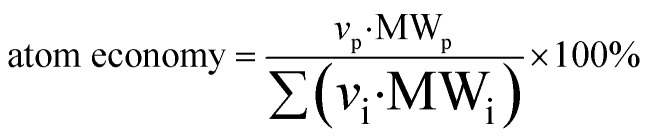
where MW_p_ represents the molecular weight of the desired product, *v*_i_ is the stoichiometric coefficient of species i, and ∑(*v*_i_·MW_i_) represents the sum of the molecular weights of all products. Cofactors involved in enzymatic reactions are excluded from this calculation, as they are typically recycled in biological systems.

#### 3.3.2 Pathway ranking

Pathway ranking is a key feature that distinguishes DORAnet from similar tools. It evaluates pathways based on multiple criteria to prioritize feasible, and what can be deemed more industrially relevant, routes, based on feedback into the ranking criteria from industrial partners. The current criteria include:

##### 3.3.2.1 Thermodynamics

Pathways are evaluated based on the maximum thermodynamic change (*e.g.*, standard gas-phase enthalpy of reaction) of the individual reactions within a pathway. This serves as an indicator of thermodynamic feasibility: pathways containing thermodynamically challenging reactions are considered less viable.^38^ Those with lower maximum enthalpy or free energy changes are ranked higher.

##### 3.3.2.2 Number of steps

Although shorter pathways are not always the most efficient, when assessing novel pathways, simpler pathways with fewer steps are ranked higher based on assumed superior economic and practical feasibility.

##### 3.3.2.3 Pathway by-product number

This criterion estimates the number of by-products in a chemical/chemocatalytic pathway. Fewer by-products indicate more efficient and selective pathways. To compute this, all feedstocks, intermediates, and products within a pathway are merged into a “soup,” and a one-generation expansion using all operators of interest is performed to determine the by-product number, which is the sum of the total number of products that can be formed from all species in the pathway. Pathways with lower by-product numbers are ranked higher. In our practical applications this criterion has only been applied to chemical/chemocatalytic reactions, as enzymatic reactions are inherently more selective,^25^ but the concept can be equally applied to enzymatic reactions as well to uncover or prevent underground metabolism.

During this calculation, another index, the intermediate by-product number, is also computed. Each intermediate within a pathway is allowed to react with other molecules in the “soup” for one generation, while reactions among other molecules are prohibited. The intermediate by-product number reflects the number of newly generated molecules in this expansion. While not used for ranking, it serves as an indicator of the diversity and reactivity of intermediates.

##### 3.3.2.4 Atom economy

Pathways with higher atom economies are preferred as they indicate more efficient use of reactants.

##### 3.3.2.5 Salt score

Indicates whether salt will be generated. Since salt formation complicates downstream processing and is generally undesirable,^39^ pathways that produce fewer salts are ranked higher. Currently, salt production is flagged only for nitrogen and sulfur chemical/chemocatalytic rules, while other reaction rules do not yet support this criterion.

##### 3.3.2.6 Reaxys hits

Represents the percentage of reactions reported in the Reaxys database, with higher percentages leading to higher rankings. However, since this criterion favors established reactions over novel reactions, it should be weighted less if innovation is a priority.

To apply this criterion, a Reaxys subscription and some manual operations are required. The pathway search function generates a Reaxys batch file containing all reactions in the pathways, along with an empty CSV file to store the search results. The batch file is submitted to Reaxys, which returns a result log viewable on the Reaxys website. The log is then copied and pasted into the result CSV file. Both the batch and result files are required for DORAnet to incorporate Reaxys hits into pathway rankings.

##### 3.3.2.7 Coolness

Users can assign weights to specific reaction types, favoring or penalizing certain pathways. Positive weights promote desired reactions, while negative weights discourage undesirable ones.

##### 3.3.2.8 Profitability index (PI)

Defined as:2
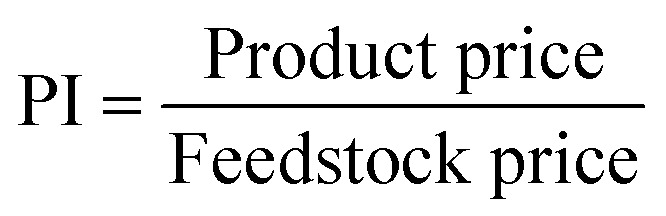


This provides a preliminary metric for profit, independent of operational costs. To use PI for ranking, users must provide a CSV file containing feedstock and product prices. This approach allows users to input context-specific pricing data, which may vary by region, market conditions, or application.

The scores for each criterion are normalized, and user-defined weights are applied to compute the final score for each pathway, allowing customization based on project goals.

For ranking criterion j, pathway i has a normalized score:3
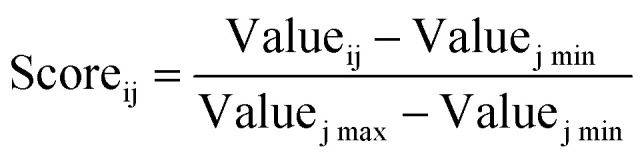
where Value_ij_ represents pathway i's value for criterion j, and Value_j max_ and Value_j min_ are the maximum and minimum values across all pathways for that criterion.

The total score for pathway i is computed as:

4 Score_i total_ = ∑(Score_ij_·Weight_j_)

where Weight_j_ is the ranking weight assigned to criterion j.

**Fig. 5:**
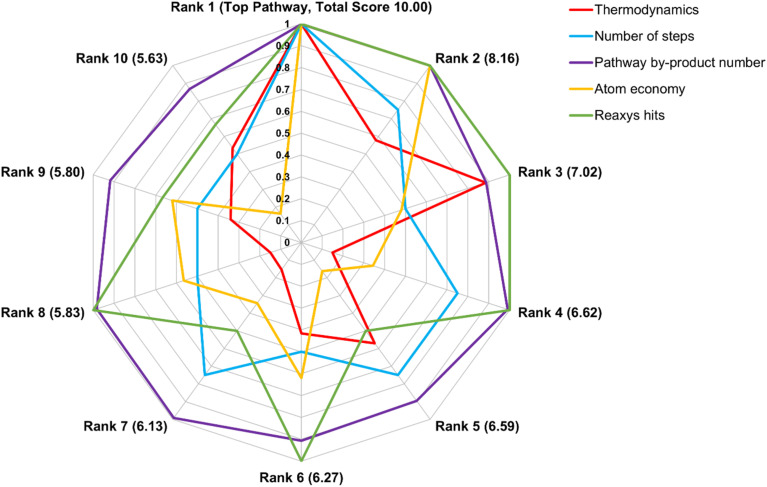
Total score (shown beside the rank number) and individual criterion scores for the top 10 pathways from renewable feedstocks (see SI) to propylene glycol. While high scores in some criteria can compensate for lower scores in others, the highest-ranking pathways generally achieve strong performance across multiple categories, making them more favorable. Ranking weights: thermodynamics (2), number of steps (4), pathway by-product number (2), atom economy (1), salt score (0), Reaxys hits (1), coolness (0), profitability index (0).

The total score determines the final pathway ranking, with weighting choices directly influencing the outcome. Because the total score reflects contributions from normalized scores across multiple metrics, an exceptionally high or low score in a single metric has a reduced impact. A sensitivity analysis (discussed below) examines the impact of different weight assignments. Fig. 5 illustrates pathway scores and rankings in an example case. This comprehensive ranking approach ensures robustness and practical relevance, enabling researchers to efficiently identify the most promising pathways.

#### 3.3.3 Pathway visualization

The final step in post-processing is pathway visualization. All ranked pathways are represented as graphs, providing an intuitive overview of each route. These visualizations display key information, including rank number, atom economy, by-product number, profitability index, reaction names, thermodynamic changes for each step, and Reaxys report status (with reported reactions highlighted in blue or a user-defined color). Pathways are stored in a PDF file for easy sharing and review, enabling researchers to efficiently assess their viability for experimental validation. A visualization example is provided in Fig. S7.

## 4. Results and discussion

### 4.1 Validation of reaction rules

The chemical/chemocatalytic reaction rules in DORAnet were validated against the reactions present in the USPTO-50 k dataset^40^ to ensure accuracy, diversity, and coverage. The USPTO-50 k dataset is a well-established benchmark, containing 50 000 diverse reactions extracted from patent literature through advanced text mining. To ensure a meaningful validation process, the dataset underwent rigorous sanitization to eliminate imbalanced reactions and reactions involving reacting sites outside of our scope (*i.e.*, reacting sites not involving C/H/O/N/S). This sanitization ensured that only well-defined and chemically plausible organic reactions were tested, while excluding problematic records resulting from the text mining process.

As summarized in Table 1, the DORAnet chemical/chemocatalytic rule set successfully reproduced 96.9% of the recorded reactions. This high level of coverage indicates that the rule set is comprehensive and capable of capturing a broad spectrum of commercially and synthetically relevant transformations. This gives DORAnet significant versatility, enabling it to explore complex chemical spaces that include a wide range of functional groups and reactivity types. A benchmark with other CASP tools is provided in Table S3.

**Table 1:** Validation of chemical/chemocatalytic reaction rules against USPTO-50k^a^

Atom types featured in reaction	Number of sanitized reactions	Reproduction rate
C/H/O only	9373	97.0%
Nitrogen	21 777	96.7%
Sulfur	1211	99.5%

a Note: all reaction types include reactions containing halogens. A reaction is considered successfully reproduced if any predicted product set matches the recorded products.

A detailed investigation into the performance of enzymatic reaction rules is presented in the work of Ni *et al.*,^14,30^ which introduced two rule sets: JN1224min and JN3604imt. JN1224min is a more generalized rule set designed to cover the most common enzymatic reactions while minimizing redundancy. In contrast, JN3604imt comprises a larger set of rules with optimized intermediate specificity, offering more realistic promiscuity and improved predictive accuracy. It covers 87% of all common enzymatic reactions^30^ from MetaCyc, KEGG, and BRENDA.^41–43^ JN3604imt is integrated into DORAnet as the default enzymatic rule set.

### 4.2 Chemical space exploration

One of the key applications of network expansion tools is their ability to explore vast chemical spaces and potentially discover novel molecules with unique properties. This capability is critical for the development of new bioactive compounds, materials, or chemical intermediates, where unique structural features can lead to desirable properties.^44^ This section evaluates DORAnet's performance in generating novel compounds.

The three-generation chemical/chemocatalytic reaction network from Section 3.2.1, initiated with alanine and six helper molecules, was used for this analysis. The network is visualized in Fig. S2. The third-generation molecules are not displayed due to their large quantity, which makes visualization in print impractical.

**Fig. 6:**
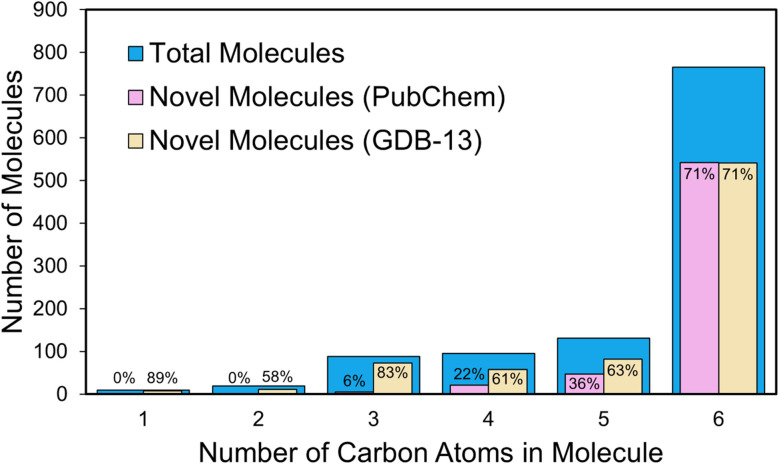
Analysis of the molecules with fewer than seven carbon atoms obtained from the reaction expansion of alanine, using six helper molecules and three generations of expansion. Blue bars represent the number of generated molecules containing a given number of carbon atoms. Purple and yellow bars indicate the number of these molecules not reported in PubChem and GDB-13, respectively, and are thus considered novel. The percentage of the total number of molecules with a given number of carbon atoms that are novel is shown over each purple and yellow bar.

To evaluate the novelty of the discovered molecules, they were compared against the PubChem^45^ and GDB-13 (ref. 46) databases. A molecule was classified as novel with respect to a given database if it was not found in that database, indicating unexplored chemical space. Fig. 6 shows the total number of generated molecules and the number of novel molecules after three generations of expansion, categorized by carbon atom count. To keep the analysis manageable, only molecules with fewer than seven carbon atoms and no more than 12 heavy atoms were investigated.

For PubChem, none of the molecules containing one or two carbon atoms were novel (0%), indicating that small molecules are well represented in the database. This observation is expected, as smaller molecules are typically well studied, leaving fewer opportunities for new structural variations. As molecular complexity increased, the proportion of unreported structures rose, demonstrating a clear and expected trend: larger molecules are increasingly likely to be novel. This demonstrates quantitatively that structurally diverse, larger molecules offer great potential for discovering new chemical entities. For six-carbon molecules, the majority were novel, outnumbering previously reported structures. For GDB-13, DORAnet consistently found a high percentage of novel molecules across all carbon counts.

These results demonstrate that DORAnet can explore chemical space to generate novel molecules, particularly as molecular complexity increases. This capability makes it a valuable tool for researchers aiming to discover new compounds in underexplored areas of chemical space.

### 4.3 Advantages of hybrid strategy

One feature of DORAnet is its ability to integrate both chemical/chemocatalytic and enzymatic rules, enabling the discovery of hybrid pathways. This section highlights the advantages of hybrid strategies by comparing molecules generated from hybrid networks to those produced through purely chemical/chemocatalytic or purely enzymatic expansions.

**Fig. 7:**
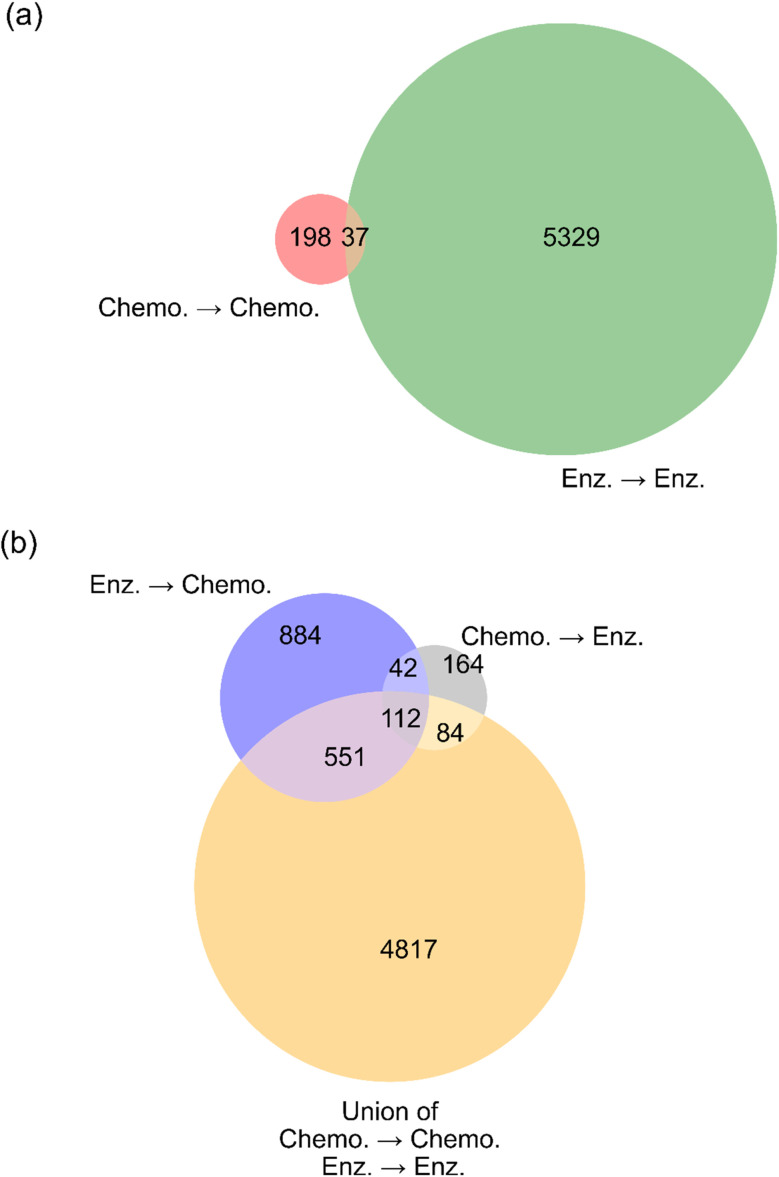
Venn diagrams illustrating the number of molecules produced by two-generation expansions with different strategies. “Chemo.” refers to chemocatalysis, “Enz.” refers to enzymatic catalysis. (a) Compares the molecules generated from two steps of either chemocatalysis (red) or enzymatic catalysis (green), with a small number (37) of molecules synthesized using either approach. (b) Compares the results in (a) to those of two hybrid options, in which one step of chemocatalysis is followed by one step of enzymatic catalysis, or one step of enzymatic catalysis is followed by one step of chemocatalysis.

The chemical/chemocatalytic reaction network from Section 3.2.1 was reused for this comparison. Additional networks were generated using alternative strategies: a two-generation enzymatic expansion, a one-generation enzymatic expansion followed by one-generation chemical/chemocatalytic expansion, and a one-generation chemical/chemocatalytic expansion followed by one-generation enzymatic expansion. The enzymatic expansion incorporated a thermodynamic filter using eQuilibrator,^47^ applying a threshold of Δ_r_*G*′° < 0 kcal mol^−1^ under default aqueous conditions (25 °C, 1 bar pressure, pH 7.5, ionic strength 250 mM, and pMg 3). The results are visualized in Fig. 7 using Venn diagrams.

Fig. 7(a) compares molecules generated from purely chemical/chemocatalytic and purely enzymatic expansions, showing that these approaches explore largely distinct regions of chemical space. The minimal overlap suggests that chemical/chemocatalytic and enzymatic transformations target fundamentally different reaction pathways. The larger number of molecules generated from enzymatic expansion is partly due to the much larger size of the enzymatic rule set, but also reflects the use of generalized reaction rules that model enzyme promiscuity, the potential for enzymes to act on structurally similar substrates beyond those they are experimentally known to accept.^19^

Fig. 7(b) demonstrates that hybrid expansions yield novel molecules that cannot be accessed through purely chemical/chemocatalytic or enzymatic routes alone. Both hybrid expansion strategies produced a significant number of unique molecules, highlighting the advantage of integrating both reaction types. This finding demonstrates hybrid strategies as a valuable feature of DORAnet, expanding accessible chemical space and providing new routes to target molecules. Moreover, hybrid pathways may offer attractive synthetic alternatives by leveraging enzymatic transformations for selective reactions and chemical/chemocatalytic steps for challenging conversions.

### 4.4 Sensitivity analysis

As discussed in Section 3.3.2, pathway ranking weights influence the final results. To understand the impact of varying ranking weights, a sensitivity analysis was conducted. Key ranking criteria analyzed included the number of steps, reaction thermodynamics, by-product number, atom economy, and Reaxys hits. Response Surface Methodology (RSM)^48^ was employed to systematically investigate the effects of altering weights across these five parameters.

Pathways from bio-based building blocks to propylene glycol were used as a test case. Propylene glycol was selected as the benchmark because it is a high-volume commodity chemical, with U.S. consumption exceeding 0.5 million metric tons annually.^49^ It is used across a wide range of industries, including pharmaceuticals, as a polyethylene terephthalate (PET) additive, and in other industrial applications.^50^ Moreover, its conventional three-step production route (fossil fuels to propylene to propylene oxide to propylene glycol^51^) offers multiple opportunities to introduce novel reactions at each stage.

**Fig. 8:**
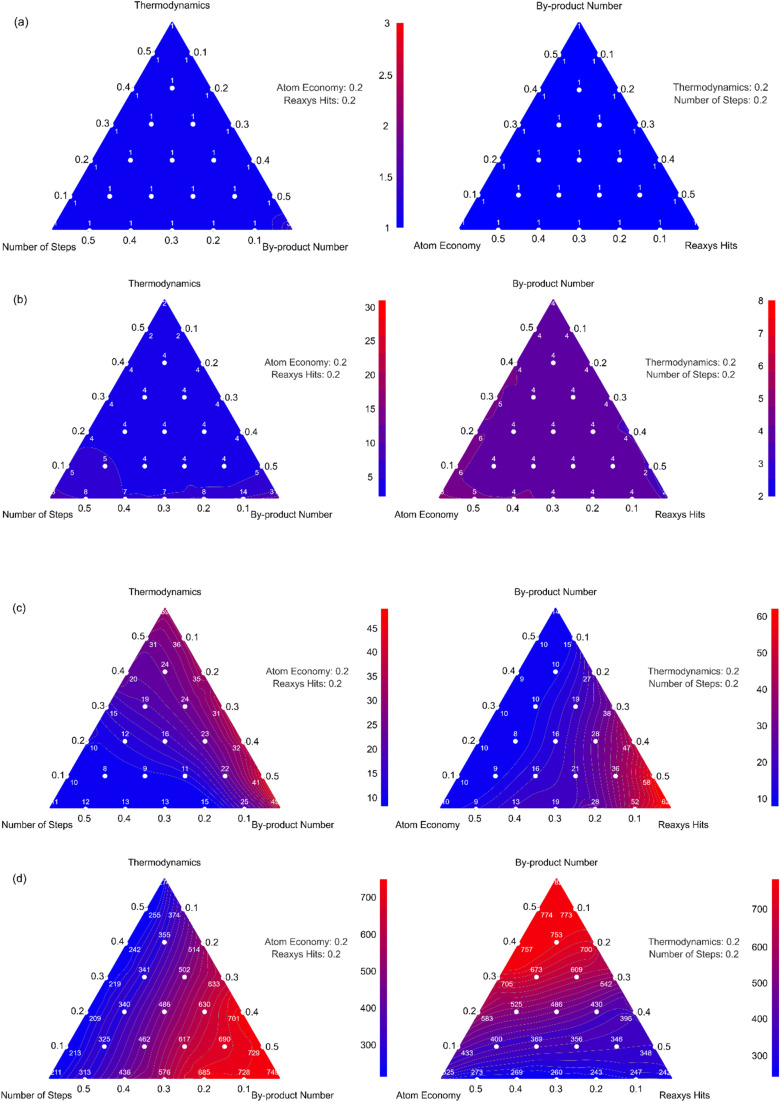
Response surface of rank of selected pathways in relation to ranking weights. (a)–(d) show the response surfaces for pathways ranked 1, 5, 10, and 500, respectively, according to the baseline ranking weights.

A baseline ranking was established using default weights (number of steps: 0.4, reaction thermodynamics: 0.2, by-product number: 0.2, atom economy: 0.1, Reaxys hits: 0), and the top 10 pathways are visualized in Fig. S7. Four pathways (Ranks 1, 5, 10, and 500) were selected for analysis. To explore combinations of the five factors, a grid sampling approach was used, where weight values were varied in increments of 0.1, and the total sum was fixed to 1. Note that there is no requirement that the weight values sum to a particular value since the rankings are recalculated in each case, and it is the values of the weights relative to each other that is important. With the rankings of the selected pathways recalculated for each combination, changes in the relative positions of the four pathways used for evaluation were tallied. Since a ternary plot can only accommodate three parameters at a time, separate response surfaces were generated for each pathway. Fig. 8 presents the response surfaces for different weight combinations.

The high-ranking pathways demonstrated strong stability, with only moderate rank fluctuations near the boundaries of the weight space. This suggests that high-ranking pathways are generally resilient to moderate shifts in weighting. In contrast, pathway 500 was highly sensitive to weight changes, reflecting a reliance on one or two favorable metrics. Such pathways are less competitive overall and more susceptible to ranking shifts.

Overall, the sensitivity analysis confirms the robustness of DORAnet's ranking algorithm, which consistently identifies promising pathways for experimental validation and industrial application. While top-ranked routes offer both stability and broad applicability, the system also provides flexibility to explore edge cases through custom weighting schemes, supporting both general-purpose and specialized use cases.

### 4.5 Case study

To demonstrate DORAnet's capability to identify industrially-relevant synthesis pathways, we present selected results from a broader project aiming to identify viable pathways between alternative feedstocks and 51 high-volume organic chemicals. Alternative feedstocks were selected based on their U.S. availability,^52^ compositional similarity to target chemicals (*i.e.*, carbon, oxygen, and heteroatom content), and lack of existing waste management strategies. Each feedstock class was assumed to undergo a representative pretreatment process to convert it into one or more chemically tractable starting molecules (*e.g.*, syngas, sugars, VFAs), which then serve as inputs to downstream chemical production pathways. DORAnet was employed to search for candidate pathways, offering insights into alternative routes and feedstocks. In this case study, only chemical/chemocatalytic reaction rules were used. This choice reflects the project's focus on the petrochemical sector by identifying drop-in or near-term alternatives to existing industrial processes, which predominantly rely on chemocatalysis.

We focused on converting various alternative feedstocks into target chemicals (see SI). Each feedstock is represented by one or more molecular structures, which serve as starting molecules for DORAnet. Notably, there is an overlap between alternative and petrochemical feedstocks. For example, syngas can be produced *via* steam methane reforming or biomass gasification.^53^ Ethylene is commercially synthesized through the thermal cracking of petroleum hydrocarbons, but can also be derived from waste plastic pyrolysis.^54^ As a result, commercially established pathways may appear in the results and offer validation of DORAnet's performance.

Target chemicals are categorized into four tiers based on their production hierarchy in the chemical industry. Tier 1 consists of platform chemicals or fundamental building blocks, such as ethylene. Tier 2 includes chemicals derived from tier 1 compounds, such as ethylene oxide from ethylene.^51^ Tier 3 comprises advanced or specialized products synthesized from tier 2 intermediates, such as ethylene glycol from ethylene oxide.^51^ Finally, tier 4 includes products derived from tier 3 compounds. This hierarchical structure reflects the progression of chemical synthesis from basic feedstocks to end-use compounds. Consequently, tier 1 chemicals also serve as feedstocks for higher-tier compounds during network expansion. If a target chemical has multiple isomers, all industrially-relevant isomers are considered in the analysis.

Using DORAnet, one generation of chemical/chemocatalytic forward expansion and three to five generations of chemical/chemocatalytic retro expansion were performed, and the combined networks were analyzed to identify viable pathways. A reaction enthalpy filter with a 15 kcal mol^−1^ threshold was applied during expansions to eliminate enthalpically unfavorable reactions. Default weights were used for pathway ranking (number of steps: 0.4, reaction thermodynamics: 0.2, by-product number: 0.2, atom economy: 0.1). Greater weight was assigned to number of steps, as shorter pathways enhance efficiency and yield,^55^ which are critical in industrial applications.

**Fig. 9:**
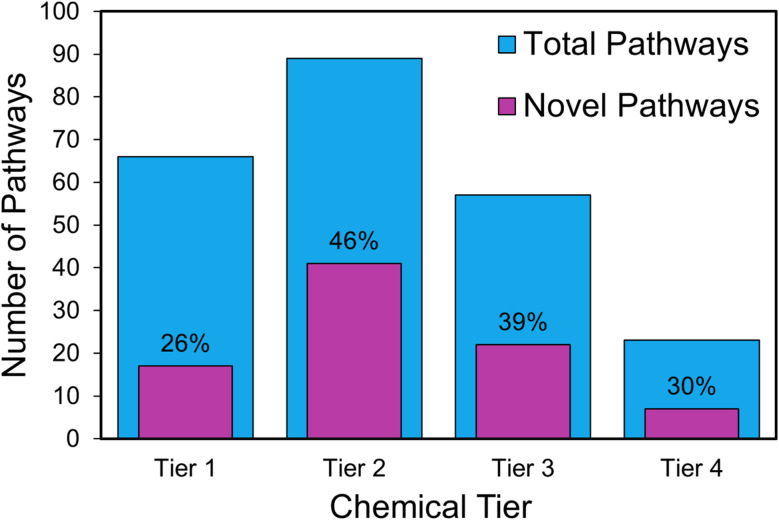
Number of novel pathways identified by DORAnet across chemical tiers. For each target compound, pathways starting from the same feedstock were grouped to simplify comparison. Blue bars represent the total number of pathways considered, including those reported in the literature and the top-25 ranked DORAnet pathways of each target. Purple bars indicate the pathways uniquely identified by DORAnet. Percentages show the fraction of novel pathways within each tier.

For each target molecule, DORAnet generated a ranked list of pathways. To evaluate its ability to discover novel routes, a literature review was conducted to collect all experimentally reported pathways associated with each target. We then compared these with the top 25 pathways identified by DORAnet. The results, summarized in Fig. 9, show that a substantial fraction of pathways were uniquely identified by DORAnet, particularly for tier 2 and tier 3 targets. This suggests that DORAnet is especially effective at generating new synthetic strategies for intermediate-level chemicals, likely because these targets are downstream enough to allow structural diversity, yet not so complex as to require specialized reactions that might limit pathway options. The relatively lower novelty in tier 1 is expected, as these platform chemicals are extensively studied and well-documented. The low number of total pathways and moderate novelty observed in tier 4 suggests that more complex products may inherently allow fewer alternative pathways, due to increased structural constraints and synthetic difficulty. Overall, it demonstrates DORAnet's ability to uncover previously unexplored pathways, complementing literature-based approaches.

**Fig. 10:**
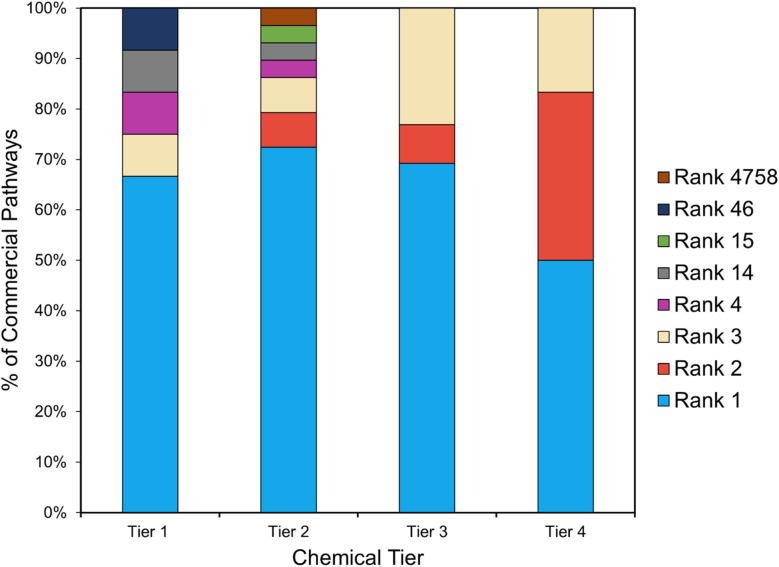
Rankings of commercial pathways in DORAnet results. Each bar represents a chemical tier, and each stacked segment indicates the percentage of commercial pathways that achieved a given rank. Notably, commercial routes frequently appear among the top-ranked pathways.

Next, the top-ranking DORAnet pathways were compared against current and historical commercial routes. The commercial routes for eight of the targets could not be reproduced by DORAnet due to outside constraints rather than gaps in DORAnet's methodology. The most common reason is that the commercial routes rely on petroleum-derived feedstocks that are absent from the alternative feedstock list. If such feedstocks cannot be derived from our available feedstocks, reproducing the commercial pathway becomes impossible. For example, the mainstream commercial pathway for 1,4-butanediol (the Reppe process)^51^ requires acetylene as a feedstock, which is absent from our list. In another case, acetone^51^ is co-produced with phenol in industrial processes. Reproducing such pathways requires adding the specific co-product as a starting molecule in the retro expansion, which falls outside the scope of our investigation. Lastly, lysine is commercially produced exclusively *via* fermentation^56^ and lacks synthetic routes. A summary of the reasons for the absence of commercial routes is provided in the SI. For the remaining targets, Fig. 10 summarizes the rankings of commercial pathways.

**Fig. 11:**
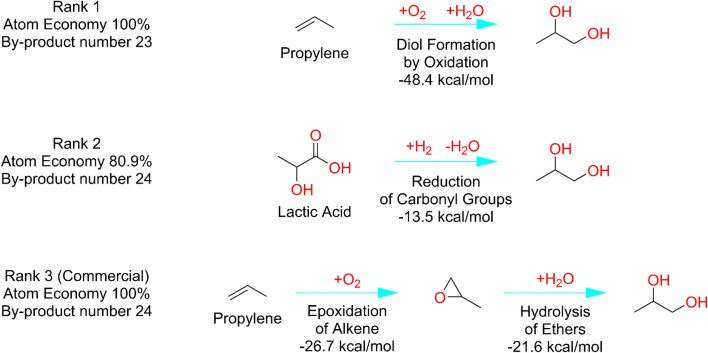
Top three ranked pathways to propylene glycol (commercial route ranked #3). Blue arrows denote chemical/chemocatalytic reactions reported in Reaxys. The #1 pathway closely resembles the commercial route and has been reported to achieve a 95% yield.^57^ However, it employs an expensive osmium catalyst, which may have limited its adoption in industrial applications.

Notably, although the project was not designed to reproduce commercial routes, DORAnet frequently identified these established pathways among the highest-ranked results, often placing them first or within the top three. For instance, the commercial route to propylene glycol was ranked #3 (see Fig. 11). This strongly indicates the reliability of DORAnet's ranking method and confirms that its metrics are well-calibrated for practical application. Additionally, DORAnet uncovered synthetic routes not documented in existing literature, showcasing its capability to explore novel pathways. Among tier 2 targets, an interesting outlier emerged: the commercial pathway for methionine was ranked #4758. This anomaly will be discussed in the following section.

#### 4.5.1 Pathway analysis

**Fig. 12:**
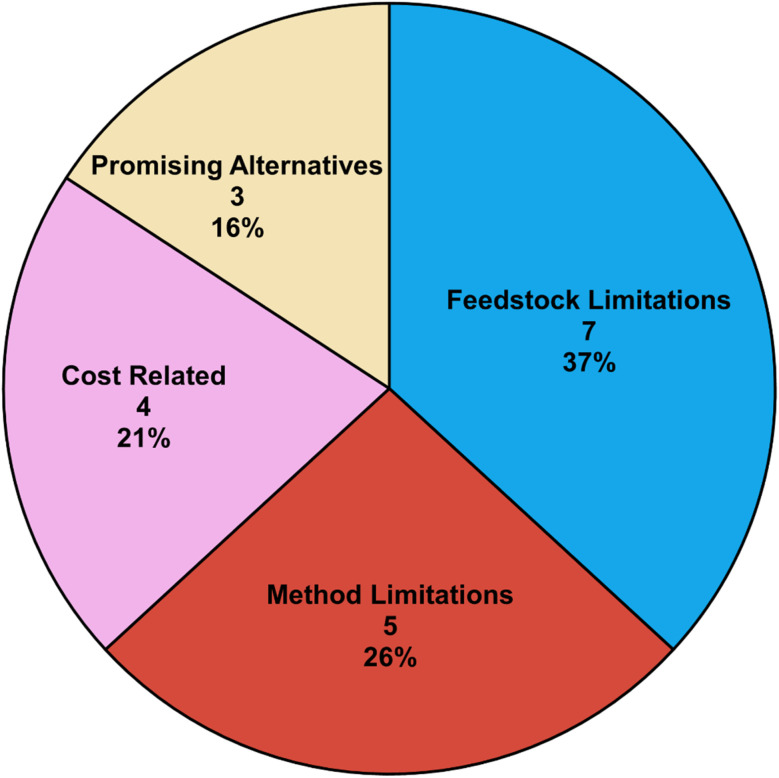
Factors contributing to commercial pathways not achieving the top rank.

A detailed analysis was conducted on cases where commercial pathways did not achieve top rankings. The underlying reasons were identified and classified into four main categories, as shown in Fig. 12. This analysis helps assess whether the ranking method has limitations, can be refined, or successfully identifies promising alternative pathways that outperform current commercial routes.

Feedstock limitations are the leading reason, occurring when required feedstocks are absent but can be generated. The reproduced commercial pathway becomes unnecessarily long because it must first generate the required feedstocks from available ones. If these key feedstocks were provided as starting molecules, the commercial pathways would likely achieve top rankings. This is not a failing of DORAnet but rather highlights the importance of aligning feedstock parameters with realistic industry scenarios. An example is illustrated in Fig. S3, which shows how providing more advanced feedstocks can significantly reduce the synthesis length for a complex target.

The second category consists of cases where DORAnet failed to rank the commercial pathway highly, while the top-ranked pathways proved problematic upon closer examination. These cases reveal DORAnet's limitations and warrant further investigation. The issues often stem from reactions that are difficult to perform, have low yields, or exhibit poor selectivity. Although these reactions are theoretically valid, they may not be practical for industrial application. Addressing these cases requires a case-by-case analysis to refine DORAnet's ranking methodology. However, even problematic pathways may offer insights that inspire the design of novel synthetic routes. Examples comparing top-ranking pathways with their commercial counterparts^51^ are illustrated in Fig. S4.

##### 4.5.1.1 Case 1: methylene diisocyanate (4th tier target)

The commercial route closely resembles the top-ranked pathway but follows a different reaction sequence. The commercial pathway was ranked #2 due to its higher by-product number. However, upon closer examination, the top-ranked pathway includes an unreported reaction (Friedel–Crafts hydroxyalkylation of nitrobenzene). This reaction is unlikely to occur because the electron-withdrawing nitro group deactivates the system, preventing the reaction.^31^ In contrast, the commercial pathway employs an electron-donating amino group, which promotes the reaction.

This case reveals a limitation in DORAnet's ability to assess the feasibility of seemingly valid reactions. While refining reaction rules to account for substituent effects could help with this specific case, similar unforeseen cases will always arise. The authors suggest that reaction rules should remain general rather than overly restrictive, as it is preferable to filter out infeasible reactions rather than risk missing viable ones. Implementing a reaction feasibility algorithm such as the one that we have developed for enzymatic reactions^10^ would be a valuable approach to addressing this challenge.

##### 4.5.1.2 Case 2: 1-butanol (2nd tier target)

The commercial pathway was ranked #4 primarily due to its longer reaction sequence. In contrast, the top-ranked pathway consists of a single step. However, this pathway follows an anti-Markovnikov addition, which is likely to suffer from poor selectivity. Cases like this prompted the later incorporation of a regioselectivity filter in DORAnet.

##### 4.5.1.3 Case 3: 2-ethylhexanol (2nd tier target)

The commercial pathway was ranked #15 due to its length. Notably, the final two steps in this route are both hydrogenation reactions, which, in industrial production, are performed in a single hydrogenation reactor.^51^ If these two steps were combined in DORAnet, the commercial pathway with its higher atom economy and lower maximum reaction enthalpy would receive a higher score and replace the current top-ranked pathway. As a future improvement, DORAnet's post-processing could integrate consecutive steps of the same reaction type.

The top-ranking pathway in this case also presents challenges. It contains two unreported reactions. While the final step (hydrolysis) appears reasonable, the preceding step (cross-coupling) relies on an uncommon reaction,^58^ which may also suffer from selectivity issues. Additionally, this pathway introduces chlorine, raising concerns regarding safety, environmental impact, and equipment corrosion. These challenges could be addressed during post-processing. For instance, increasing the weight of Reaxys hits and specifying unfavored reactions in cool reactions could penalize the top pathway, while excluding chlorine-containing feedstocks would prevent this route from appearing.

##### 4.5.1.4 Case 4: isobutanol (2nd tier target)

Similar to Case 2, the top-ranked pathway consists of a single step but follows an anti-Markovnikov addition, which may result in selectivity issues.

##### 4.5.1.5 Case 5: styrene (3rd tier target)

The commercial pathway was ranked #3 due to its high reaction enthalpy. The final ethylbenzene dehydrogenation step has a reaction enthalpy of 28.5 kcal mol^−1^. It requires typical operating conditions of 620 °C, which is significantly higher than most organic chemical production processes.^51^ Styrene can be considered a special case with an unusually high thermal barrier. The fact that it still ranked within the top three suggests that the ranking method is robust.

Similar to Case 3, the top-ranked pathway introduces unfavorable chlorine and contains one unreported reaction. Like Case 1, it could benefit from a feasibility algorithm to better assess reaction viability.

The third category involves cost-related challenges, which arise because feedstock and catalyst prices were not included in the ranking. As a result, the top-ranked pathway may rely on expensive feedstocks or catalysts. An example with acetic acid as the target is shown in Fig. S5. The top-ranked pathway has a significantly lower reaction enthalpy and was ranked higher than the commercial pathway (#2). However, its feedstock costs are substantially higher than those of the commercial route. The cost of feedstock was later incorporated into DORAnet as a ranking criterion. However, catalyst and operational costs are not yet accounted for, representing a potential area for future improvement.

In the final category, the top-ranked pathways emerged as promising alternatives that outperformed commercial pathways based on the ranking criteria. These results are particularly encouraging, as they suggest the potential for discovering superior pathways that have not yet been widely adopted, possibly due to factors such as industrial verification challenges or scale-up limitations. One example is illustrated in Fig. S6. However, the authors acknowledge that the ranking method remains constrained by consideration of a limited list of criteria, so evaluation by practitioners is necessary to assess the further viability of these pathways.

The case study demonstrates that DORAnet effectively replicates and validates established industrial processes. Again, the primary goal was to identify alternative pathways rather than prioritize commercial pathways, and the results confirm that most commercial routes are, not surprisingly, indeed highly favorable. While DORAnet excels at identifying optimal pathways based on predefined criteria, careful consideration of real-world constraints, such as feedstock availability, operational costs, regulatory constraints, and industrial scalability, is essential to bridging the gap between computational predictions and practical implementation.

#### 4.5.2 Hybrid pathways

To demonstrate DORAnet's capability to identify hybrid reaction pathways, this section presents hybrid routes to propylene glycol, a representative target chemical used earlier in this work. The hybrid pathways were generated by combining the propylene glycol chemical/chemocatalytic retro expansion network from the case study with a one-generation enzymatic forward expansion. Thermodynamic filtering was applied with a 15 kcal mol^−1^ reaction enthalpy threshold for chemical/chemocatalytic steps and a 0 kcal mol^−1^ free energy threshold for enzymatic steps. Pathways were ranked using the default criteria described previously.

**Fig. 13:**
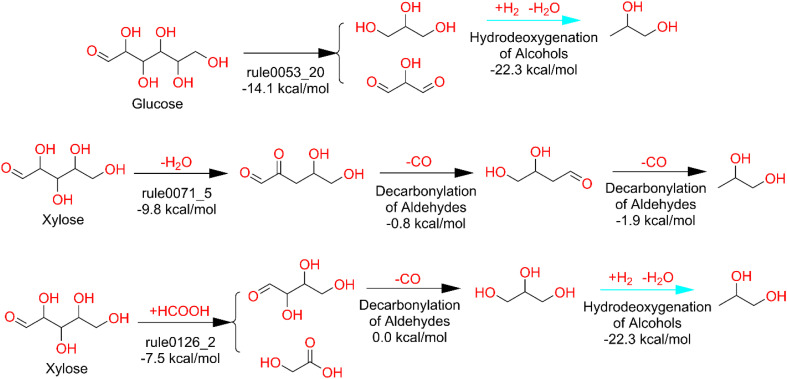
Hybrid pathways to propylene glycol. Pathways originating from sugar-type starter molecules were selected, as these compounds are readily utilized in biological systems (unlike molecules such as propylene, which are not naturally metabolizable despite their industrial importance). Each pathway begins with one enzymatic reaction (annotated with the reaction rule ID), followed by a series of chemical/chemocatalytic transformations (annotated by reaction type).

Fig. 13 presents three selected hybrid pathways ranked within the top 10% of results identified by DORAnet. These routes were not accessible through previous purely chemical/chemocatalytic expansions, reaffirming the value of hybrid strategies. While this section does not assess the experimental feasibility of the individual pathways, the results highlight DORAnet's ability to integrate and traverse distinct reaction domains within a single synthesis route. These findings suggest opportunities for future research into hybrid process design, where the combined use of enzymatic and chemical/chemocatalytic transformations could unlock access to otherwise unreachable molecules and diversify the range of viable synthetic strategies.

## 5. Conclusion

DORAnet is a powerful and versatile tool for (bio)synthetic route discovery, providing researchers and industrial chemists with a comprehensive platform to explore, analyze, and optimize chemical pathways. Through its modular architecture, extensive reaction rule database, and flexible network expansion strategies, it allows for the identification of novel synthetic routes that leverage the advantages of both chemical/chemocatalytic and enzymatic transformations. Furthermore, the open-source nature of DORAnet makes it highly adaptable, which is advantageous for researchers looking to customize the tool for specific projects or expand its capabilities for new types of chemistry.

In this work, we demonstrated the comprehensiveness and accuracy of DORAnet's reaction rules, its capability to generate novel molecules that are underrepresented in existing chemical databases, and DORAnet's ability to identify both established and alternative pathways for key industrial chemicals. The ranking methodology employed by DORAnet has proven to be effective in prioritizing practical and efficient pathways, aligning well with known industrial routes while also surfacing novel opportunities. While this work focuses on synthesis pathway discovery, DORAnet's flexible framework could also support applications such as environmental waste degradation and chemical recycling, broadening its relevance to a wide range of relevant chemistries.

Despite its strengths, challenges remain, particularly regarding the combinatorial explosion of network expansion and feasibility of predicted novel reactions, which call for an improved reaction feasibility assessment. Future improvements, including machine learning-based ranking refinements, helper molecules and prediction of co-products, improved thermodynamic calculations, and more accurate modeling of enzyme–substrate compatibility will further enhance the predictive power and usability of DORAnet. Parallelization is under development and planned for a future release, with the goal of significantly improving performance for large-scale network expansions. We also plan to implement a native priority queue–based expansion strategy to support A*-style^59^ exploration.

## Conflicts of interest

There are no conflicts to declare.

## Supplementary Material

DD-004-D5DD00229J-s001

DD-004-D5DD00229J-s002

## Data Availability

The version of DORAnet used in this study (v0.5.5a1) is archived on Zenodo (DOI: https://www.doi.org/10.5281/zenodo.17172561). The most recent release (v0.5.6a1) is archived on Zenodo (DOI: https://www.doi.org/10.5281/zenodo.17172622). The DORAnet source code is also available on GitHub (https://github.com/wsprague-nu/doranet) for ease of access. The interactive hands-on exercise is available *via* Google Colab (https://colab.research.google.com/drive/1Ms_bywzFFQOOdKtNO54_vWDtq2smtn2a?usp=sharing). The pathermo package is archived on Zenodo (DOI: https://www.doi.org/10.5281/zenodo.17172519) and is also openly available on GitHub (https://github.com/dmdqy/pathermo). A complete archive containing scripts and datasets used in this work is available on Zenodo (DOI: https://www.doi.org/10.5281/zenodo.17172591). Supplementary information: additional figures, tables, algorithm, scripts to reproduce the results, and case study feedstocks and targets are provided in the Supplementary Information (SI). See DOI: https://doi.org/10.1039/d5dd00229j.
