# Supplementary material for: Discovery of hybrid chemical synthesis pathways with DORAnet
Source: Digit Discov. 2025 Sep 26;4(11):3109–25. doi: 10.1039/d5dd00229j (PMC12512011; doi:10.1039/d5dd00229j)
Supplement: DD-004-D5DD00229J-s001 [file DD-004-D5DD00229J-s001.pdf]

Supplementary Information (SI) for Digital Discovery.
This journal is © The Royal Society of Chemistry 2025

# Supplementary Information for
# Discovery of Hybrid Chemical Synthesis Pathways with DORAnet

Quan Zhang[a], William W. Sprague[a], Shivani S. Kozarekar[a], Stefan C. Pate[a], Taylor Uekert[b], and Linda J. Broadbelt[a]

[a] Department of Chemical and Biological Engineering, Northwestern University, 2145 Sheridan Road, Evanston, Illinois 60208

[b] Strategic Energy Analysis Center, National Renewable Energy Laboratory, 15013 Denver West Parkway, Golden, Colorado 80401

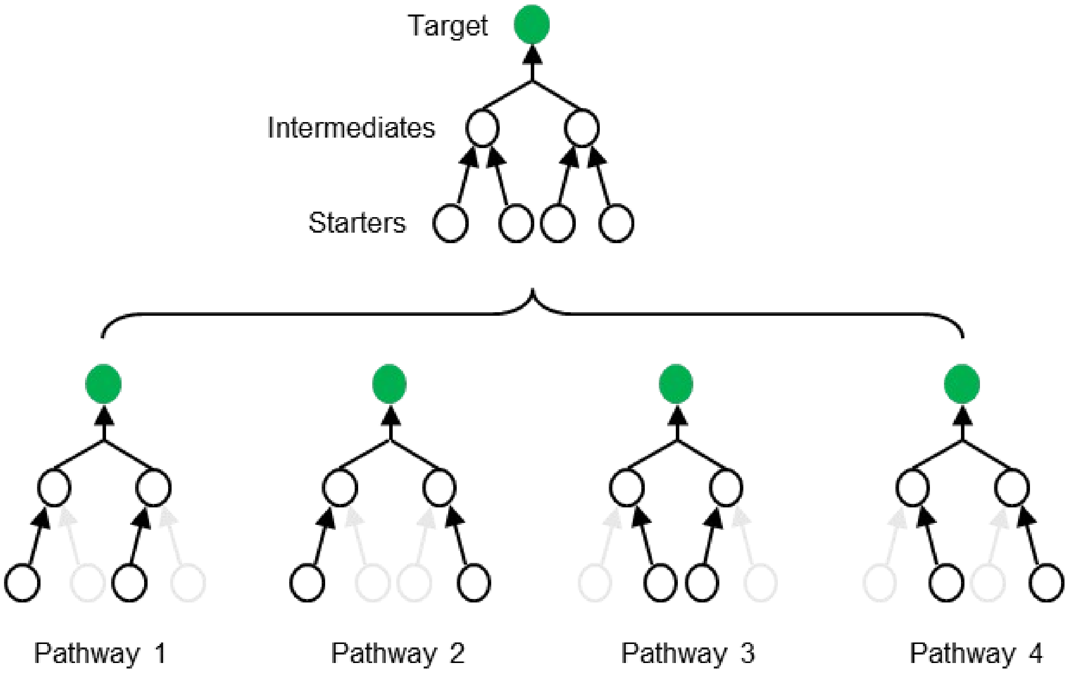


Fig. S1. Pathways from a simple two-generation reaction network. Nodes represent molecules, while edges represent reactions. The target is the product of a bimolecular reaction, meaning both intermediates must be produced through their respective pathways. Consequently, each pathway consists of three reactions, despite spanning only two generations.

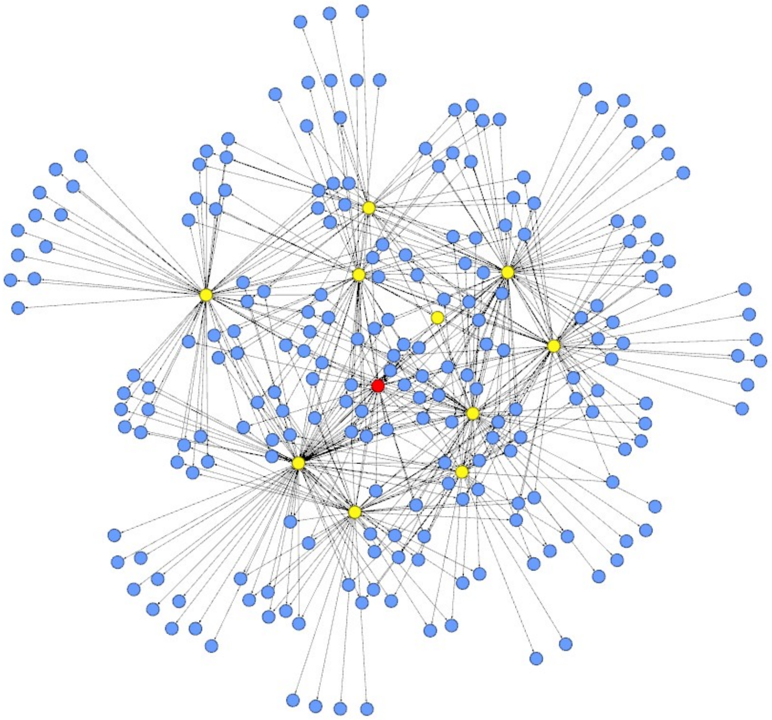

Fig. S2. Reaction network from alanine, displaying 0- to 2-generation molecules. The starting molecule (alanine) is marked in red, first-generation products in yellow, and second-generation products in blue.

Rank 1
Atom Economy 53.9%
By-product number 149

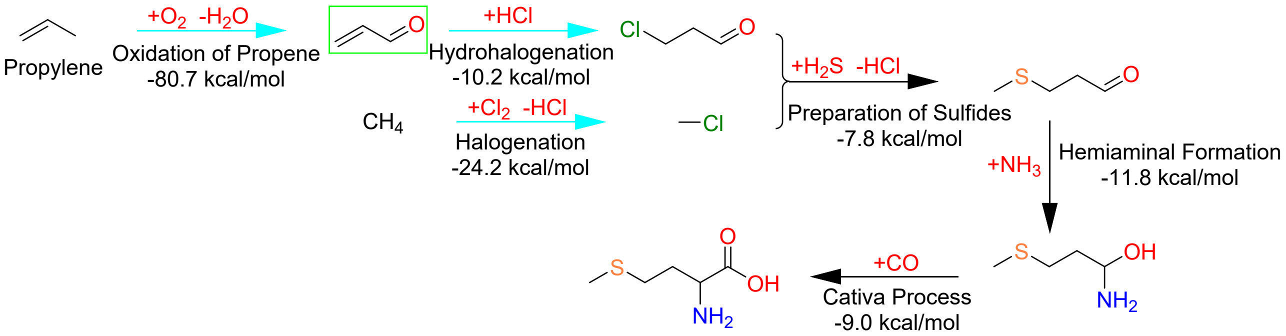


Rank 4758 (Commercial)
Atom Economy 40.0%
By-product number 186

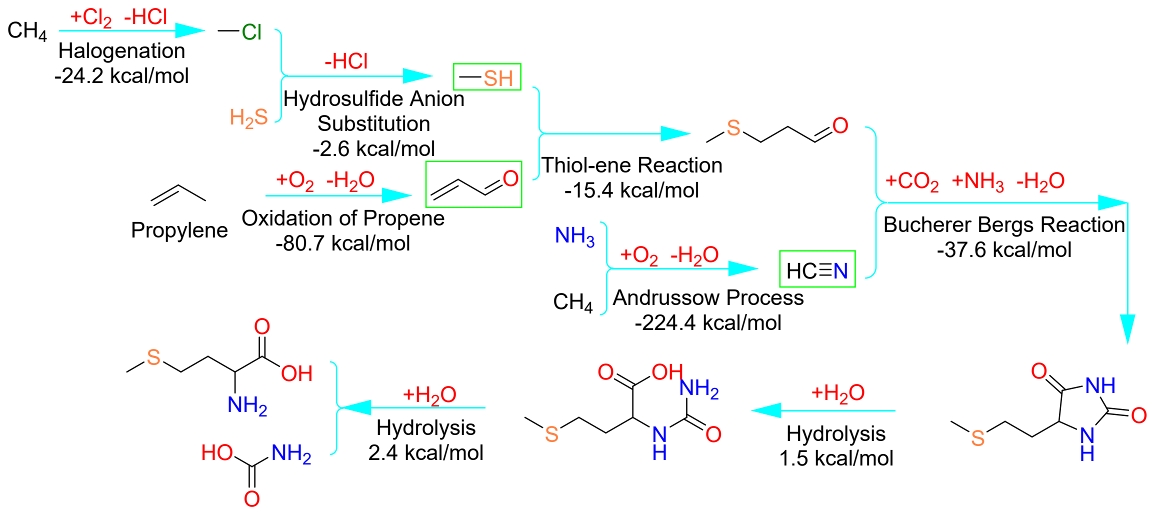


Fig S3. The top-ranking pathway and the commercial pathway for production of methionine[1]. Blue arrows represent reactions reported in Reaxys, while black arrows indicate unreported reactions. Green squares denote feedstocks used in commercial pathways. The commercial pathway ranked low due to its length (eight steps) compared to the six-step top pathway. However, in industrial practice, methanethiol, acrolein, and hydrogen cyanide are readily available as feedstocks. If these were provided as starting molecules, the commercial route would be reduced to four steps and rank as the top pathway. Notably, it is the only pathway composed entirely of reported reactions, meaning its ranking could improve if more weight were assigned to Reaxys hits. In this study, Reaxys hits were weighted at zero since the objective was to identify novel

pathways. It is also important to note that the top pathway contains three unreported reactions, including a key step utilizing the Cativa process, which is conventionally applied in acetic acid production[1] but has not been used for complex molecules. This reduces the pathway's credibility, presenting a challenge discussed in the following discussion.

(a) Methylene Diisocyanate

Rank 1
Atom Economy 47.9%
By-product number 146

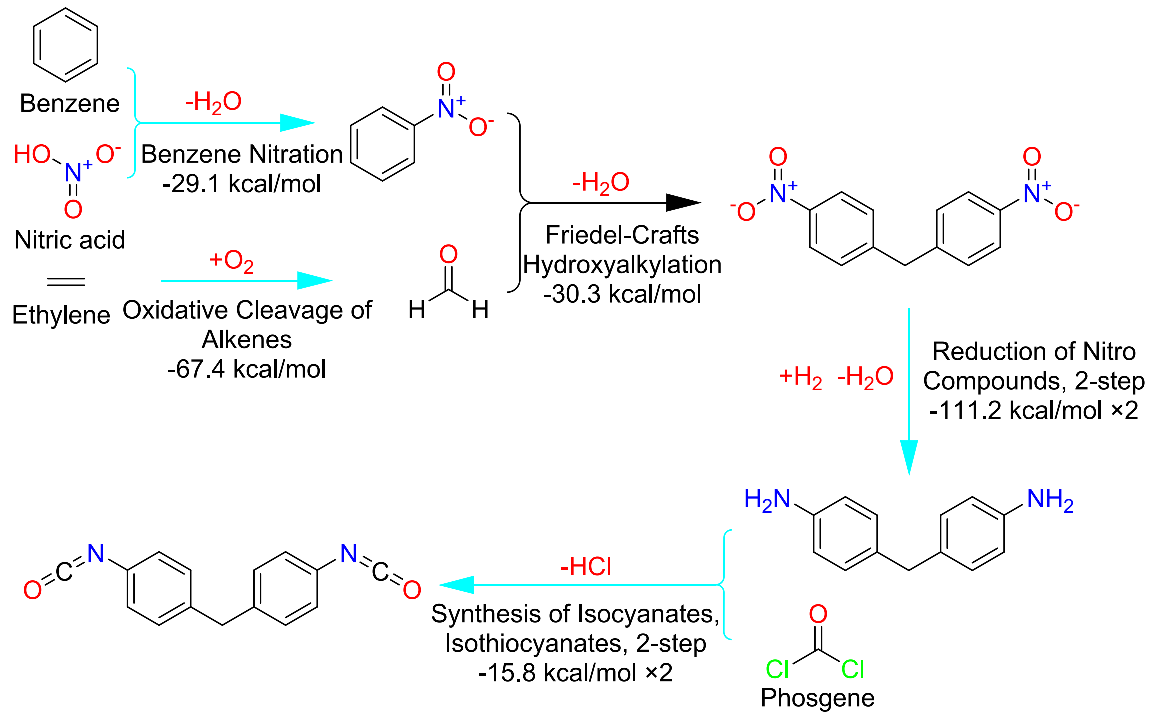


Rank 2 (Commercial)
Atom Economy 47.9%
By-product number 168

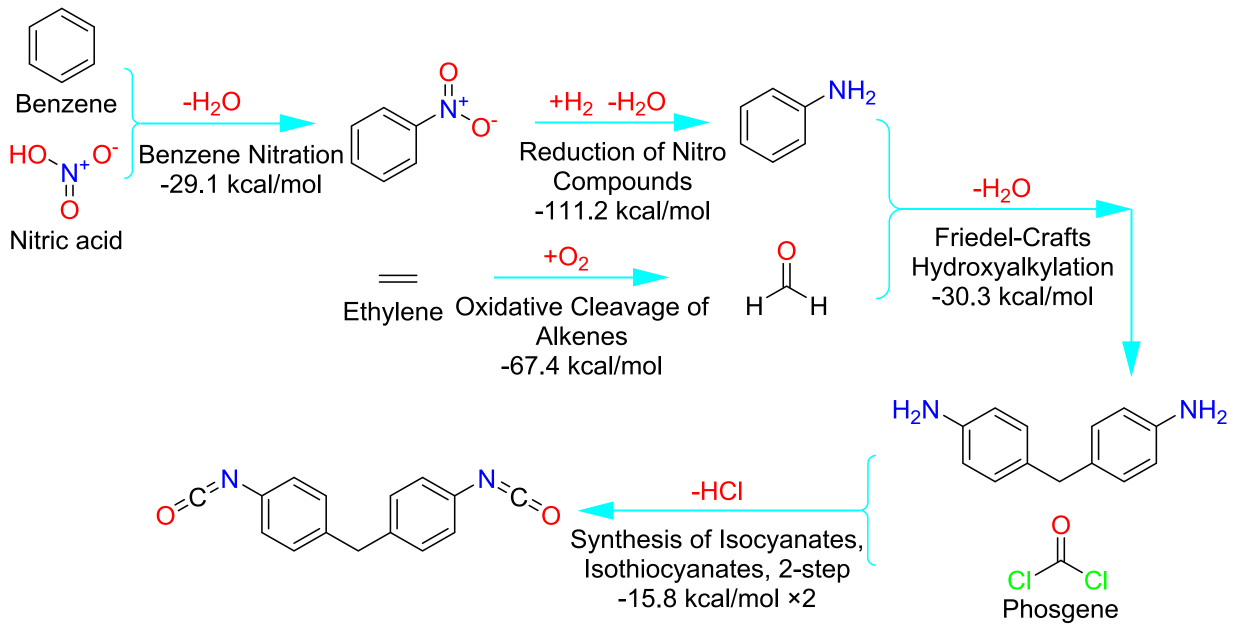

(b) 1-Butanol

Rank 1
Atom Economy 100%
By-product number 5

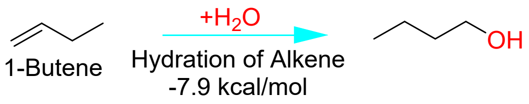


Rank 4 (Commercial)
Atom Economy 100%
By-product number 24

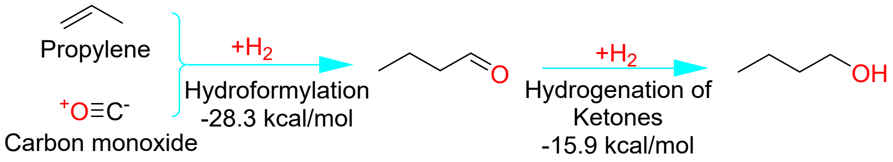


(c) 2-Ethylhexanol

Rank 1
Atom Economy 78.1%
By-product number 62

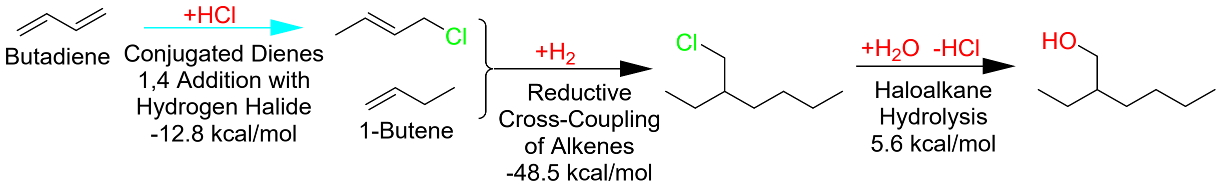


Rank 15 (Commercial)
Atom Economy 87.8%
By-product number 78

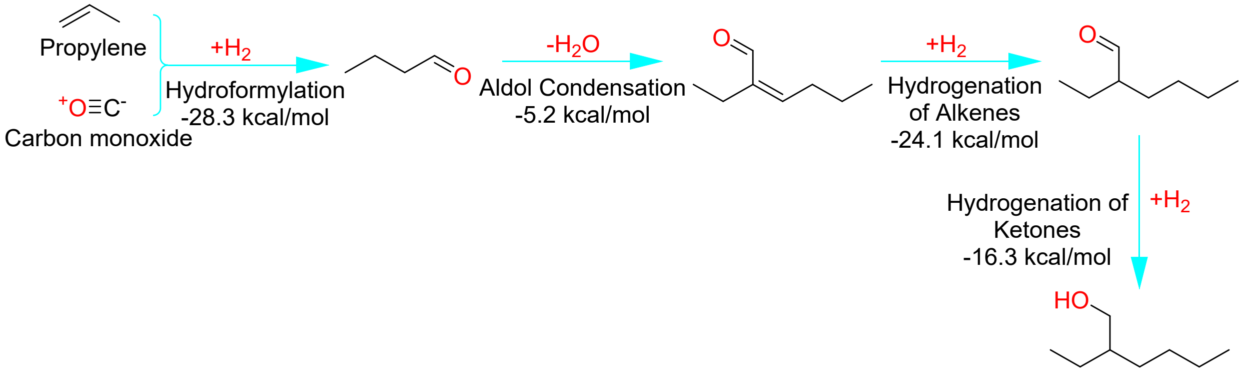


(d) Isobutanol

Rank 1
Atom Economy 100%
By-product number 5

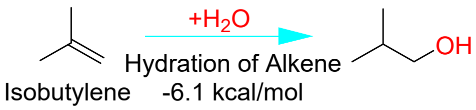


Rank 3 (Commercial)
Atom Economy 100%
By-product number 23

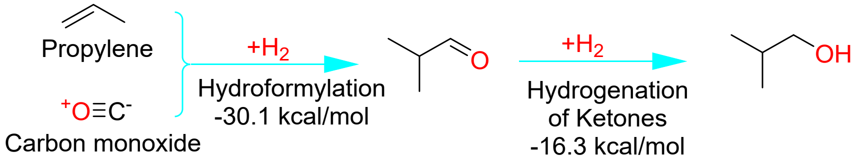

(e) Styrene

Rank 1
Atom Economy 58.8%
By-product number 51

Rank 3 (Commercial)
Atom Economy 98.1%
By-product number 66

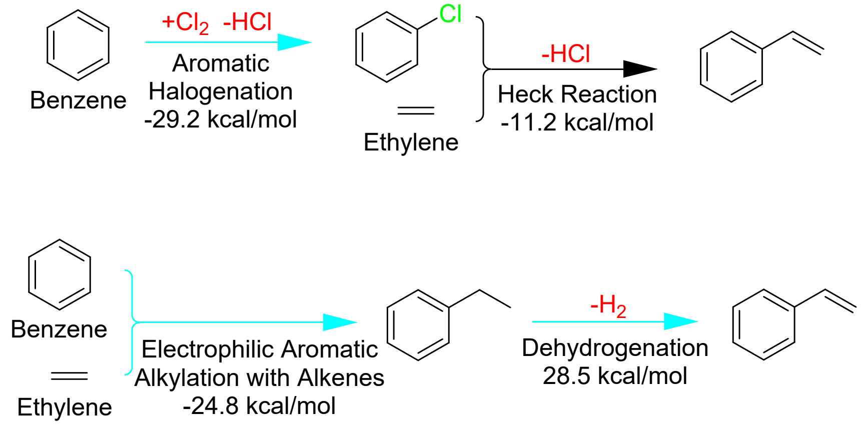


Fig. S4. Problematic top-ranked pathways and their commercial counterparts. (a) Methylene diisocyanate, (b) 1-Butanol, (c) 2-Ethylhexanol, (d) Isobutanol, (e) Styrene. The issues plaguing the top-ranked pathways are described in detail in the text.

Rank 1
Atom Economy 76.9%
By-product number 8

Rank 2 (Commercial)
Atom Economy 100%
By-product number 7

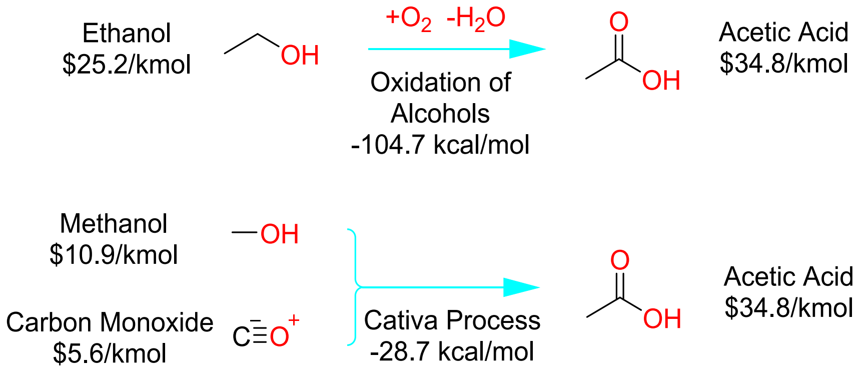


Fig. S5. Top-ranking pathways to acetic acid. The figure includes the 2014–2018 average prices of ethanol[2] and carbon monoxide[3], as well as the 2018 average prices of methanol[4] and acetic acid[5].

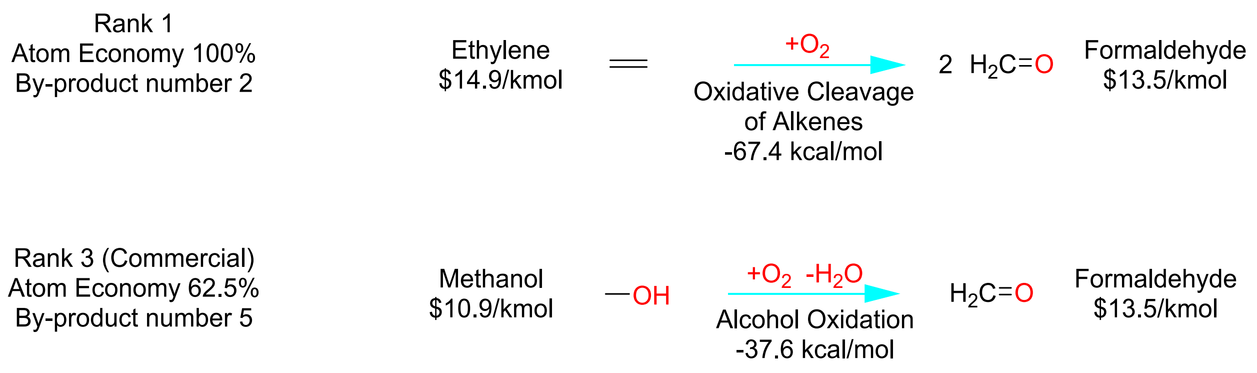


Fig. S6. Top-ranking and commercial pathways to formaldehyde. The figure includes 2018 average prices for ethylene[6], methanol[4], and formaldehyde[7]. The top-ranked pathway offers higher atom economy, lower cost (each ethylene molecule produces two formaldehyde molecules), and has been reported to proceed with various oxidants and catalysts[8-10].

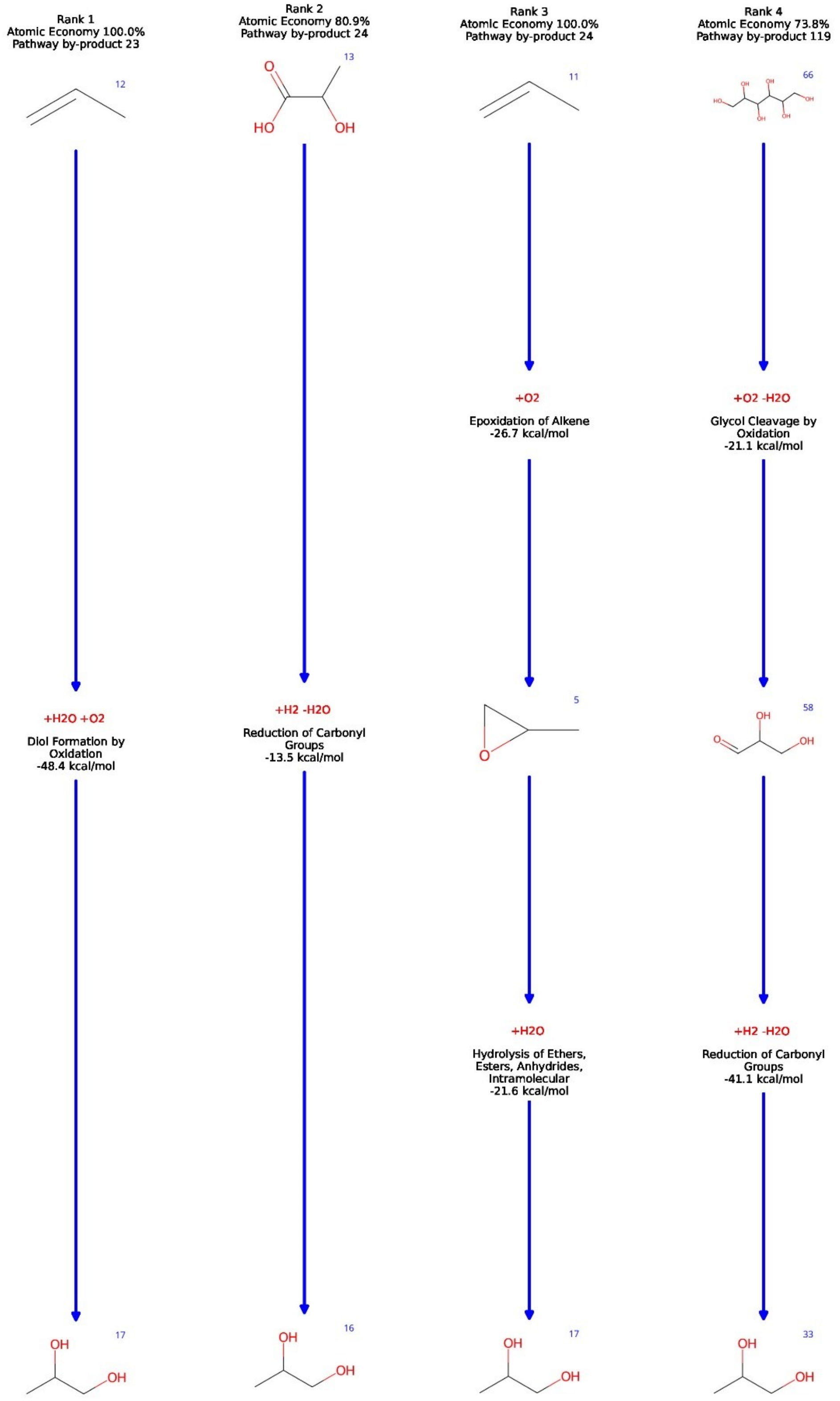

Rank 1
Atomic Economy 100.0%
Pathway by-product 23
Rank 2
Atomic Economy 80.9%
Pathway by-product 24
Rank 3
Atomic Economy 100.0%
Pathway by-product 24
Rank 4
Atomic Economy 73.8%
Pathway by-product 119
12
13
11
66
+O2
Epoxidation of Alkene
-26.7 kcal/mol
+O2 -H2O
Glycol Cleavage by Oxidation
-21.1 kcal/mol
+H2O +O2
Diol Formation by Oxidation
-48.4 kcal/mol
+H2 -H2O
Reduction of Carbonyl Groups
-13.5 kcal/mol
5
58
+H2O
Hydrolysis of Ethers, Esters, Anhydrides, Intramolecular
-21.6 kcal/mol
+H2 -H2O
Reduction of Carbonyl Groups
-41.1 kcal/mol
17
16
17
33

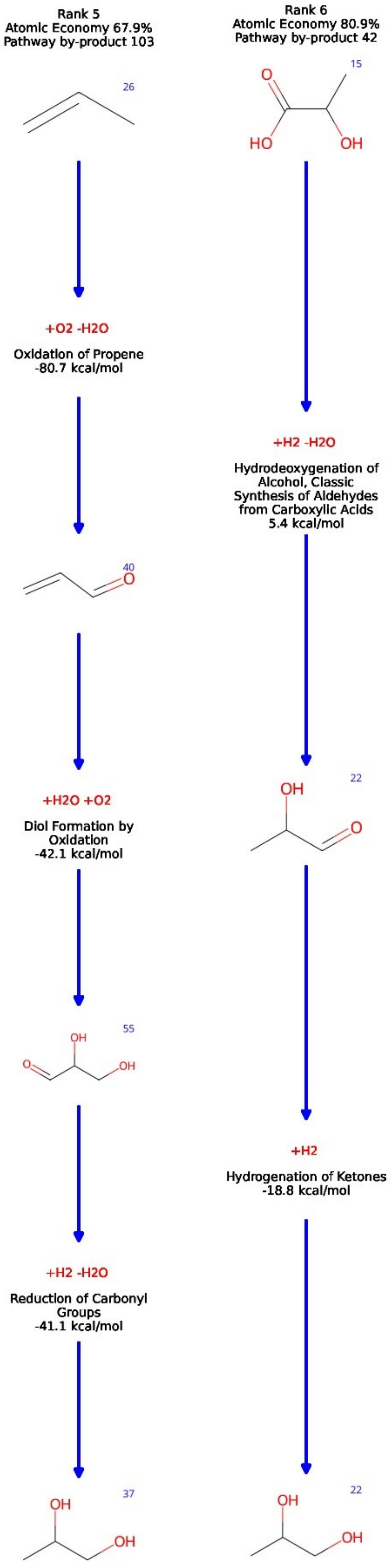

Rank 5
Atomic Economy 67.9%
Pathway by-product 103
26
+O2 -H2O
Oxidation of Propene
-80.7 kcal/mol
40
O
+H2O +O2
Diol Formation by Oxidation
-42.1 kcal/mol
55
OH
O
OH
+H2 -H2O
Reduction of Carbonyl Groups
-41.1 kcal/mol
37
OH
OH
Rank 6
Atomic Economy 80.9%
Pathway by-product 42
15
O
HO
OH
+H2 -H2O
Hydrodeoxygenation of Alcohol, Classic Synthesis of Aldehydes from Carboxylic Acids
5.4 kcal/mol
22
OH
O
+H2
Hydrogenation of Ketones
-18.8 kcal/mol
22
OH
OH

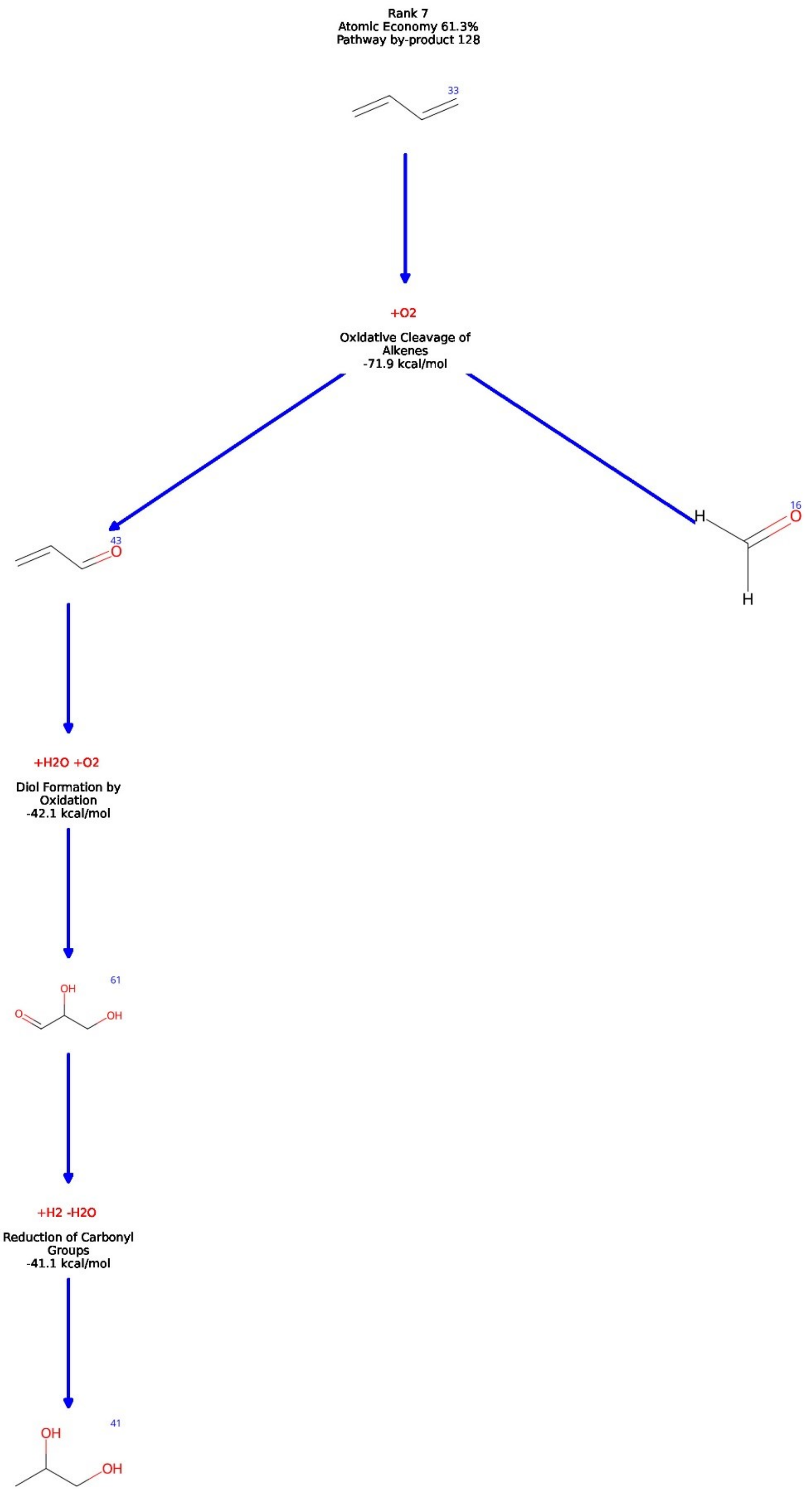
Rank 7
Atomic Economy 61.3%
Pathway by-product 128
33
+O2
Oxidative Cleavage of Alkenes
-71.9 kcal/mol
43
O
16
H
O
H
+H2O +O2
Diol Formation by Oxidation
-42.1 kcal/mol
61
OH
O
OH
+H2 -H2O
Reduction of Carbonyl Groups
-41.1 kcal/mol
41
OH
OH

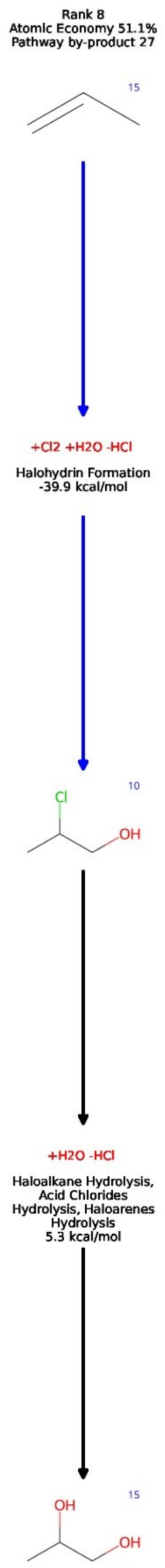
Rank 8
Atomic Economy 51.1%
Pathway by-product 27
15
+Cl2 +H2O -HCl
Halohydrin Formation
-39.9 kcal/mol
10
Cl
OH
+H2O -HCl
Haloalkane Hydrolysis, Acid Chlorides Hydrolysis, Haloarenes Hydrolysis
5.3 kcal/mol
15
OH
OH

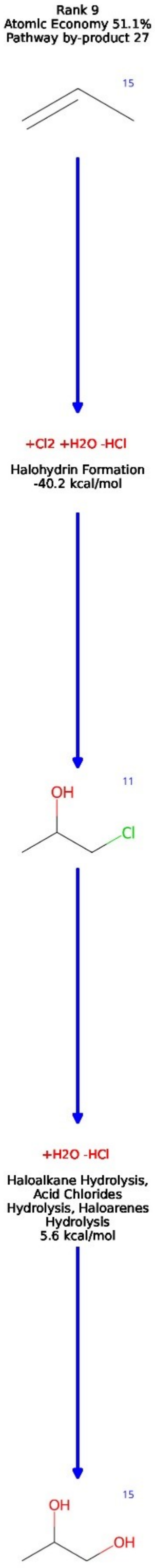
Rank 9
Atomic Economy 51.1%
Pathway by-product 27
15
+Cl2 +H2O -HCl
Halohydrin Formation
-40.2 kcal/mol
OH
Cl
11
+H2O -HCl
Haloalkane Hydrolysis, Acid Chlorides Hydrolysis, Haloarenes Hydrolysis
5.6 kcal/mol
OH
OH
15

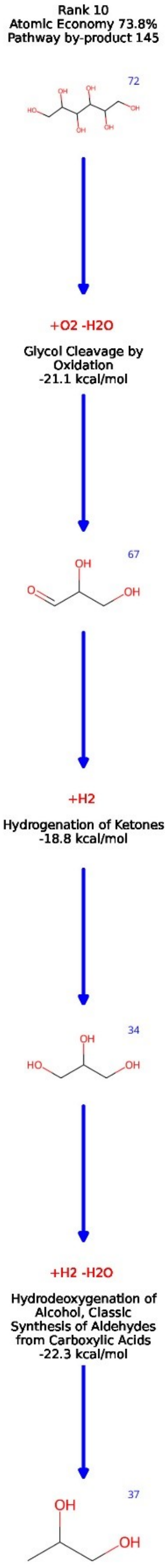
Rank 10
Atomic Economy 73.8%
Pathway by-product 145
72
+O2 -H2O
Glycol Cleavage by Oxidation
-21.1 kcal/mol
OH
O
OH
67
+H2
Hydrogenation of Ketones
-18.8 kcal/mol
OH
HO
OH
34
+H2 -H2O
Hydrodeoxygenation of Alcohol, Classic Synthesis of Aldehydes from Carboxylic Acids
-22.3 kcal/mol
OH
OH
37

Fig. S7. Top 10 ranked pathways to propylene glycol generated by DORAnet, used as part of the baseline result for the sensitivity analysis in Section 4.4. Pathways 1 through 10 are ranked based on the default weights. Each figure is directly output from DORAnet (originally one pathway per page in the output PDF; here, figures are cropped to display multiple pathways per page). Notably, rank 3 corresponds to the current commercial route[1]. Each pathway includes all intermediate molecules and reaction steps. These visualizations are provided to help assess the chemical plausibility of the highest scoring routes discussed in the main text.

**Algorithm 1** Reaction Pathway Search

**Input:** Target molecule $t$, generation limit $G$, maximum pathway length $L$, starter set $S$, minimum atom economy threshold $E$
**Initialize:**
  $pathways\_deque \leftarrow \{[\text{Expandable}, \{(t, 0)\}]\}$ ▷ Each pathway: [status, {(molecule, generation)}, {applied reactions}]
**while** there exists an expandable pathway in $pathways\_deque$ **do**
  Pop an expandable pathway $P$ from the left end of $pathways\_deque$
  Let $(n, g)$ be the first unexpanded molecule in $P$
  **for all** reactions $r$ that produce $n$ with atom economy $\geq E$ **do**
    Create a copy $P'$ of $P$ and append reaction $r$
    Remove $(n, g)$ from $P'$
    **for all** reactants $a$ of reaction $r$ **do**
      **if** adding $a$ violates generation limits **then**
        **Skip** this reaction branch
      **else**
        Add or update reactant $a$ in $P'$ with generation $g + 1$
      **end if**
    **end for**
    **if** $P'$ is viable **then**
      Sort unexpanded molecules in $P'$ by production difficulty
      **if** $P'$ has no remaining molecules to expand **then**
        Mark $P'$ as **complete**
      **else if** the pathway length is within limit **then**
        Mark $P'$ as **expandable** and add it to the left end of $pathways\_deque$
      **end if**
    **end if**
  **end for**
**end while**

Table S1. Computational performance and memory usage for typical runs.

| Job | Generations | Molecules | Reactions | Time (hh:mm:ss) | Memory (Peak, GB) |
|---|---|---|---|---|---|
| Chemocatalytic forward expansion using **alanine** and 6 helpers ($H_2O$, $O_2$, $H_2$, CO, HCl, $Cl_2$), 40 kcal/mol reaction enthalpy threshold | 1 | 18 | 9 | 0:00:03 | 0.22 |
| | 2 | 241 | 366 | 0:00:07 | 0.22 |
| | 3 | 208,919 | 342,700 | 1:29:22 | 2.39 |
| Chemocatalytic forward expansion using **alanine** and 6 helpers ($H_2O$, $O_2$, $H_2$, CO, HCl, $Cl_2$), -10 kcal/mol reaction enthalpy threshold, max product size 6 carbon atoms | 4 | 70 | 90 | 0:00:05 | 0.22 |
| | 5 | 230 | 329 | 0:00:15 | 0.25 |
| | 6 | 1,767 | 2,629 | 0:02:38 | 0.64 |
| Chemocatalytic forward expansion using **alanine** and 6 helpers ($H_2O$, $O_2$, $H_2$, CO, HCl, $Cl_2$), no additional filtering | 1 | 21 | 12 | 0:00:03 | 0.21 |
| | 2 | 573 | 787 | 0:00:11 | 0.22 |
| | 3 | 2,708,079 | 3,949,239 | 21:41:36 | 24.33 |
| Enzymatic forward expansion using **alanine**, no additional filtering | 1 | 78 | 92 | 0:01:05 | 1.44 |
| | 2 | 27,027 | 31,401 | 9:34:02 | 2.70 |
| Chemocatalytic forward expansion using **54 molecules** (feedstock and first-tier targets molecules in the case study), 15 kcal/mol reaction enthalpy threshold, max product size 18 carbon atoms | 1 | 6,405 | 9,038 | 0:02:46 | 0.26 |

| | | | | | |
|---|---|---|---|---|---|
| Chemocatalytic retro expansion using **bisphenol A** and 10 helpers ($H_2O$, $CO_2$, HCl, $Cl_2$, $O_2$, $N_2$, CO, $NH_3$, $H_2S$, trimethylamine), 15 kcal/mol reaction enthalpy threshold, max product size 15 carbon atoms, only homo-bimolecular reactions allowed | 3 | 26,020 | 58,273 | 0:21:02 | 0.96 |
| Chemocatalytic retro expansion using **acetone** and 10 helpers ($H_2O$, $CO_2$, HCl, $Cl_2$, $O_2$, $N_2$, CO, $NH_3$, $H_2S$, trimethylamine), 15 kcal/mol reaction enthalpy threshold, max product size 9 carbon atoms, only homo-bimolecular reactions allowed | 4 | 102,303 | 217,220 | 0:36:20 | 1.55 |
| Chemocatalytic retro expansion using **glycerol** and 10 helpers ($H_2O$, $CO_2$, HCl, $Cl_2$, $O_2$, $N_2$, CO, $NH_3$, $H_2S$, trimethylamine), 15 kcal/mol reaction enthalpy threshold, max product size 10 carbon atoms, only homo-bimolecular reactions allowed | 4 | 871,191 | 2,520,464 | 11:27:21 | 15.97 |
| Enzymatic forward expansion using **glucose**, only homo-bimolecular reactions allowed | 1 | 72 | 91 | 0:00:55 | 1.44 |
| | 2 | 10,224 | 14,224 | 1:45:20 | 1.85 |

| | | | | | |
|---|---|---|---|---|---|
| Enzymatic forward expansion using **phenol**, only homo-bimolecular reactions allowed | 1 | 25 | 30 | 0:00:39 | 1.44 |
| | 2 | 1,046 | 1,468 | 0:10:48 | 1.48 |

Notes:

1. All jobs were executed on a single compute node running Ubuntu 16.04.7 LTS (kernel 4.4.0-210). The node was equipped with an Intel Xeon E5-2640 v4 processor (10 physical cores, 20 threads, 2.40 GHz base frequency), 62 GB of RAM (no swap), and a 2.8 TB NFS-mounted home filesystem. Because DORAnet does not yet support parallel execution, only one core was utilized.
2. To limit the size of the retrosynthetic network, by default only homo-bimolecular reactions (i.e., A + A and A + helper) are permitted in the retro expansions. Hetero-bimolecular (A + B) retro reactions correspond to forward reactions that generate side products and thus compromise atom economy. This filter can be disabled by users. Forward expansions are unaffected by this filter by default.

Table S2. Predefined filters in DORAnet and their purpose.

| Filter Name | Purpose |
|---|---|
| Thermodynamic filter | Rejects reactions with enthalpy or free energy changes exceeding a defined threshold, ensuring that only practically feasible pathways are explored. |
| Molecule size filter | Limits product size based on atom count, excluding overly large molecules that may not be relevant. |
| Multi-reactant filter | Restricts reactions to a single reactant (excluding helper molecules). While this reduces the number of reactions, it sacrifices comprehensiveness. It is useful for tasks where users aim to modify a molecule using only helpers (in chemocatalytic expansion) or cofactors (in enzymatic expansion). |
| Regioselectivity filter | Enforces regioselectivity constraints, including Markovnikov, anti-Markovnikov, Zaitsev, Hofmann (anti-Zaitsev), and Baeyer-Villiger rules. Users can enable one or multiple regioselectivity rules as needed. |
| Co-reactant filter | Rejects reactions with only helper molecules as reactants. |
| Generation filter | Rejects reactions if a reactant's generation number exceeds the user defined limit. |

| | |
|---|---|
| Aromatic filter | Rejects reactions that generate new aromatic rings. |
| Enol filter | Rejects reactions containing an enol reactant unless the reaction is tautomerization. |
| Elements filter | Allows only reactions where reactants are composed of specified elements. |
| Molecule filter | Prevents a specified molecule from being reacted. |

Table S3. Benchmark comparison of DORAnet and other CASP tools

| | DORAnet | Syntheseus[11] | Syntheseus | DORAnet | RetroPath2.0[12] |
|---|---|---|---|---|---|
| Rule set/model | Chemo-catalytic rules | LocalRetroModel | Chemformer Model | Enzymatic (JN3604IMT) | retrorules_rr01_rp2_flat_all[13] |
| Test Dataset | USPTO-50k[14] | USPTO-50k | USPTO-50k | Rhea[15] subset | Rhea subset |
| Reproduction rate | 96.9% | 92.3% | 90.5% | 61.6% | 65.1% |

Notes:

1. Dataset sanitization and preparation:
    a. USPTO-50k: Only carbon-balanced reactions were included, where the reaction centers involve atoms of C, H, O, N, S, or halogens.
    b. Rhea (https://www.rhea-db.org/, retrieved April 2025): Reactions containing incomplete molecules (wildcards) were filtered out. Cofactors were removed, and only monosubstrate reactions were retained, where the substrate contains carbon and has a molecular weight below 1,000 Da. A random subset of 1,000 reactions with unique substrates was selected.
2. Tool settings:
    a. Default settings were used for both Syntheseus and RetroPath2.0.
3. Reproduction criteria:
    a. USPTO-50k: A reaction was considered successfully reproduced if any predicted product set matched the recorded products.
    b. Rhea subset: A reaction was considered successfully reproduced if the recorded product(s) were a subset of any predicted product set, since predicted sets may include cofactors.

Table S4. Case study feedstock molecules.

| Raw Feedstock | Starter molecules for DORAnet |
|---|---|
| Biomass | Syngas ($CO/H_2$) |
| | Glucose |
| | Xylose |
| | Coniferyl alcohol |
| | N-Acetylglucosamine |
| Plastic | Syngas ($CO/H_2$) |
| | Ethylene glycol |
| | Terephthalic acid |
| | Ethylene |
| | Propylene |
| | Xylene |
| | Propane |
| | Benzoic acid |
| | Styrene |
| | HCl |
| Food waste | Glucose |
| | Xylose |
| | Butyric acid |
| | Acetic acid |
| | Valeric acid |
| | Caproic acid |
| | Propionic acid |
| | $CH_4$ and $CO_2$ |
| Fats & oils | Oleic acid |
| | Palmitic acid |
| | Linoleic acid |
| | Stearic acid |
| $CO_2$ | $CO_2$ |
| | CO |
| | $CH_4$ |
| | Ethylene |
| | Formic acid |
| | Methanol |
| | Ethanol |
| $CH_4$ | $CH_4$ |
| Nitrogen | Ammonia |
| | Urea |
| | Nitric acid |
| | NO |
| | $NO_2$ |

| | |
|---|---|
| Chlorine | $Cl_2$ |
| Sulfur | $H_2S$ |

Note: Raw feedstock may require pre-treatment to generate the starter molecules for DORAnet. These included:

1. Mixed sugars from deacetylation and dilute acid hydrolysis of herbaceous biomass
2. Syngas from gasification of woody biomass and plastic waste
3. Volatile fatty acids (e.g., butyric, lactic acid) from arrested anaerobic digestion of food waste
4. Benzoic acid from autooxidation of polystyrene
5. Purified $CO_2$ and $CH_4$ from industrial point sources
6. Formic acid and syngas from $CO_2$ electrolysis

Table S5. Case study target molecules.

| Tier | Name | DORAnet rank number of commercial routes or reason of absence | Number of reactions in the pathway |
|---|---|---|---|
| Tier 1 | Benzene | 14 | 3 |
| | Butadiene | 1 | 3 |
| | Butylene (1-butene) | 1 | 2 |
| | Butylene (2-butene) | 3 | 2 |
| | Butylene (isobutylene) | 4 | 3 |
| | Ethanol | 1 | 1 |
| | Ethylene | 1 | 1 |
| | Glycerol | Commercial routes require triglycerides from oils.[16] | N/A |
| | Lactic acid | 46 | 4 |
| | Lysine | No commercial synthetic route. Produced exclusively via fermentation.[17] | N/A |
| | Methanol | 1 | 1 |
| | Phosgene | 1 | 1 |
| | Propylene | 1 | 1 |
| | Sorbitol | 1 | 1 |
| | Toluene | Commercially extracted from crude petroleum distillates, liquid products from | N/A |

| | | | |
|---|---|---|---|
| | | coal, lignite gasification or coking.[1] | |
| | Xylene (m-) | Commercially obtained from naphtha reformates and hydrocarbons from coal.[1] | N/A |
| | Xylene (o-) | | |
| | Xylene (p-) | | |
| Tier 2 | 2-Ethylhexanol | 15 | 4 |
| | Acetic acid | 2 | 1 |
| | Acrylic acid | 1 | 2 |
| | Acrylonitrile | 1 | 1 |
| | Adiponitrile | 1 | 2 |
| | Butanol (1-) | 4 | 2 |
| | Butanol (2-) | 1 | 1 |
| | Butanol (iso) | 3 | 2 |
| | Butanol (tert butyl alcohol) | 1 | 1 |
| | Cumene | 1 | 1 |
| | Cyclohexane | 1 | 1 |
| | Dinitrotoluene | 1 | 1 |
| | Epichlorohydrin | 14 | 3 |
| | Ethylbenzene | 2 | 1 |
| | Ethylene dichloride | 1 | 1 |
| | Ethylene oxide | 1 | 1 |
| | Formaldehyde | 3 | 1 |
| | Isophthalic acid | 1 | 1 |
| | Isopropanol | 1 | 1 |
| | Maleic anhydride | 1 | 2 |
| | Methionine | 4758 | 8 |
| | Methyl chloride | 1 | 1 |
| | Methyl tert butyl ether | 1 | 1 |
| | Methylamine | 1 | 1 |
| | Methylene chloride | 1 | 2 |
| | Nitrobenzene | 1 | 1 |
| | Phthalic anhydride | 1 | 2 |
| | Propylene oxide | 1 | 1 |
| | Terephthalic acid | 1 | 1 |
| Tier 3 | Butanediol (1,4-) | Commercial (Reppe) process requires acetylene.[1] | N/A |
| | Acetic anhydride | 1 | 1 |
| | Acetone | Commercial (Hock) process requires | N/A |

| | | | |
|---|---|---|---|
| | | phenol as a co-product.[1] | |
| | Aniline | 1 | 2 |
| | Chloroform | 1 | 3 |
| | Cyclohexanol | 1 | 2 |
| | Cyclohexanone | 1 | 2 |
| | Ethylene glycol | 2 | 2 |
| | Hexamethylene diamine | 1 | 4 |
| | Phenol | 1 | 2 |
| | Propylene glycol | 3 | 2 |
| | Styrene | 3 | 2 |
| | Toluene diamine (2,4-Diaminotoluene) | 1 | 2 |
| | Vinyl acetate | 1 | 1 |
| | Vinyl chloride | 3 | 2 |
| Tier 4 | Adipic acid | 3 | 3 |
| | Bisphenol A | 1 | 4 |
| | Caprolactam | 1 | 4 |
| | Methylene diisocyanate | 2 | 5 |
| | Tetrahydrofuran | 2 | 2 |
| | Toluene diisocyanate | 1 | 3 |

Note: Certain targets' commercial routes require feedstocks that are not included in our feedstock list and cannot be derived from available feedstocks; therefore, such routes cannot be reproduced by DORAnet. A special case is acetone, which is commercially co-produced with phenol; this process falls outside the scope of our investigation.

Notably, 27 of the matched pathways involve a single step, reflecting the industrial preference for shorter, more economical processes. 33 pathways are multi-step, particularly among tier 3 and tier 4 targets, which tend to be more complex and require longer synthetic routes. This illustrates DORAnet's capacity to recover both simple and complex pathways effectively.

Table S6. Summary of manually curated chemical/chemocatalytic reaction rules.

| Alkanes | |
|---|---|
| Halogenation of Methane, Halomethanes | 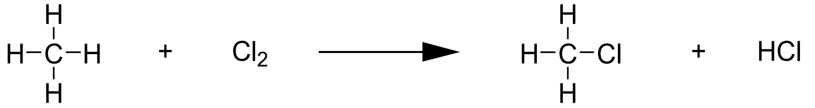 "[C!$(*~[!F!Cl!Br!I])+0!H0:1].[F,Cl,Br,I;+0:2][F,Cl,Br,I;+0:3]>>[*:1][*:2].[*:3]", |

| | |
|---|---|
| Alkane Cracking | 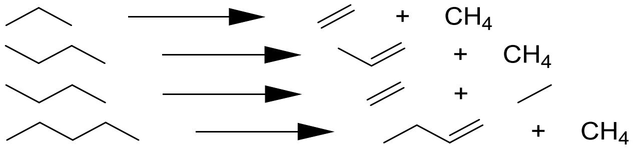<br><br>"[C+0!H0:1][C+0:2]!@[C+0:3]>>[*:1]=[*:2].[*:3]",<br>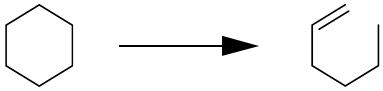<br>"[C+0!H0:1][C+0:2]@[C+0:3]>>([*:1]=[*:2].[*:3])", |
| Alkane Cyclization (A type of Catalytic Reforming) | 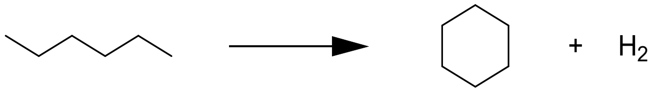<br><br>"[C+0!H0:1][C+0:2][C+0:3][C+0:4][C+0:5][C+0!H0:6]>>[*:1]1[*:2][*:3][*:4][*:5][*:6]1.[H][H]", |
| Alkane Isomerization | 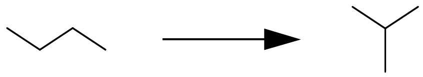<br>"[C+0:1][C+0!H0:2][C+0:3][C+0H3:4]>>[*:1][*:2]([*:3])[*:4]", |
| Alkane Dehydrogenation | 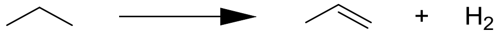<br><br>"[C+0!H0:1][C+0!H0:2]>>[*:1]=[*:2].[H][H]",<br>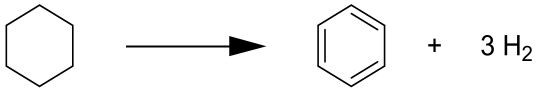<br><br>"([C+0!H0:1][C+0!H0:2].[C+0!H0:3][C+0!H0:4].[C+0!H0:5][C+0!H0:6])>>([*:1]=[*:2].[*:3]=[*:4].[*:5]=[*:6]).[H][H]", |
| **Alkenes** | |
| Hydrogenation of Alkene | 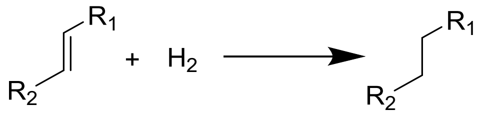<br><br>"[C+0:1]=[C+0:2].[H][H]>>[*:1][*:2]", |
| Oxidative Cleavage of Alkenes | 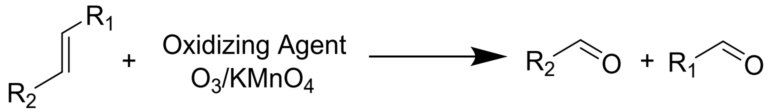<br><br>"[C+0:1]=!@[C+0:2].[O+0:4]=[O+0:5]>>[*:1]=[*:4].[*:2]=[*:5]",<br>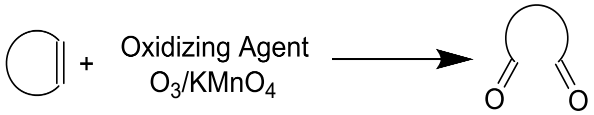<br><br>"[C+0:1]=@[C+0:2].[O+0:4]=[O+0:5]>>([*:1]=[*:4].[*:2]=[*:5])", |
| Olefin Cross Metathesis (CM) | 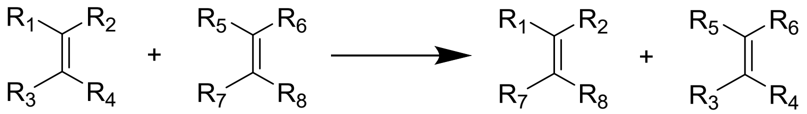<br><br>"[C+0:1]=!@[C+0:2].[C+0:3]=!@[C+0:4]>>[*:1]=[*:3].[*:2]=[*:4]", |

| | |
|---|---|
| Olefin Ring-Opening Metathesis (ROM) | 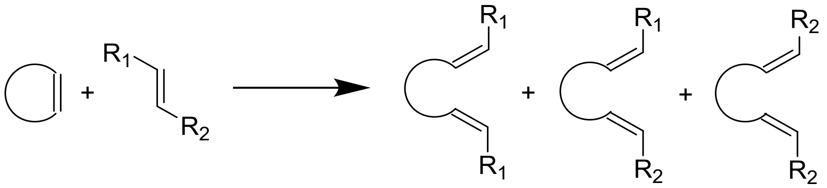<br>"[C+0:1]=@[C+0:2].[C+0:3]=!@[C+0:4]>>([*:1]=[*:3].[*:2]=[*:4])",<br>"[C+0:1]=@[C+0:2].[C+0:3]=!@[C+0:4]>>([*:1]=[*:3].[*:2]=[*:3]).([*:1]=[*:4].[*:2]=[*:4])", |
| Olefin Ring-Closing Metathesis (RCM) | 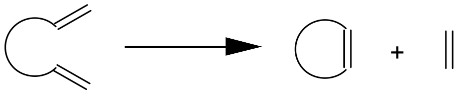<br>"([C+0:1]=!@[C+0:3].[C+0:2]=!@[C+0:4])>>[*:1]=[*:2].[*:3]=[*:4]", |
| Reductive Cross-Coupling of Alkenes | 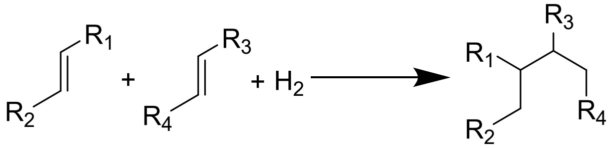<br>"[C+0:1]=[C+0:2].[C+0:3]=[C+0:4].[H][H]>>[*:1][*:2][*:3][*:4]", |
| Epoxidation of Alkene | 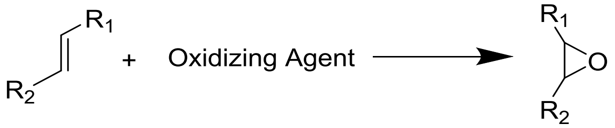<br>"[C+0:1]=[C+0:2].[O+0:3]=[O+0]>>[*:1]1[*:2][*:3]1", |
| Hydration of Alkene, Ethers from Addition of Alcohols or Acids to Alkenes | 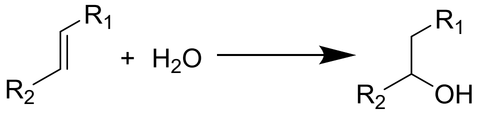<br>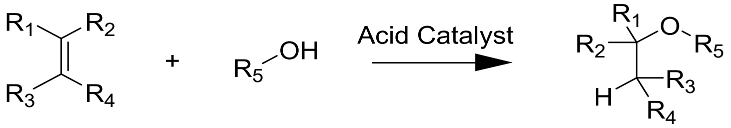<br>"[C+0:1]=[C+0:2].[O+0!H0:3]>>[*:1][*:2][*:3]", Markovnikov<br>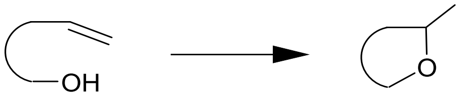<br>"([C+0:1]=[C+0:2].[O+0H:3][C+0:4])>>[*:1][*:2][*:3][*:4]", |
| Hydration of Alkenes, 2-step | 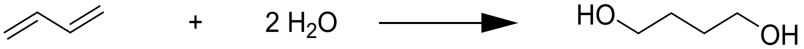<br>"([C+0:1]=[C+0:2].[C+0:3]=[C+0:4]).[O+0H2:5]>>([*:1][*:2][*:5].[*:3][*:4][O])", |
| Hydrohalogenation of Alkenes | 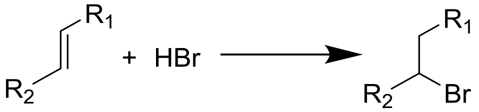<br>"[C+0:1]=[C+0:2].[F,Cl,Br,I;+0H:3]>>[*:1][*:2][*:3]", Markovnikov |
| Halogenation of Alkenes | 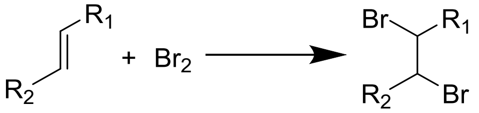<br>"[C+0:1]=[C+0:2].[F,Cl,Br,I;+0:3][F,Cl,Br,I;+0:4]>>[*:1]([*:3])[*:2][*:4]", |

| | |
|---|---|
| Diels-Alder Reaction | 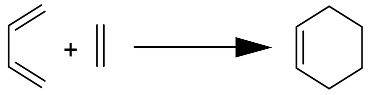<br>"[C+0:1]=[C+0:2][C+0:3]=[C+0:4].[C+0:5]=[C+0:6]>>[*:1]1[*:2]=[*:3][*:4][*:6][*:5]1",<br>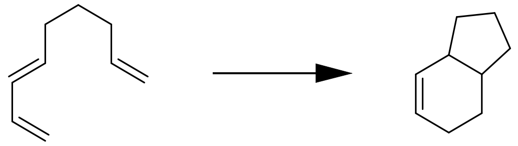<br>"([C+0:1]=[C+0:2][C+0:3]=[C+0:4].[C+0:5]=[C+0:6])>>[*:1]1[*:2]=[*:3][*:4][*:6][*:5]1",<br>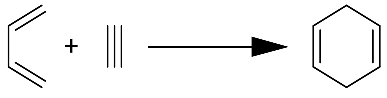<br>"[C+0:1]=[C+0:2][C+0:3]=[C+0:4].[C+0:5]#[C+0:6]>>[*:1]1[*:2]=[*:3][*:4][*:6]=[*:5]1",<br>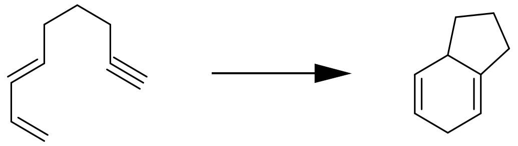<br>"([C+0:1]=[C+0:2][C+0:3]=[C+0:4].[C+0:5]#[C+0:6])>>[*:1]1[*:2]=[*:3][*:4][*:6]=[*:5]1", |
| Diels-Alder Reaction Variations: Oxo-Diels-Alder Reaction, Acrolein Diels-Alder Reaction | 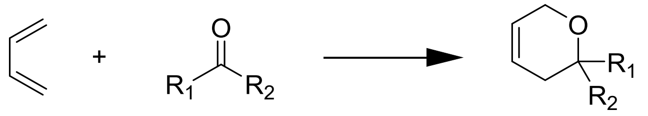<br>"[C+0:1]=[C+0:2][C+0:3]=[C+0:4].[CX3!$(*-O)+0:5]=[O+0:6]>>[*:1]1[*:2]=[*:3][*:4][*:6][*:5]1",<br>"([C+0:1]=[C+0:2][C+0:3]=[C+0:4].[CX3!$(*-O)+0:5]=[O+0:6])>>[*:1]1[*:2]=[*:3][*:4][*:6][*:5]1",<br>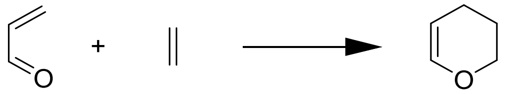<br>"[C+0:1]=[C+0:2][C!$(*-O)+0:3]=[O+0:4].[C+0:5]=[C+0:6]>>[*:1]1[*:2]=[*:3][*:4][*:6][*:5]1",<br>"([C+0:1]=[C+0:2][C!$(*-O)+0:3]=[O+0:4].[C+0:5]=[C+0:6])>>[*:1]1[*:2]=[*:3][*:4][*:6][*:5]1", |
| Halohydrin Formation | 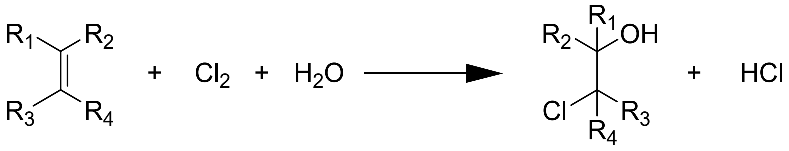<br>"[C+0:1]=[C+0:2].[F,Cl,Br,I;+0:3][F,Cl,Br,I;+0:4].[O+0H2:5]>>[*:1]([*:5])[*:2][*:3].[*:4]", |
| Diol Formation by Oxidation | 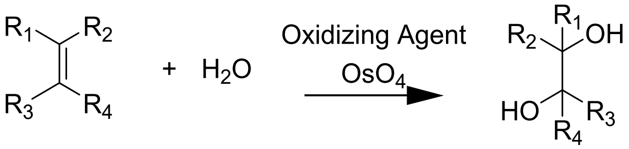<br>"[C+0:1]=[C+0:2].[O+0:3]=[O+0].[O+0H2:4]>>[*:1]([*:3])[*:2][*:4]", |
| Conjugated Dienes 1,4 Addition | 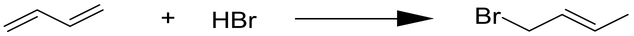<br>"[C+0:1]=[C+0:2][C+0:3]=[C+0:4].[F,Cl,Br,I;+0H:5]>>[*:5][*:1][*:2]=[*:3][*:4]",<br>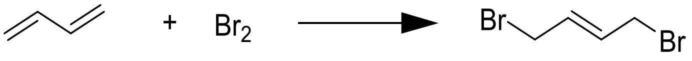<br>"[C+0:1]=[C+0:2][C+0:3]=[C+0:4].[F,Cl,Br,I;+0:5][F,Cl,Br,I;+0:6]>>[*:5][*:1][*:2]=[*:3][*:4][*:6]", |

| | |
|---|---|
| Claisen Rearrangement, Cope Rearrangements | 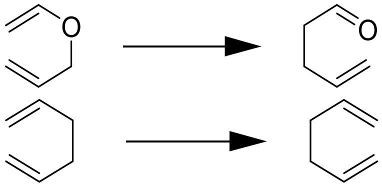<br>"[C+0:1]=[C+0:2][O,C;+0:3][C+0:4][C+0:5]=[C+0:6]>>[*:3]=[*:2][*:1][*:6][*:5]=[*:4]", |
| Aromatic Claisen Rearrangement | 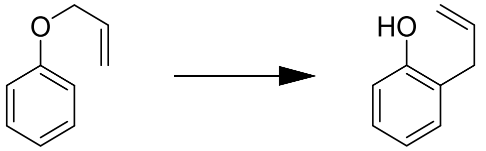<br>"[c+0H:1]1:[c+0:2]:[c+0:3]:[c+0:4]:[c+0:5]:[c+0:6]:1-[O+0:7][C+0:8][C+0:9]=[C+0:10]>>[*:1]1([*:10][*:9]=[*:8]):[*:2]:[*:3]:[*:4]:[*:5]:[*:6]:1-[*:7]",<br>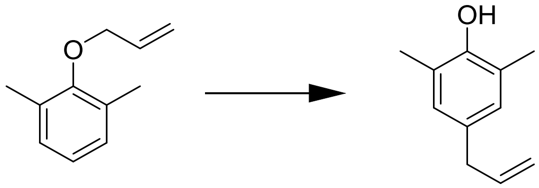<br>"[c+0H0:1]1:[c+0:2]:[c+0:3]:[c+0:4]:[c+0H0:5]:[c+0:6]:1-[O+0:7][C+0:8][C+0:9]=[C+0:10]>>[*:1]1:[*:2]:[*:3]([*:8][*:9]=[*:10]):[*:4]:[*:5]:[*:6]:1-[*:7]", |
| Tsuji–Trost Reaction | 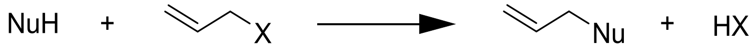<br>Nu = $ECH_2E$, enolates, amines, phenols etc. X = Cl, OC(=O)R etc.<br>"[C+0:1]=[C+0:2][C+0;!$(*[OH]);!$(*=O):3]-!@[F,Cl,Br,I,O$(*C=O);+0:4].[C+0:5](=[O+0:6])[C+0!H0:7][C+0:8]=[O+0:9]>>[*:1]=[*:2][*:3][*:7]([*:5]=[*:6])[*:8]=[*:9].[*:4]", "[C+0:1]=[C+0:2][C+0;!$(*[OH]);!$(*=O):3]-!@[F,Cl,Br,I,O$(*C=O);+0:4].[C+0:5][C+0!H0:7][C+0;!$(*CC=O):8]=[O+0:9]>>[*:1]=[*:2][*:3][*:7]([*:5])[*:8]=[*:9].[*:4]", "[C+0:1]=[C+0:2][C+0;!$(*[OH]);!$(*=O):3]-!@[F,Cl,Br,I,O$(*C=O);+0:4].[N+0!H0:7][C,c;+0:8]>>[*:1]=[*:2][*:3][*:7][*:8].[*:4]",<br>"[C+0:1]=[C+0:2][C+0;!$(*[OH]);!$(*=O):3]-!@[F,Cl,Br,I,O$(*C=O);+0:4].[O+0H:7][c+0:8]>>[*:1]=[*:2][*:3][*:7][*:8].[*:4]",<br>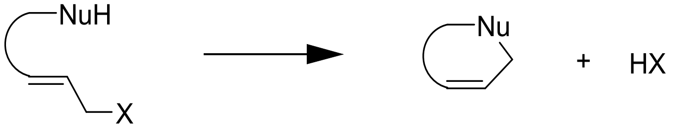<br>"([C+0:1]=[C+0:2][C+0;!$(*[OH]);!$(*=O):3]-!@[F,Cl,Br,I,O$(*C=O);+0:4].[C+0:5](=[O+0:6])[C+0!H0:7][C+0:8]=[O+0:9])>>[*:1]=[*:2][*:3][*:7]([*:5]=[*:6])[*:8]=[*:9].[*:4]",<br>"([C+0:1]=[C+0:2][C+0;!$(*[OH]);!$(*=O):3]-!@[F,Cl,Br,I,O$(*C=O);+0:4].[C+0:5][C+0!H0:7][C+0;!$(*CC=O):8]=[O+0:9])>>[*:1]=[*:2][*:3][*:7]([*:5])[*:8]=[*:9].[*:4]",<br>"([C+0:1]=[C+0:2][C+0;!$(*[OH]);!$(*=O):3]-!@[F,Cl,Br,I,O$(*C=O);+0:4].[N+0!H0:7][C,c;+0:8])>>[*:1]=[*:2][*:3][*:7][*:8].[*:4]",<br>"([C+0:1]=[C+0:2][C+0;!$(*[OH]);!$(*=O):3]-!@[F,Cl,Br,I,O$(*C=O);+0:4].[O+0H:7][c+0:8])>>[*:1]=[*:2][*:3][*:7][*:8].[*:4]", |
| Oxychlorination | 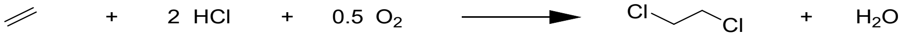<br>"[C+0:1]=[C+0:2].[F,Cl,Br,I;+0H:3].[O+0:4]=[O+0]>>[*:1]([*:3])[*:2][*:3].[*:4]", |

| | |
|---|---|
| Acetoxylation of Alkenes | 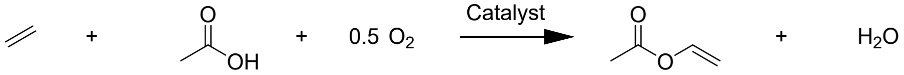<br>"[C+0!H0:1]=[C+0:2].[C+0:3](=[O+0:4])[O+0H:5].[O+0:6]=[O+0]>>[*:3](=[*:4])[*:5][*:1]=[*:2].[*:6]",<br>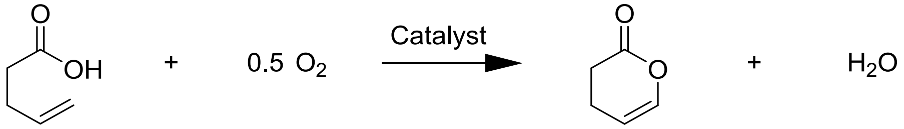<br>"([C+0!H0:1]=[C+0:2].[C+0:3](=[O+0:4])[O+0H:5]).[O+0:6]=[O+0]>>[*:3](=[*:4])[*:5][*:1]=[*:2].[*:6]"<br>, |
| Oxidation of Propene | 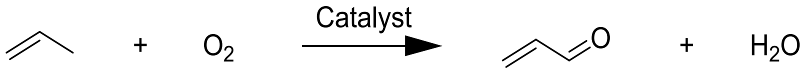<br>"[C+0H2:1]=[C+0H:2][C+0H3:3].[O+0:4]=[O+0:5]>>[*:1]=[*:2][*:3]=[*:4].[*:5]", |
| **Alkynes** | |
| Halogen Addition of Alkynes | 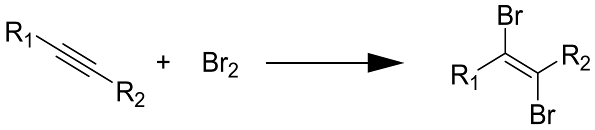<br>"[C+0:1]#[C+0:2].[F,Cl,Br,I;+0:3][F,Cl,Br,I;+0:4]>>[*:1]([*:3])=[*:2][*:4]", |
| Hydrogen Halides Addition of Alkynes | 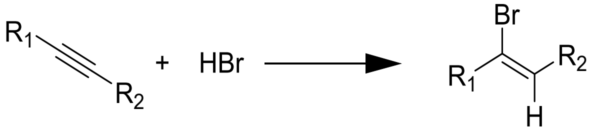<br>"[C+0:1]#[C+0:2].[F,Cl,Br,I;+0H:3]>>[*:1]([*:3])=[*:2]", Markovnikov |
| Hydration of Alkynes | 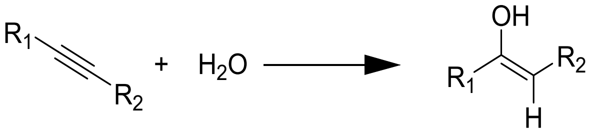<br>"[C+0:1]#[C+0:2].[O+0H2:3]>>[*:1]([*:3])=[*:2]", Markovnikov |
| Hydrogenation of Alkynes | 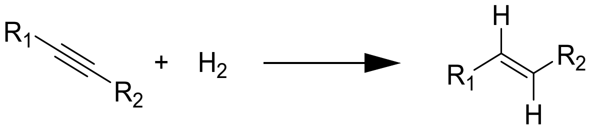<br>"[C+0:1]#[C+0:2].[H][H]>>[*:1]=[*:2]",<br>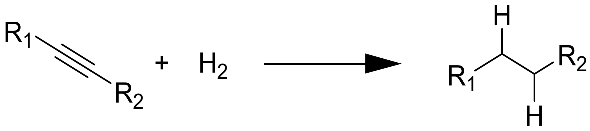<br>"[C+0:1]#[C+0:2].[H][H]>>[*:1][*:2]", |
| Gentle Alkyne Oxidation | 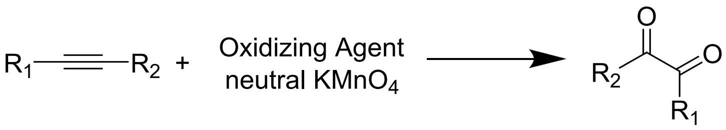<br>"[C+0:1]#[C+0:2].[O+0:4]=[O+0:5]>>[*:1](=[*:4])[*:2]=[*:5]", |

<table>
<tr><td>Oxidative Cleavage of Alkynes</td><td>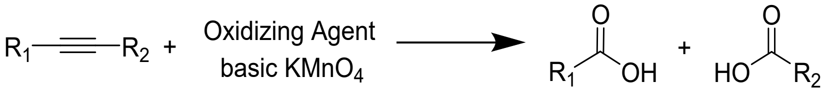

"[*:6][C+0:1]#!@[C+0:2][*:7].[O+0H2:3].[O+0:4]=[O+0:5]>>[*:6][*:1](=[*:3])[*:4].[*:7][*:2](=[*:5])[O]"<br>,<br>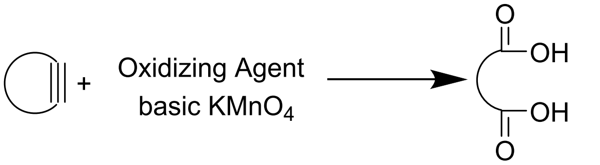

"[*:6][C+0:1]#@[C+0:2][*:7].[O+0H2:3].[O+0:4]=[O+0:5]>>([*:6][*:1](=[*:3])[*:4].[*:7][*:2](=[*:5])[O])<br>",<br>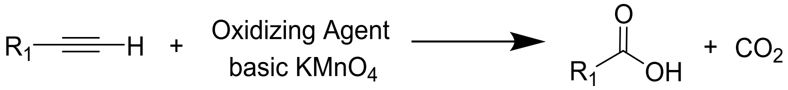

"[*:6][C+0:1]#[C+0H:2].[O+0:4]=[O+0:5]>>[*:6][*:1](=[*:4])[*:5].[O]=[*:2]=[O]",</td></tr>
<tr><td>Alkynes by Dehydrohalogenation</td><td>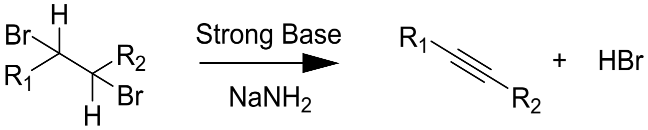

"[C+0!H0:1]([F,Cl,Br,I;+0:2])[C+0!H0:3]([F,Cl,Br,I;+0])>>[*:1]#[*:3].[*:2]",<br>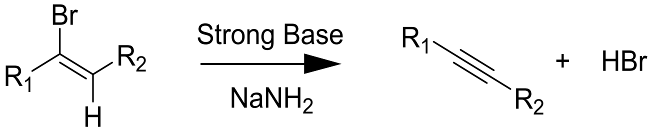

"[C+0:1]([F,Cl,Br,I;+0:2])=[C+0!H0:3]>>[*:1]#[*:3].[*:2]",</td></tr>
<tr><td>Alkylation of Acetylide Anions with Methyl and Primary Haloalkanes</td><td>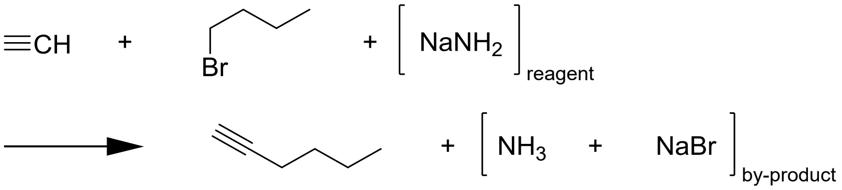

"[C+0:1]#[C+0H:2].[F,Cl,Br,I;+0:3][C;H2,H3;+0:4]>>[*:1]#[*:2][*:4].[*:3]",</td></tr>
<tr><td>Sonogashira Coupling, Acyl Sonogashira Coupling</td><td>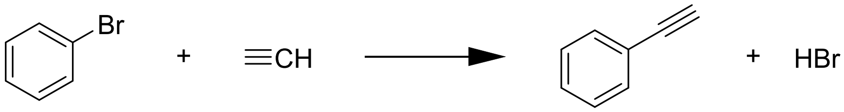<br>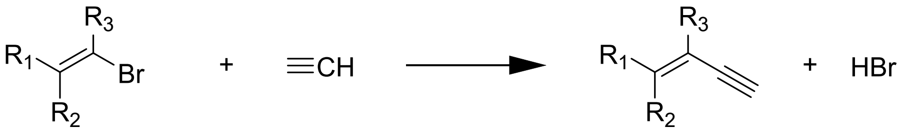<br>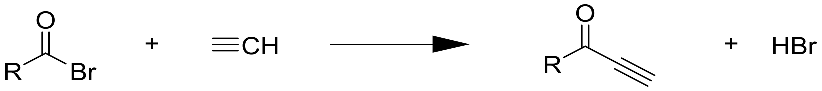
"[C+0:1]#[C+0H:2].[F,Cl,Br,I;+0:3][c,C$(*=C),C$(*=O);+0:4]>>[*:1]#[*:2][*:4].[*:3]",</td></tr>
</table>

| | |
|---|---|
| Addition of Alkynes to Aldehydes and Activated Ketones (Reppe Synthesis) | 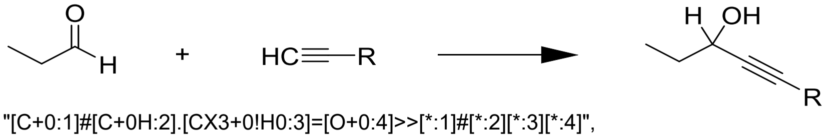<br>"[C+0:1]#[C+0H:2].[CX3+0!H0:3]=[O+0:4]>>[*:1]#[*:2][*:3][*:4]",<br>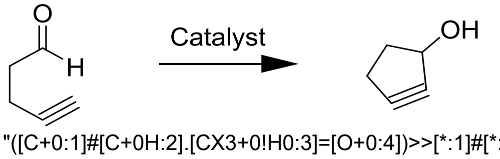<br>"([C+0:1]#[C+0H:2].[CX3+0!H0:3]=[O+0:4])>>[*:1]#[*:2][*:3][*:4]",<br>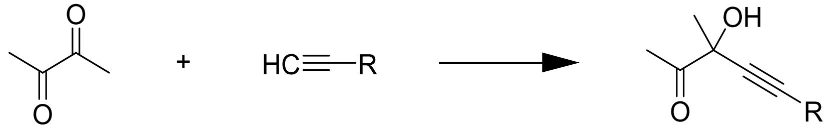<br>"[C+0:1]#[C+0H:2].[CX3$(*=O)+0:5][CX3!$(*O)+0:3]=[O+0:4]>>[*:1]#[*:2][*:3]([*:4])[*:5]", |
| **Haloalkanes, Halogenation** | |
| Alcohols React with Hydrogen Halides to form Haloalkanes, Carboxylic Acids Conversion to Acid Chlorides | $R{-}OH + H{-}X \longrightarrow R{-}X + H_2O$<br>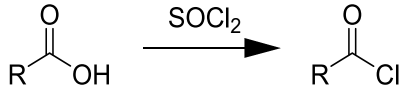<br>"[C+0:1][O+0H:2].[F,Cl,Br,I;+0H:3]>>[*:1][*:3].[*:2]", |
| Haloalkane Hydrolysis, Acid Chlorides Hydrolysis, Haloarenes Hydrolysis | $R{-}X + H_2O \longrightarrow R{-}OH + H{-}X$<br>"[C,c;+0:1][F,Cl,Br,I;+0:2].[O+0H2:3]>>[*:1][*:3].[*:2]", |
| Haloarenes Hydrolysis, Displacement | 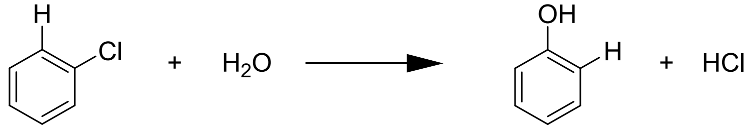<br>"[c;+0H:1][c;+0:2][F,Cl,Br,I;+0:3].[O+0H2:4]>>[*:4][*:1][*:2].[*:3]", |
| Wurtz Reaction, Coupling of Halides with Gilman Reagent<br><br>(Corey–House synthesis) | $2\ R{-}X + 2\ Na \longrightarrow R{-}R + 2\ NaX$<br>$2\ R_1{-}I \xrightarrow[\text{CuI}]{\text{Li, pentane}} (R_1)_2CuLi + R_2{-}Br \xrightarrow[\text{or THF}]{\text{Diethyl ether}} R_1{-}R_2$<br>"[C,c;+0:1][F,Cl,Br,I;+0:2].[C,c;+0:3][F,Cl,Br,I;+0:4]>>[*:1][*:3].[*:2][*:4]",<br>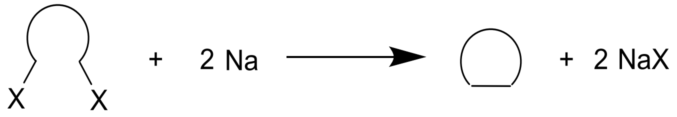<br>"([C,c;+0:1][F,Cl,Br,I;+0:2].[C,c;+0:3][F,Cl,Br,I;+0:4])>>[*:1][*:3].[*:2][*:4]", |

| | |
|---|---|
| Dehydrohalogenati<br>on | 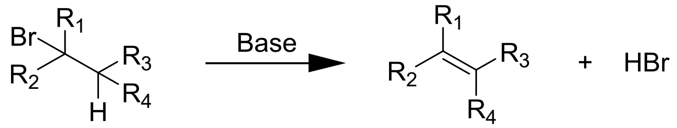<br>"[C+0:1]([F,Cl,Br,I;+0:2])[C+0!H0:3]>>[*:1]=[*:3].[*:2]", Zaitsev |
| Aromatic<br>Halogenation | 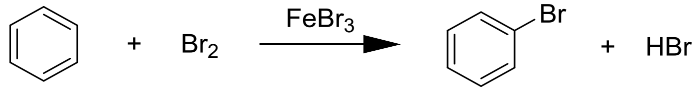<br>"[c+0H:1].[F,Cl,Br,I;+0:2][F,Cl,Br,I;+0:3]>>[*:1][*:2].[*:3]", |
| Friedel–Crafts<br>Reaction | 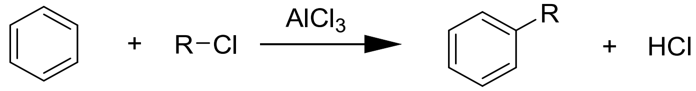<br>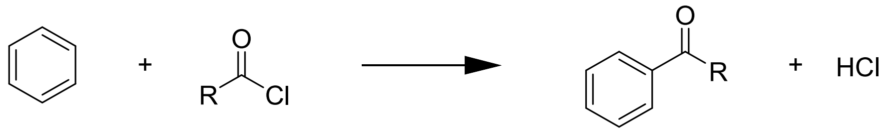<br>"[c+0H:1].[CX4,C$(*=O);+0:2][F,Cl,Br,I;+0:3]>>[*:1][*:2].[*:3]",<br>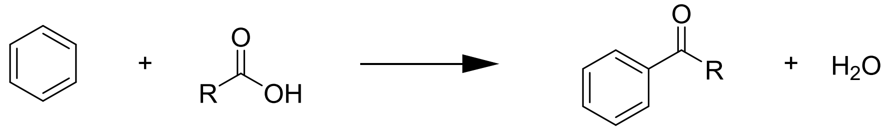<br>"[c+0H:1].[C$(*=O)+0:2][O+0H:3]>>[*:1][*:2].[*:3]",<br>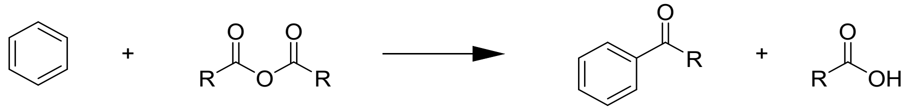<br>"[c+0H:1].[C$(*=O)+0:2][O+0:3]!@[C$(*=O)+0:4]>>[*:1][*:2].[*:3][*:4]",<br>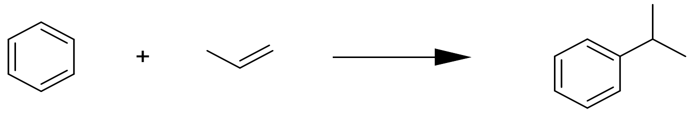<br>"[c+0H:1].[C+0:2]=[C+0:3]>>[*:1][*:2][*:3]",<br>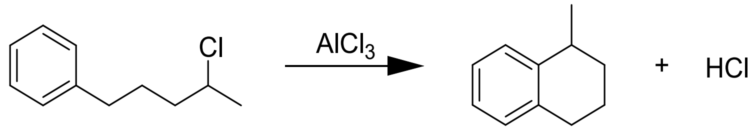<br>"([c+0H:1].[CX4,C$(*=O);+0:2][F,Cl,Br,I;+0:3])>>[*:1][*:2].[*:3]",<br>"([c+0H:1].[C$(*=O)+0:2][O+0H:3])>>[*:1][*:2].[*:3]",<br>"([c+0H:1].[C+0:2]=[C+0:3])>>[*:1][*:2][*:3]", |

| | |
|---|---|
| Heck Reaction | 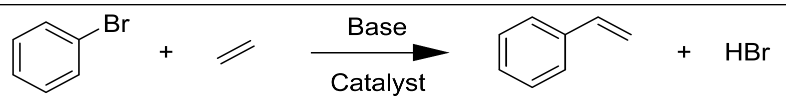<br>"[C+0:1]=[C+0!H0:2].[c+0:3][F,Cl,Br,I;+0:4]>>[*:1]=[*:2][*:3].[*:4]",<br>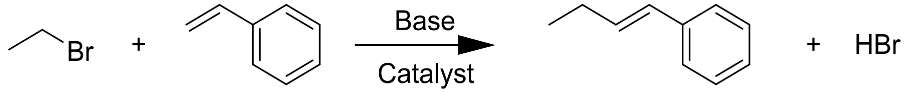<br>"[c+0:5][C+0:1]=[C+0!H0:2].[C,c;+0:3][F,Cl,Br,I;+0:4]>>[*:5][*:1]=[*:2][*:3].[*:4]",<br>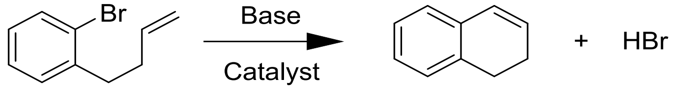<br>"([C+0:1]=[C+0!H0:2].[c+0:3][F,Cl,Br,I;+0:4])>>[*:1]=[*:2][*:3].[*:4]",<br>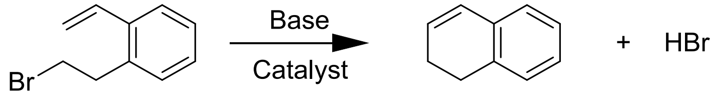<br>"([c+0:5][C+0:1]=[C+0!H0:2].[C,c;+0:3][F,Cl,Br,I;+0:4])>>[*:5][*:1]=[*:2][*:3].[*:4]", |
| Suzuki Coupling (Combined with Grignard Reagent and Borate Ester Formation, or Hydroboration of Alkenes or Alkynes) | 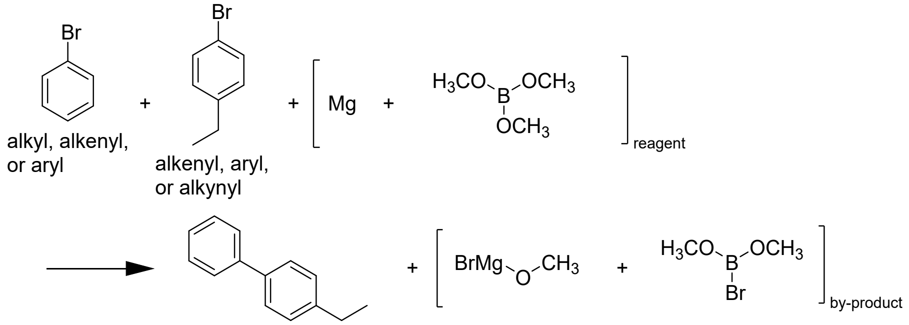<br>"[c,C!$(*~O);!$(*#*);+0:1][F,Cl,Br,I;+0].[c,C$(*=,#C);+0:2][F,Cl,Br,I;+0]>>[*:1][*:2]",<br>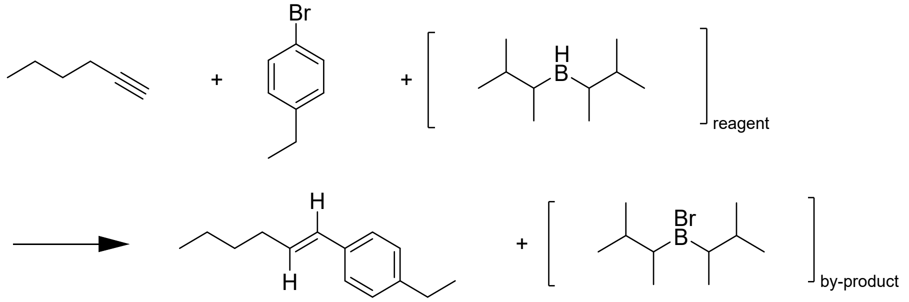<br>"[C+0:1]=[C+0:2].[c,C$(*=,#C);+0:3][F,Cl,Br,I;+0]>>[*:1]-[*:2][*:3]",<br>"[C+0:1]#[C+0:2].[c,C$(*=,#C);+0:3][F,Cl,Br,I;+0]>>[*:1]=[*:2][*:3]", |
| Reduction of Haloalkanes | 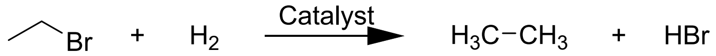<br>"[CX4,c;+0:1][F,Cl,Br,I;+0:2].[H][H]>>[*:1].[*:2]", |
| Grignard Reagent with Oxiranes (Combined with Grignard Reagent Formation) | 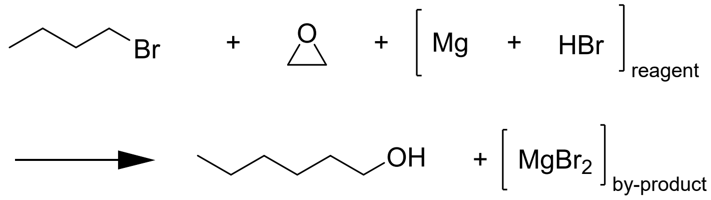<br>"[c,C!$(*~O);!$(*#*);+0:1][F,Cl,Br,I;+0].[C+0:2]1[C+0:3][O+0:4]1>>[*:1][*:2][*:3][*:4]", |

| | |
|---|---|
| Dichlorocarbene with Alkenes | 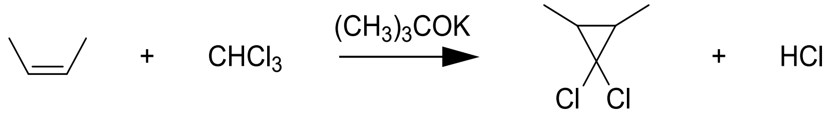<br>"[C+0H:1]([F,Cl,Br,I;+0:2])([F,Cl,Br,I;+0:3])[F,Cl,Br,I;+0:4].[C+0:5]=[C+0:6]>>[*:1]1([*:2])([*:3])[*:5][*:6]1.[*:4]", |
| Simmons-Smith Reaction | 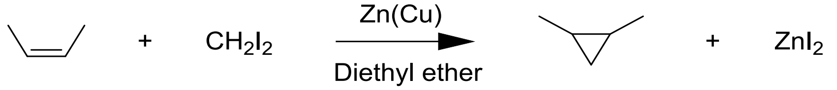<br>"[C+0H2:1]([F,Cl,Br,I;+0:2])[F,Cl,Br,I;+0:3].[C+0:5]=[C+0:6]>>[*:1]1[*:5][*:6]1.[*:2].[*:3]", |
| Esterification with Alkyl and Aryl Halides | 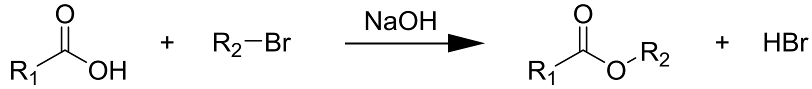<br>"[C+0:2](=[O+0:3])[O+0H:4].[CX4,c;+0:5][F,Cl,Br,I;+0:6]>>[*:2](=[*:3])[*:4][*:5].[*:6]",<br>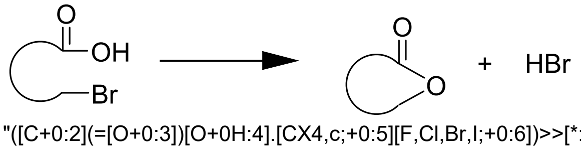<br>"([C+0:2](=[O+0:3])[O+0H:4].[CX4,c;+0:5][F,Cl,Br,I;+0:6])>>[*:2](=[*:3])[*:4][*:5].[*:6]", |
| Allylic, Benzylic Halogenation (Wohl–Ziegler Reaction) | 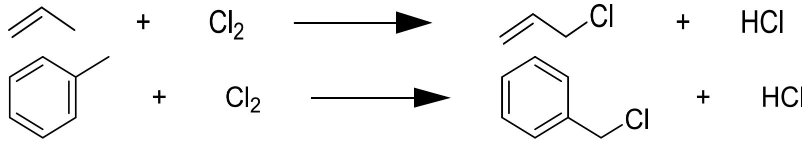<br>"[C$(*=C),c;+0:1][CX4!$(*~[O,N,S])+0!H0:2].[F,Cl,Br,I;+0:3][F,Cl,Br,I;+0:4]>>[*:1][*:2][*:3].[*:4]", |
| Phosgene Production | 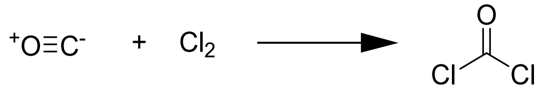<br>"[C-]#[O+].[F,Cl,Br,I;+0:3][F,Cl,Br,I;+0:4]>>[*:3][C](=[O])[*:4]", |
| Enolate Alkylation | 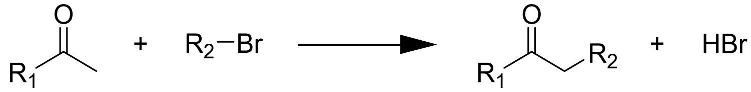<br>"[C+0:2](=[O+0:3])[C+0!H0:4].[CX4;+0:5][F,Cl,Br,I;+0:6]>>[*:2](=[*:3])[*:4][*:5].[*:6]",<br>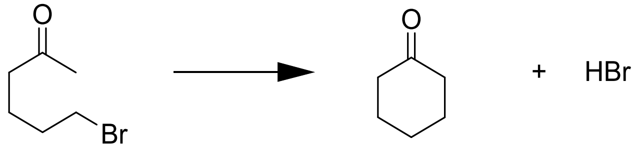<br>"([C+0:2](=[O+0:3])[C+0!H0:4].[CX4;+0:5][F,Cl,Br,I;+0:6])>>[*:2](=[*:3])[*:4][*:5].[*:6]", |
| **Alcohols** | |
| Glycol Cleavage by Oxidation | 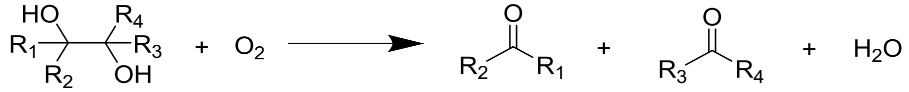<br>"[C+0:1]([O+0H:5])-!@[C+0:2][O+0H:6].[O+0:3]=[O+0]>>[*:1]=[*:5].[*:2]=[*:6].[*:3]",<br>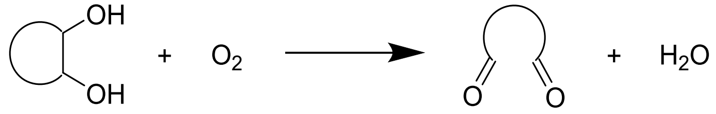<br>"[C+0:1]([O+0H:5])-@[C+0:2][O+0H:6].[O+0:3]=[O+0]>>([*:1]=[*:5].[*:2]=[*:6]).[*:3]", |

| | |
|---|---|
| Hydrodeoxygenation of Alcohol, Classic Synthesis of Aldehydes from Carboxylic Acids | 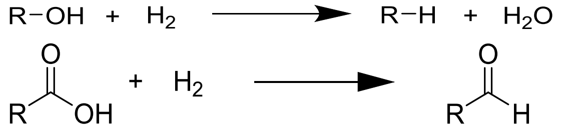<br>"[C,c;+0:1][O+0H:2].[H][H]>>[*:1].[*:2]", |
| Dehydration of Alcohols | 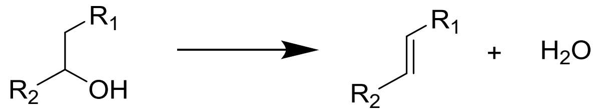<br>"[C+0!H0:1][C+0:2][O+0H:3]>>[*:1]=[*:2].[*:3]", Zaitsev |
| Dehydration of Alcohols, 2-step | 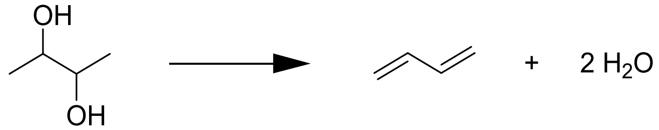<br>"([C+0!H0:1][C+0:2][O+0H:3].[C+0!H0:4][C+0:5][O+0H])>>([*:1]=[*:2].[*:4]=[*:5]).[*:3]", |
| Selective Oxidation of Alcohols | 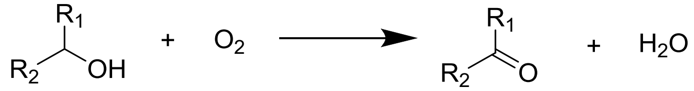<br>"[C+0!H0:1][O+0H:2].[O+0:3]=[O+0]>>[*:1]=[*:2].[*:3]", |
| Diol Carboxylation | 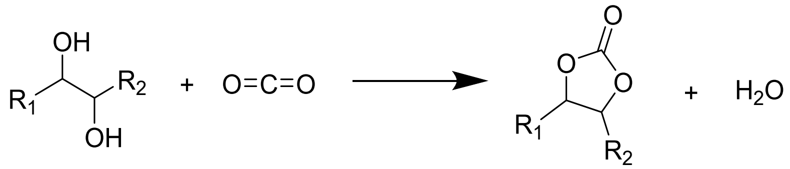<br>"[O+0H:1][C+0:2][C+0:3][O+0H:4].[O+0:5]=[C+0:6]=[O+0:7]>>[*:1].[*:2]1[*:3][*:4][*:6](=[*:7])[*:5]1"<br>,<br>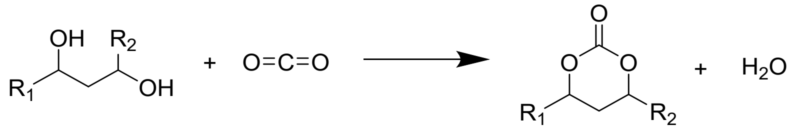<br>"[O+0H:1][C+0:2][C+0:8][C+0:3][O+0H:4].[O+0:5]=[C+0:6]=[O+0:7]>>[*:1].[*:2]1[*:8][*:3][*:4][*:6](<br>=[*:7])[*:5]1", |
| Hydrogenolysis of Primary Alcohol | 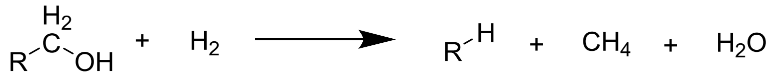<br>"[C+0:1][C+0H2:3][O+0H:2].[H][H]>>[*:1].[*:3].[*:2]", |
| Pinacol Rearrangement | 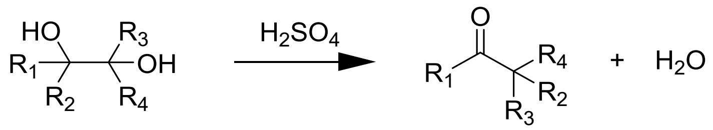<br>"[C+0:1][C+0:2]([C+0:3])([O+0H:4])[C+0X4:5][O+0H:8]>>[*:1][*:2](=[*:4])[*:5]([*:3]).[*:8]",<br>"[C+0H:2]([C+0:3])([O+0H:4])[C+0X4:5][O+0H:8]>>[*:2](=[*:4])([*:3])[*:5].[*:8]",<br>"[C+0H2:2]([O+0H:4])[C+0X4:5][O+0H:8]>>[*:2](=[*:4])[*:5].[*:8]", |
| Dehydration of Geminal Diol | 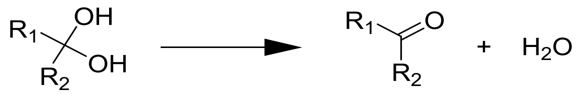<br>"[C+0:1]([O+0H:2])[O+0H:3]>>[*:1]=[*:2].[*:3]", |

<table>
<tr><td>Formation of Acetals from Hemiacetals</td><td>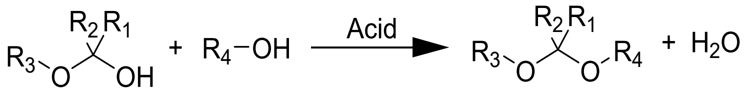

"[C+0X4:1]([O+0H0X2:2])[O+0H:3].[O+0H:4][C+0X4;!$(*OC):5]>>[*:1]([*:2])[*:4][*:5].[*:3]",</td></tr>
<tr><td>Oxidation Of Primary Alcohols to Carboxylic Acids</td><td>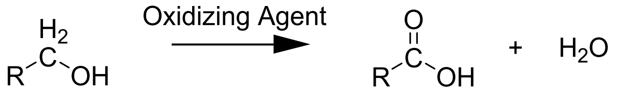

"[C+0;H3,H2:1][O+0H:2].[O+0:3]=[O+0:4]>>[*:1](=[*:3])[*:2].[*:4]",</td></tr>
<tr><td>Oxidation Of Primary Alcohols to Carboxylic Acids, 2-step</td><td>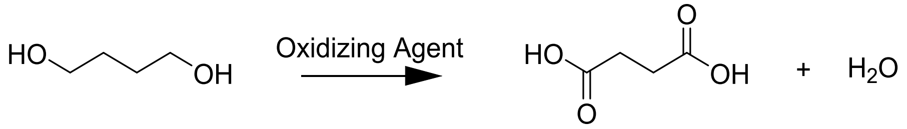

"([C+0;H3,H2:1][O+0H:2].[C+0;H3,H2:5][O+0H:6]).[O+0:3]=[O+0:4]>>([*:1](=[*:3])[*:2].[*:5](=[*:6])[O]).[*:4]",</td></tr>
<tr><td>Formation of Cyclic Acetals from Ketones/Aldehydes with Diols</td><td>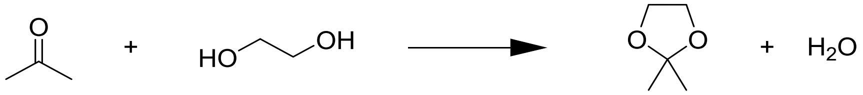
"[C+0X3!$(*O):1]=[O+0:2].[O+0H:3][C+0X4:4][C+0X4:5][O+0H:6]>>[*:1]1[*:3][*:4][*:5][*:6]1.[*:2]",</td></tr>
<tr><td>Oxidation of Cyclohexane</td><td>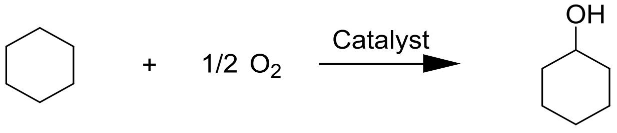

"[C+0H2:1]1[C+0H2:2][C+0H2:3][C+0H2:4][C+0H2:5][C+0H2:6]1.[O+0:7]=[O+0]>>[*:1]1[*:2][*:3][*:4][*:5][*:6]1[*:7]",
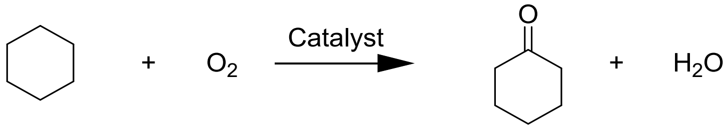

"[C+0H2:1]1[C+0H2:2][C+0H2:3][C+0H2:4][C+0H2:5][C+0H2:6]1.[O+0:7]=[O+0:8]>>[*:1]1[*:2][*:3][*:4][*:5][*:6]1=[*:7].[*:8]",</td></tr>
<tr><td>Methanol from Syngas</td><td>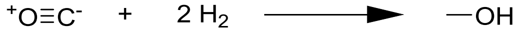
"[C-]#[O+].[H][H]>>[C][O]",</td></tr>
<tr><td colspan="2">

# Ethers, Epoxides

</td></tr>
<tr><td>Hydrolysis of Ethers, Esters, Anhydrides</td><td>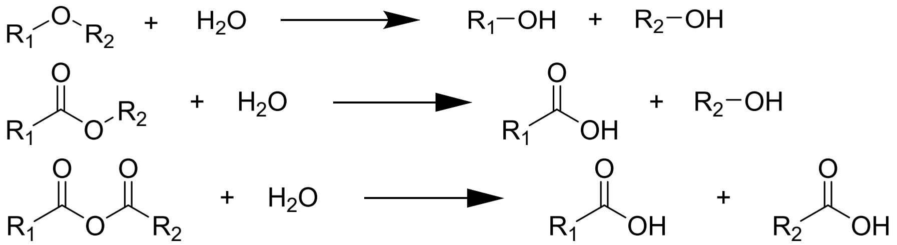
"[C,c;+0:1][O+0:2]-!@[C,c;+0:3].[O+0H2:4]>>[*:1][*:2].[*:3][*:4]",
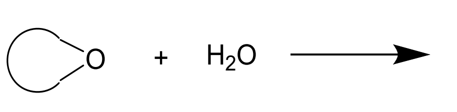</td></tr>
</table>

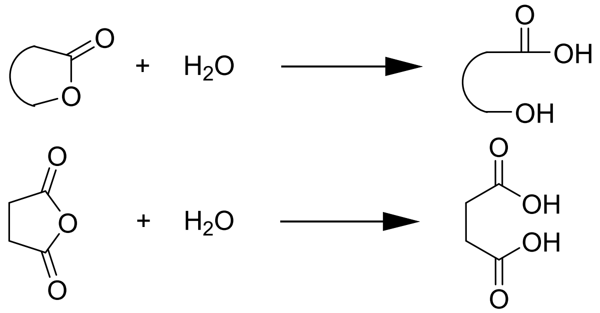

"[C,c;+0:1][O+0:2]-@[C,c;+0:3].[O+0H2:4]>>([*:1][*:2].[*:3][*:4])",

**Hydrogenolysis of Ethers**

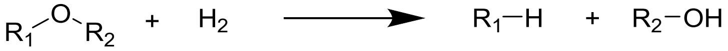

"[C,c;+0;!$(*=O):1]!@[O+0:2][C,c;+0;!$(*=O):3].[H][H]>>[*:1].[*:2][*:3]",

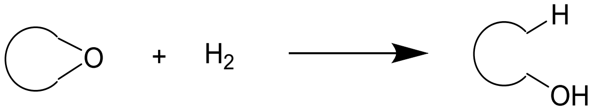

"[C,c;+0;!$(*=O):1]@[O+0:2][C,c;+0;!$(*=O):3].[H][H]>>([*:1].[*:2][*:3])",

**Williamson Ether Synthesis, Ullmann Condensation**

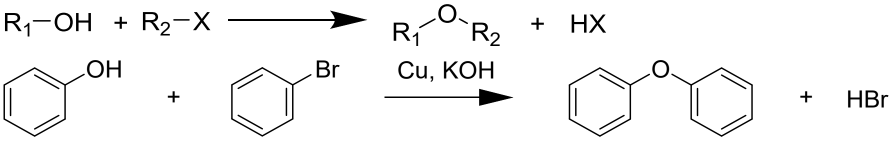

"[C,c;!$(*=O);+0:1][O+0H:2].[CX4!H0,c;+0:3][F,Cl,Br,I;+0:4]>>[*:1][*:2][*:3].[*:4]",

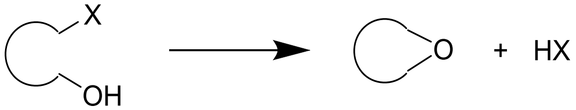

"([C,c;!$(*=O);+0:1][O+0H:2].[CX4!H0,c;+0:3][F,Cl,Br,I;+0:4])>>[*:1][*:2][*:3].[*:4]",

**Ether Cleavage**

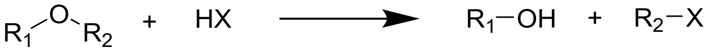

"[C,c;+0;!$(*=O):1][O+0:2]!@[C;+0;!$(*=O):3].[F,Cl,Br,I;+0H:4]>>[*:1][*:2].[*:3][*:4]",

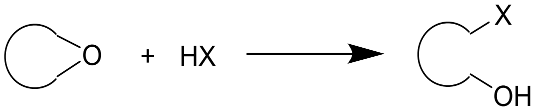

"[C,c;+0;!$(*=O):1][O+0:2]@[C;+0;!$(*=O):3].[F,Cl,Br,I;+0H:4]>>([*:1][*:2].[*:3][*:4])",

**Ether Synthesis by Dehydration**

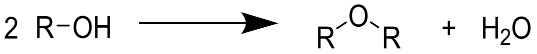

"[C,c;!$(*=O);+0:1][O+0H:2].[C,c;!$(*=O);+0:3][O+0H:4]>>[*:1][*:2][*:3].[*:4]",

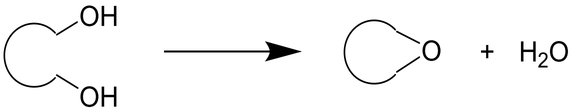

"([C,c;!$(*=O);+0:1][O+0H:2].[C,c;!$(*=O);+0:3][O+0H:4])>>[*:1][*:2][*:3].[*:4]",

**Epoxides Ring Opening**

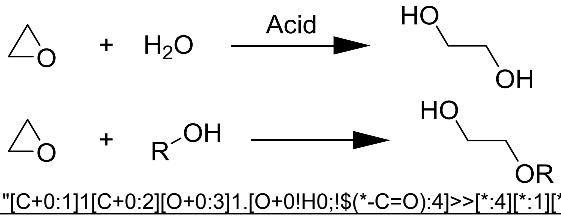

"[C+0:1]1[C+0:2][O+0:3]1.[O+0!H0;!$(*-C=O):4]>>[*:4][*:1][*:2][*:3]",

# Aldehydes and Ketones

| | |
|---|---|
| Aldehyde Oxidation | 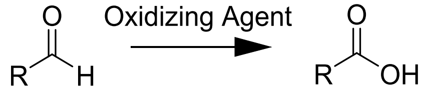 "[C!$(*[OH])+0!H0:1]=[O+0:2].[O+0:3]=[O+0]>>[*:1](=[*:2])[*:3]", |
| Aldehyde Oxidation, 2-step | 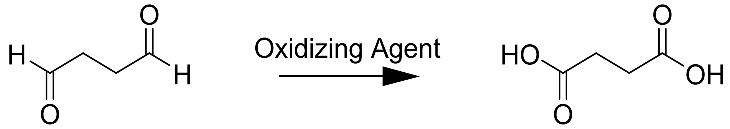 "([C+0!H0:1]=[O+0:2].[C+0!H0:5]=[O+0:6]).[O+0:3]=[O+0:4]>>([*:1](=[*:2])[*:3].[*:5](=[*:6])[*:4])", |
| Aldehyde & Alcohol Oxidation | 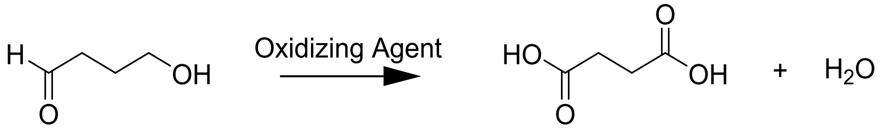 "([C+0!H0:1]=[O+0:2].[C+0;H3,H2:5][O+0H:6]).[O+0:3]=[O+0:4]>>([*:1](=[*:2])[*:3].[*:5](=[*:6])[O]).[*:4]", |
| Ketone Oxidation | 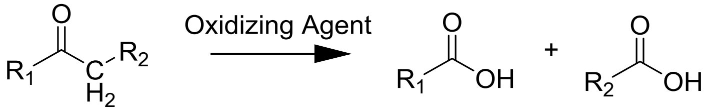 "[C,c;+0:1][C+0:2](=[O+0:3])!@[C+0H2:4][C,c;+0:5].[O+0:6]=[O+0:7]>>[*:1][*:2](=[*:3])[*:6].[*:5][*:4](=[*:7])[O]", 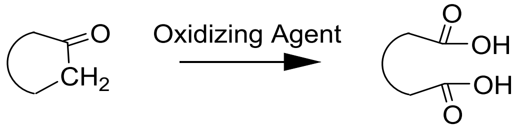 "[C,c;+0:1][C+0:2](=[O+0:3])@[C+0H2:4][C,c;+0:5].[O+0:6]=[O+0:7]>>([*:1][*:2](=[*:3])[*:6].[*:5][*:4](=[*:7])[O])", |
| Baeyer-Villiger Oxidation (ketones and aldehydes) | 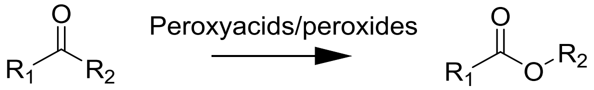 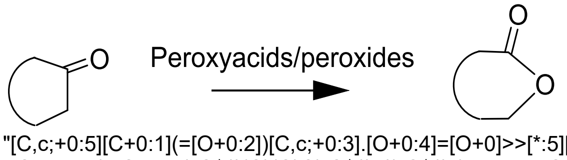 "[C,c;+0:5][C+0:1](=[O+0:2])[C,c;+0:3].[O+0:4]=[O+0]>>[*:5][*:1](=[*:2])[*:4][*:3]", Baeyer-Villiger "[C+0H:1](=[O+0:2])[C$(*(C)(C)C),C$(*=*),C$(*c),c;+0:3].[O+0:4]=[O+0]>>[*:1](=[*:2])[*:4][*:3]", |
| Hydrogenation of Ketones | 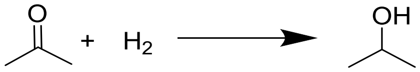 "[C+0!$(*-O):1]=[O+0:2].[H][H]>>[*:1][*:2]", |
| Keto-enol Tautomerization (Also covers Imine-Enamine, Imine-Amine, Amide-Imidic Acid Tautomerization) | 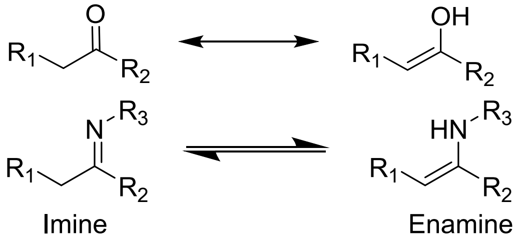 |

| | |
|---|---|
| | 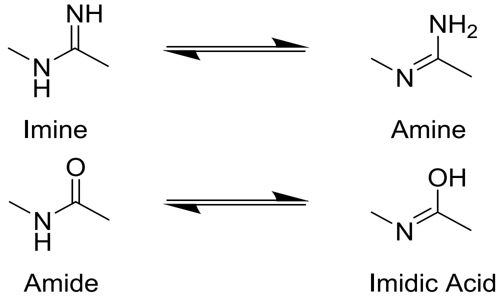<br>"[C,N;+0!H0:1][C+0:2]=[O,N;+0:3]>>[*:1]=[*:2][*:3]", |
| Decarbonylation of Aldehydes | 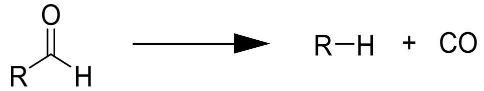<br>"[*+0:1][C+0H:2]=[O+0:3]>>[*:1].[*-:2]#[*+:3]", |
| Ketonization | 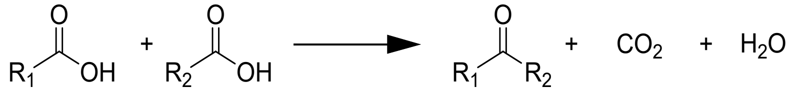<br>"[*+0:1][C+0:2](=[O+0:3])[O+0H:4].[*+0:5][C+0:6](=[O+0:7])[O+0H:8]>>[*:1][*:2](=[*:3])[*:5].[*:4]=[*:6]=[*:7].[*:8]",<br>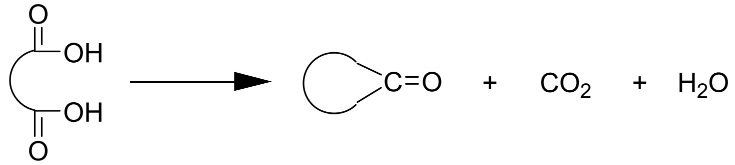<br>"([*+0:1][C+0:2](=[O+0:3])[O+0H:4].[*+0:5][C+0:6](=[O+0:7])[O+0H:8])>>[*:1][*:2](=[*:3])[*:5].[*:4]=[*:6]=[*:7].[*:8]", |
| Oxidative Esterification of Aldehydes and Alcohols | 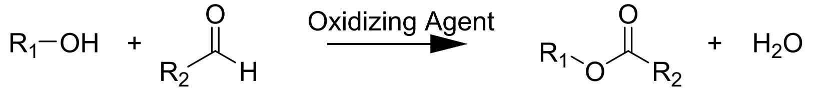<br>"[C+0;!$(*=O):5][O+0H:1].[O+0:2]=[C+0!H0:3].[O+0:4]=[O+0]>>[*:5][*:1][*:3]=[*:2].[*:4]", |
| | 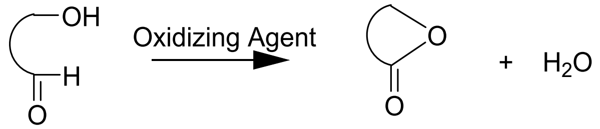<br>"([C+0;!$(*=O):5][O+0H:1].[O+0:2]=[C+0!H0:3]).[O+0:4]=[O+0]>>[*:5][*:1][*:3]=[*:2].[*:4]", |
| McMurry Reaction | 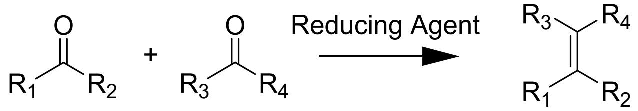<br>"[*+0:1][C+0;!$(*-O):2](=[O+0:3]).[*+0:5][C+0;!$(*-O):6](=[O+0:7]).[H][H]>>[*:1][*:2]=[*:6][*:5].[*:3].[*:7]",<br>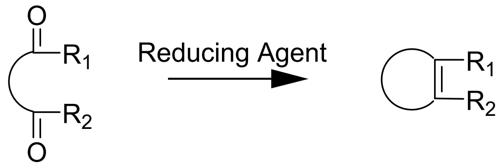<br>"([*+0:1][C+0;!$(*-O):2](=[O+0:3]).[*+0:5][C+0;!$(*-O):6](=[O+0:7])).[H][H]>>[*:1][*:2]=[*:6][*:5].[*:3].[*:7]", |

| | |
|---|---|
| Hydration of Ketone and Aldehyde | 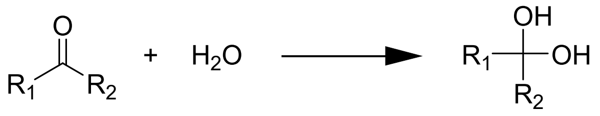<br>"[CX3+0;!$(*-O):2]=[O+0:3].[O+0H2:4]>>[*:2]([*:3])[*:4]", |
| Hemiacetal Dissociation and Formation, Addition of Alcohols to Carbonyl Groups | 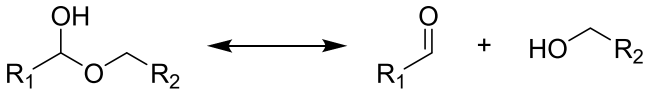<br>"[C;!$(*=O):1]([O+0H:2])!@[O+0:3][C;!$(*=O):4]>>[*:1]=[*:2].[*:3][*:4]",<br>"[C;!$(*O):1]=[O+0:2].[O+0H:3][C;!$(*=O):4]>>[*:1]([*:2])[*:3][*:4]",<br>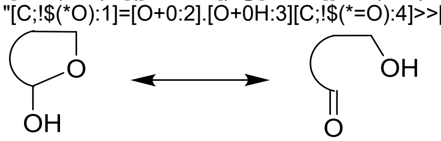<br>"[C;!$(*=O):1]([O+0H:2])@[O+0:3][C;!$(*=O):4]>>([*:1]=[*:2].[*:3][*:4])",<br>"([C;!$(*O):1]=[O+0:2].[O+0H:3][C;!$(*=O):4])>>[*:1]([*:2])[*:3][*:4]", |
| Reduction of Carbonyl Groups | 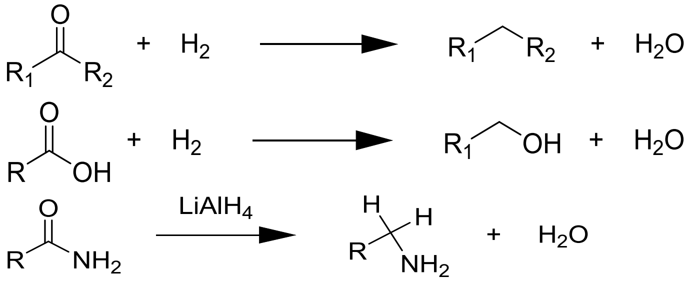<br>"[C,c,N;+0:1][C+0:2]=[O+0:3].[H][H]>>[*:1][*:2].[*:3]", |
| a-Halogenation | 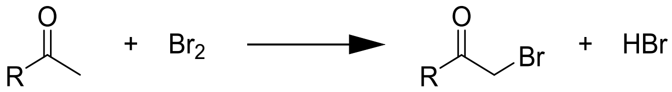<br>"[C+0:2](=[O+0:3])[C+0!H0:4].[F,Cl,Br,I;+0:5][F,Cl,Br,I;+0:6]>>[*:2](=[*:3])[*:4][*:5].[*:6]", |
| Wittig Reaction (Combined with Ylide Formation) | 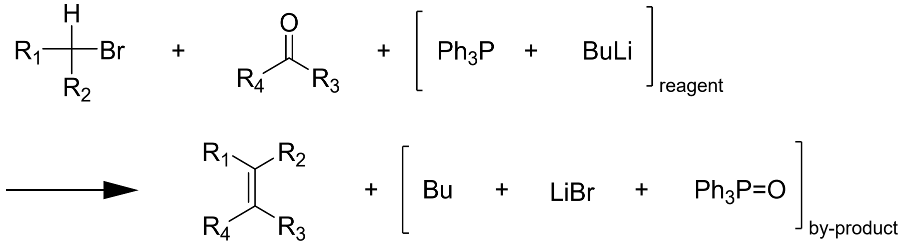<br>"[C+0!H0;!$(*~[O,S,N]):1][F,Cl,Br,I;+0].[C+0X3;!$(*-O):4]=[O+0]>>[*:1]=[*:4]",<br>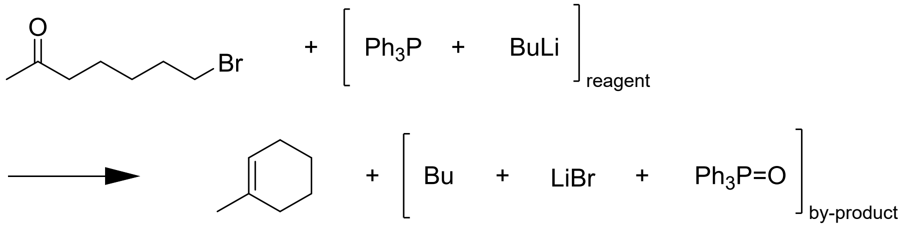<br>"([C+0!H0;!$(*~[O,S,N]):1][F,Cl,Br,I;+0].[C,c;+0:3][C+0;!$(*-O):4]=[O+0])>>[*:1]=[*:4][*:3]", |

| | |
|---|---|
| Synthesis of Enol Ethers from Aldehyde and Alcohol | 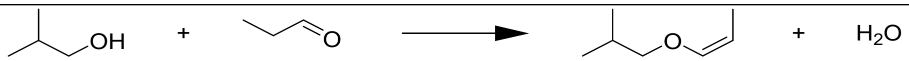 `"[CX4+0;H2,H3:1][O+0H:2].[CX4+0!H0:3][CX3+0H:4]=[O+0:5]>>[*:1][*:2][*:4]=[*:3].[*:5]",` 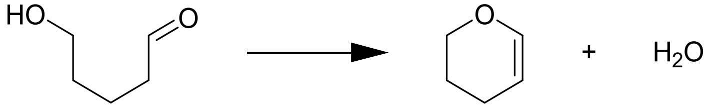 `"([CX4+0;H2,H3:1][O+0H:2].[CX4+0!H0:3][CX3+0H:4]=[O+0:5])>>[*:1][*:2][*:4]=[*:3].[*:5]",` |
| Pinacol Coupling | 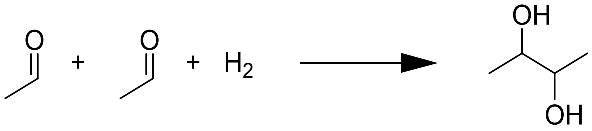 `"[C+0X3;!$(*-O):2](=[O+0:3]).[C+0X3;!$(*-O):6](=[O+0:7]).[H][H]>>[*:2](-[*:3])-[*:6]-[*:7]",` 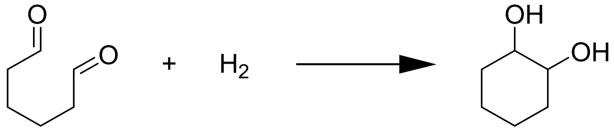 `"([C+0X3;!$(*-O):2](=[O+0:3]).[C+0X3;!$(*-O):6](=[O+0:7])).[H][H]>>[*:2](-[*:3])-[*:6]-[*:7]",` |
| **Carboxylic Acids** | |
| Carboxylic Acids Decarboxylation | 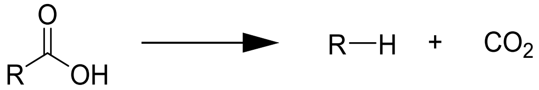 `"[*+0:1][C+0:2](=[O+0:3])[O+0H:4]>>[*:1].[*:3]=[*:2]=[*:4]",` |
| Acid Anhydrides React with Alcohols to Form Esters | 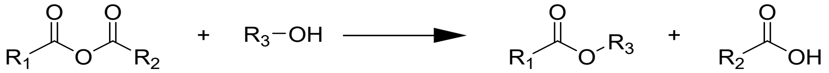 `"[C+0;!$(*[OH]):1](=[O+0:2])[O+0!R:3][C+0;!$(*[OH]):4](=[O+0:5]).[C,c;+0;!$(*=O):6][O+0H:7]>>[*:1](=[*:2])[*:3][*:6].[*:4](=[*:5])[*:7]",` 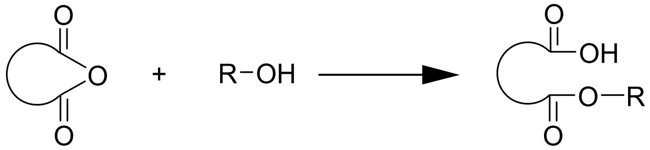 `"[C+0;!$(*[OH]):1](=[O+0:2])[O+0R:3][C+0;!$(*[OH]):4](=[O+0:5]).[C,c;+0;!$(*=O):6][O+0H:7]>>([*:1](=[*:2])[*:3][*:6].[*:4](=[*:5])[*:7])",` |
| Oxidation of Butane | 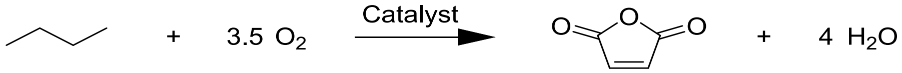 `"[C+0H3][C+0H2][C+0H2][C+0H3].[O+0]=[O+0]>>[C]1(=[O])[C]=[C][C](=[O])[O]1.[O]",` |
| **Enols and Enolates** | |
| Aldol Condensation | 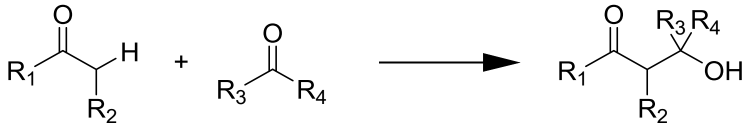 `"[C!$(*[OH])+0:2](=[O+0:3])[C+0!H0:4].[O+0:5]=[C!$(*[OH])+0X3:6]>>[*:2](=[*:3])[*:4][*:6]([*:5])",` 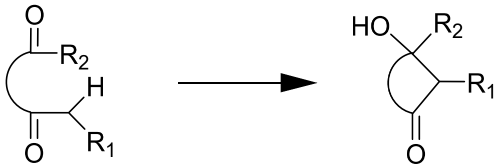 `"([C!$(*[OH])+0:2](=[O+0:3])[C+0!H0:4].[O+0:5]=[C!$(*[OH])+0X3:6])>>[*:2](=[*:3])[*:4][*:6]([*:5])",` |

| | |
|---|---|
| | 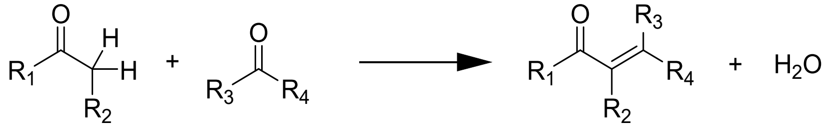<br>"[C!$(*[OH])+0:2](=[O+0:3])[C+0;H3,H2:4].[O+0:5]=[C!$(*[OH])+0X3:6]>>[*:2](=[*:3])[*:4]=[*:6].[*:5]",<br>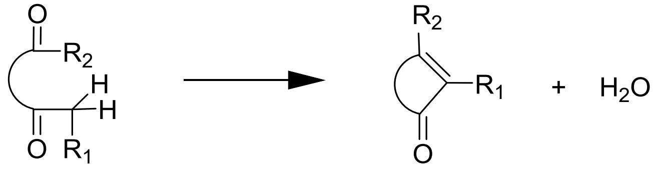<br>"([C!$(*[OH])+0:2](=[O+0:3])[C+0;H3,H2:4].[O+0:5]=[C!$(*[OH])+0X3:6])>>[*:2](=[*:3])[*:4]=[*:6].[*:5]", |
| Claisen Condensation | 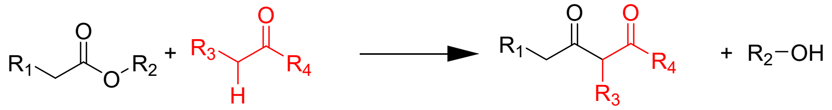<br>"[C!$(*[OH])+0:2](=[O+0:3])!@[O+0:7][C+0:4].[O+0:5]=[C!$(*[OH])+0:6][C+0!H0:8]>>[*:2](=[*:3])[*:8][*:6]=[*:5].[*:7][*:4]", |
| Dieckmann Condensation (Intramolecular Claisen Condensations) | 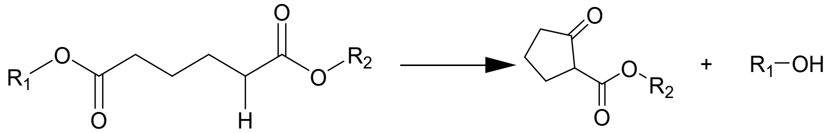<br>"([C!$(*[OH])+0:2](=[O+0:3])!@[O+0:7][C+0:4].[O+0:5]=[C!$(*[OH])+0:6][C+0!H0:8])>>[*:2](=[*:3])[*:8][*:6]=[*:5].[*:7][*:4]", |
| Michael Reaction | 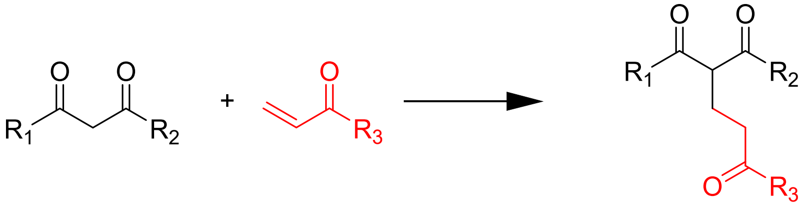<br>"[C+0:1](=[O+0:2])[C+0!H0:3][C+0:4]=[O+0:5].[C+0:6]=[C+0:7][C+0:8]=[O+0:9]>>[*:1](=[*:2])[*:3]([*:6][*:7][*:8]=[*:9])[*:4]=[*:5]",<br>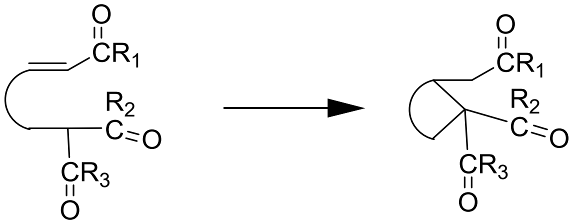<br>"([C+0:1](=[O+0:2])[C+0!H0:3][C+0:4]=[O+0:5].[C+0:6]=[C+0:7][C+0:8]=[O+0:9])>>[*:1](=[*:2])[*:3]([*:6][*:7][*:8]=[*:9])[*:4]=[*:5]",<br>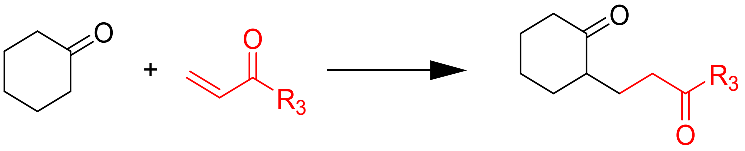<br>"[C+0:1]1(=[O+0:2])[C+0!H0:3][C+0:10][C+0:11][C+0:12][C+0:13]1.[C+0:6]=[C+0:7][C+0:8]=[O+0:9]>>[*:1]1(=[*:2])[*:3]([*:10][*:11][*:12][*:13]1)[*:6][*:7][*:8]=[*:9]",<br>"([C+0:1]1(=[O+0:2])[C+0!H0:3][C+0:10][C+0:11][C+0:12][C+0:13]1.[C+0:6]=[C+0:7][C+0:8]=[O+0:9])>>[*:1]1(=[*:2])[*:3]([*:10][*:11][*:12][*:13]1)[*:6][*:7][*:8]=[*:9]", |

### Robinson Annulation (Michael-aldol sequence)

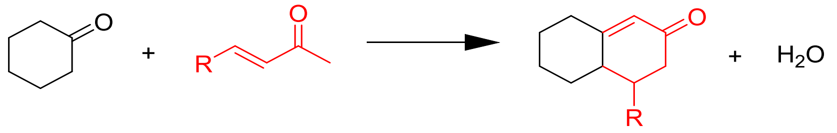

"[C+0:1]1(=[O+0:2])[C+0!H0:3][C+0:14][C+0:15][C+0:16][C+0:17]1.[C+0:8]=[C+0:9][C+0:10](=[O +0:11])[C+0;H2,H3:12]>>[*:1]12[*:3]([*:8][*:9][*:10](=[*:11])[*:12]=2)[*:14][*:15][*:16][*:17]1.[*:2]",

## Esters

### Esterification, Acid Anhydride Formation

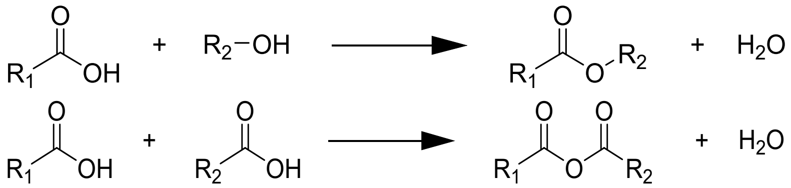

"[C+0:2](=[O+0:3])[O+0H:4].[C,c;+0:5][O+0H:6]>>[*:2](=[*:3])[*:6][*:5].[*:4]",

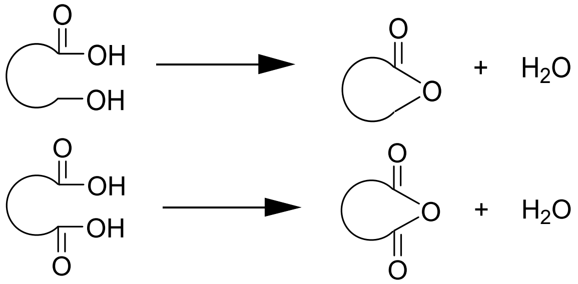

"([C+0:2](=[O+0:3])[O+0H:4].[C,c;+0:5][O+0H:6])>>[*:2](=[*:3])[*:6][*:5].[*:4]",

### Esterification of Acid Halides

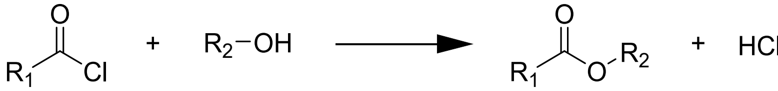

"[C+0:1](=[O+0:2])[F,Cl,Br,I;+0:3].[C,c;!$(*=O);+0:4][O+0H:5]>>[*:1](=[*:2])[*:5][*:4].[*:3]",

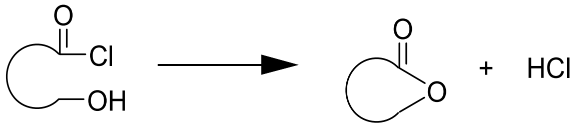

"([C+0:1](=[O+0:2])[F,Cl,Br,I;+0:3].[C,c;!$(*=O);+0:4][O+0H:5])>>[*:1](=[*:2])[*:5][*:4].[*:3]",

### Ester Reduction

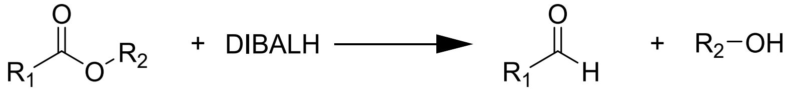

"[O+0H0!R:1][C+0:2]=[O+0:3].[H][H]>>[*:1].[*:2]=[*:3]",

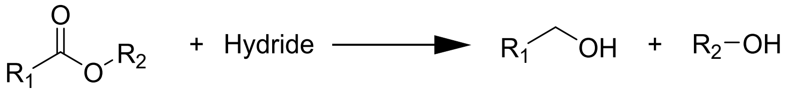

"[O+0H0!R:1][C+0:2]=[O+0:3].[H][H]>>[*:1].[*:2][*:3]",

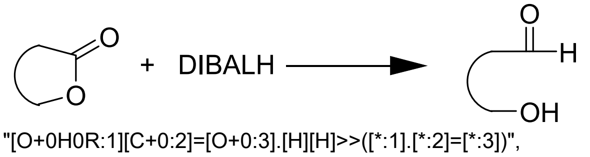

"[O+0H0R:1][C+0:2]=[O+0:3].[H][H]>>([*:1].[*:2]=[*:3])",

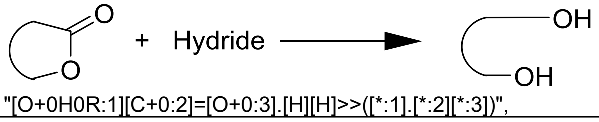

"[O+0H0R:1][C+0:2]=[O+0:3].[H][H]>>([*:1].[*:2][*:3])",

**Transesterification**

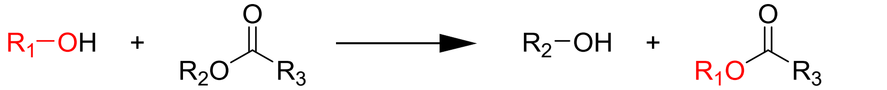


"[C+0:1](=[O+0:2])!@[O+0:3][*+0:4].[*+0:6][O+0H;!$(*-C=O):5]>>[*:1](=[*:2])[*:5][*:6].[*:4][*:3]",

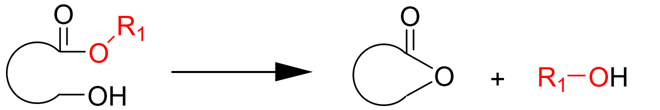


"([C+0:1](=[O+0:2])!@[O+0:3][*+0:4].[*+0:6][O+0H;!$(*-C=O):5])>>[*:1](=[*:2])[*:5][*:6].[*:4][*:3]",

# Ketenes

**[2+2] Cycloaddition**

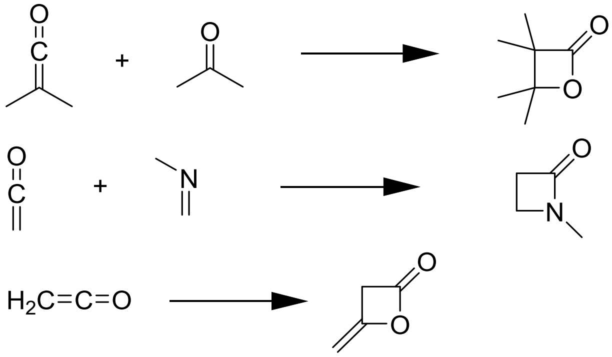


"[C+0:1]=[C+0:2]=[O+0:3].[C!$(*(=O)=O)!$(*-O)+0:4]=[O,N;+0:5]>>[*:1]1[*:2](=[*:3])[*:5][*:4]1",

# Benzene and Derivatives

**Benzene Hydrogenation**

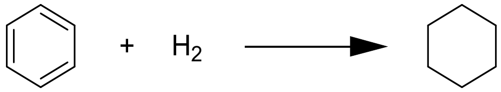


"[C:1]1=[C:2][C:3]=[C:4][C:5]=[C:6]1.[H][H]>>[*:1]1[*:2][*:3][*:4][*:5][*:6]1",

**Benzene Partial Hydrogenation**

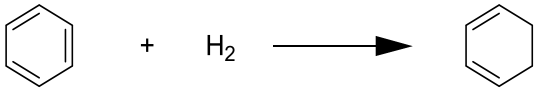


"[C:1]1=[C:2][C:3]=[C:4][C:5]=[C:6]1.[H][H]>>[*:1]1=[*:2][*:3]=[*:4][*:5][*:6]1",

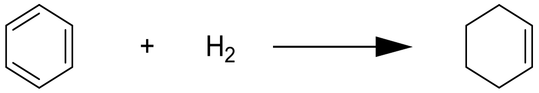


"[C:1]1=[C:2][C:3]=[C:4][C:5]=[C:6]1.[H][H]>>[*:1]1=[*:2][*:3][*:4][*:5][*:6]1",

**Kolbe Carboxylation**

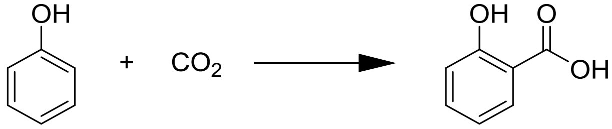


"[c+0:1]1:[c+0:2]([O+0H:7]):[c+0H:3]:[c+0:4]:[c+0:5]:[c+0:6]:1.[O+0:8]=[C+0:9]=[O+0:10]>>[*:1]1:[
*:2]([*:7]):[*:3]([*:9](=[*:8])[*:10]):[*:4]:[*:5]:[*:6]:1",

| | |
|---|---|
| Phenols Oxidation to Quinones | 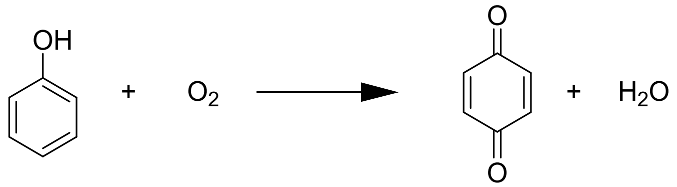<br>"[c+0;!$(*~O):1]1:[c+0:2]([O+0H:7]):[c+0;!$(*~O):3]:[c+0;!$(*~O):4]:[c+0H:5]:[c+0;!$(*~O):6]:1.[O+0:8]=[O+0:9]>>[*:1]1[*:2](=[*:7])[*:3]=[*:4][*:5](=[*:8])[*:6]=1.[*:9]",<br>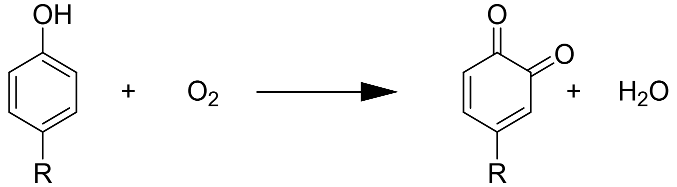<br>"[c+0;!$(*~O):1]1:[c+0:2]([O+0H:7]):[c+0H;!$(*~O):3]:[c+0;!$(*~O):4]:[c+0:5]([*;!O+0:10]):[c+0;!$(*~O):6]:1.[O+0:8]=[O+0:9]>>[*:1]1[*:2](=[*:7])[*:3](=[*:8])[*:4]=[*:5]([*:10])[*:6]=1.[*:9]",<br>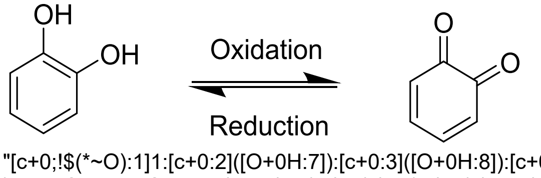<br>"[c+0;!$(*~O):1]1:[c+0:2]([O+0H:7]):[c+0:3]([O+0H:8]):[c+0;!$(*~O):4]:[c+0;!$(*~O):5]:[c+0;!$(*~O):6]:1.[O+0:9]=[O+0]>>[*:1]1[*:2](=[*:7])[*:3](=[*:8])[*:4]=[*:5][*:6]=1.[*:9]",<br>"[C+0:1]1[C+0:2](=[O+0:7])[C+0:3](=[O+0:8])[C+0:4]=[C+0:5][C+0:6]=1.[H][H]>>[*:1]1:[*:2]([*:7]):[*:3]([*:8]):[*:4]:[*:5]:[*:6]:1",<br>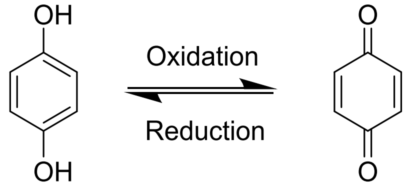<br>"[c+0;!$(*~O):1]1:[c+0:2]([O+0H:7]):[c+0;!$(*~O):3]:[c+0;!$(*~O):4]:[c+0:5]([O+0H:8]):[c+0;!$(*~O):6]:1.[O+0:9]=[O+0]>>[*:1]1[*:2](=[*:7])[*:3]=[*:4][*:5](=[*:8])[*:6]=1.[*:9]",<br>"[C+0:1]1[C+0:2](=[O+0:7])[C+0:3]=[C+0:4][C+0:5](=[O+0:8])[C+0:6]=1.[H][H]>>[*:1]1:[*:2]([*:7]):[*:3]:[*:4]:[*:5]([*:8]):[*:6]:1", |
| Quinones Addition | 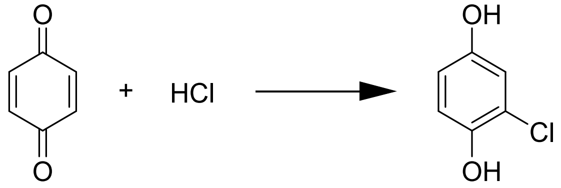<br>"[C+0:1]1[C+0:2](=[O+0:7])[C+0:3]=[C+0H:4][C+0:5](=[O+0:8])[C+0:6]=1.[F,Cl,Br,I;+0H:9]>>[*:1]1:[*:2]([*:7]):[*:3]:[*:4]([*:9]):[*:5]([*:8]):[*:6]:1", |
| Naphthalene Oxidation | 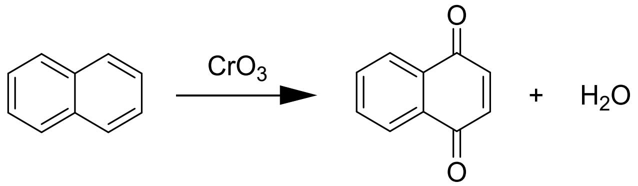<br>"[c+0:1]1:[c+0H:2]:[c+0:3]([c+0:9][c+0:10][c+0:11][c+0:12]2):[c+0:4]2:[c+0H:5]:[c+0:6]:1.[O+0:7]=[O+0:8]>>[*:1]1[*:2](=[*:7])[*:3](:[*:9]:[*:10]:[*:11]:[*:12]:2)=[*:4]2[*:5](=[O])[*:6]=1.[*:8]",<br>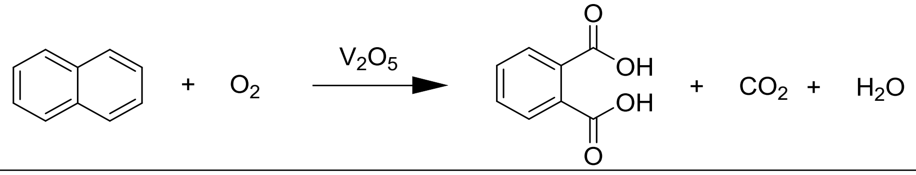 |

| | |
|---|---|
| | "[c+0:1]1:[c+0:2]:[c+0:3]([c+0H:9][c+0H:10][c+0H][c+0H:12]2):[c+0:4]2:[c+0:5]:[c+0:6]:1.[O+0:7]=[O+0:8]>>[*:1]1:[*:2]:[*:3]([C:9](=[O])[O]):[*:4]([C:12](=[O])[O]):[*:5]:[*:6]:1.[*:7]=[C:10]=[O].[*:8]", |
| Oxidation of Aromatic Alkanes | 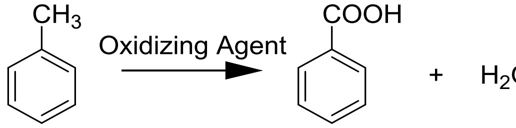<br>"[c+0:1][C+0H3:2].[O+0:3]=[O+0:4]>>[*:1][*:2](=[*:3])[O].[*:4]",<br>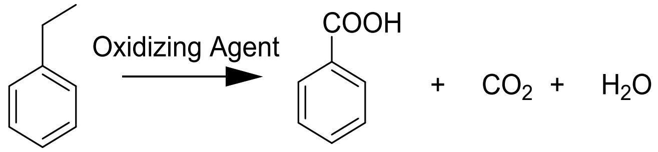<br>"[c+0:1][C+0H2:2][C+0H3:3].[O+0:4]=[O+0:5]>>[*:1][*:2](=[*:4])[O].[O]=[*:3]=[O].[*:5]",<br>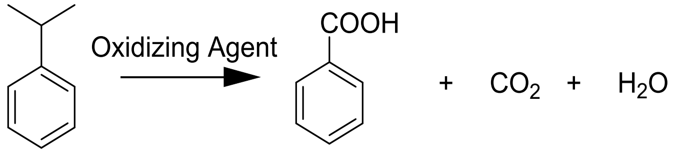<br>"[c+0:1][C+0H:2]([C+0H3:3])[C+0H3].[O+0:4]=[O+0:5]>>[*:1][*:2](=[*:4])[O].[O]=[*:3]=[O].[*:5]",<br>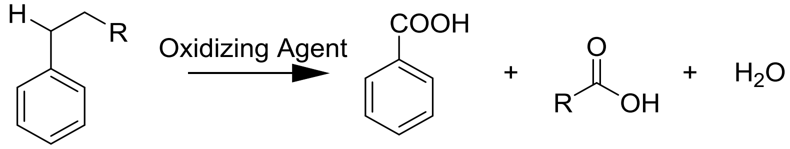<br>"[c+0:1][C+0H2:2]!@[C+0H2:3][C+0!H3:4].[O+0:5]=[O+0:6]>>[*:1][*:2](=[*:5])[O].[*:4][*:3](=[O])[O].[*:6]",<br>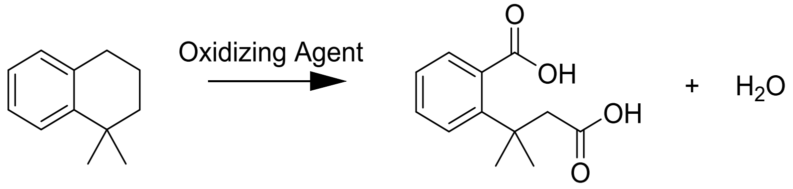<br>"[c+0:1][C+0H2:2]@-[C+0H2:3].[O+0:4]=[O+0:5]>>([*:1][*:2](=[*:4])[O].[*:3](=[O])[O]).[*:5]",<br>"[c+0:1][C+0H:2]@=[C+0H:3].[O+0:4]=[O+0:5]>>([*:1][*:2](=[*:4])[O].[*:3](=[*:5])[O])", |
| Oxidation of Aromatic Alkanes, 2-step | 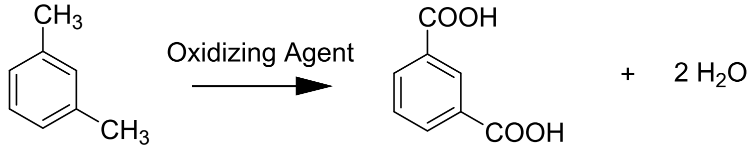<br>"([c+0:1][C+0H3:2].[c+0:5][C+0H3:6]).[O+0:3]=[O+0:4]>>([*:1][*:2](=[*:3])[O].[*:5][*:6](=[O])[O]).[*:4]", |
| Aldehyde & Aromatic Alkane Oxidation | 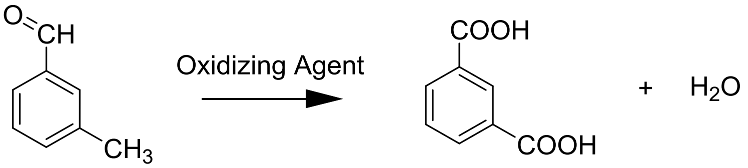<br>"([C+0!H0:1]=[O+0:2].[c+0:5][C+0H3:6]).[O+0:3]=[O+0:4]>>([*:1](=[*:2])[*:3].[*:5][*:6](=[O])[O]).[*:4]", |
| Alcohol & Aromatic Alkane Oxidation | 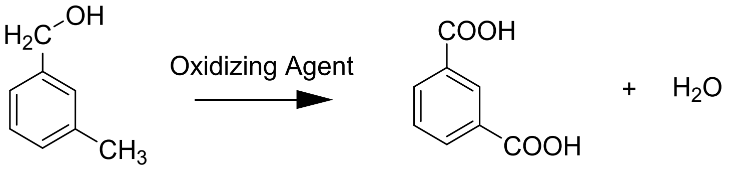<br>"([C+0;H3,H2:1][O+0H:2].[c+0:5][C+0H3:6]).[O+0:3]=[O+0:4]>>([*:1](=[*:2])[*:3].[*:5][*:6](=[O])[O] |

`).[*:4]",`

### Halogenation of Aromatic Alkanes

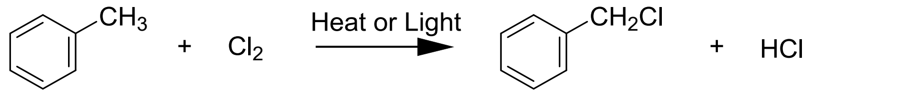


`"[c+0:1][C+0!H0:2].[F,Cl,Br,I;+0:3][F,Cl,Br,I;+0:4]>>[*:1][*:2][*:3].[*:4]",`

### Electrophilic Aromatic Alkylation with Alkenes

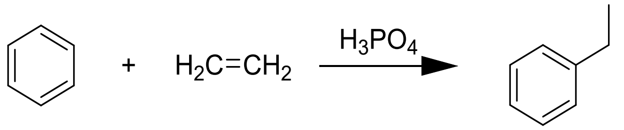


`"[c+0H:1].[C+0:2]=[C+0:3]>>[*:1][*:2][*:3]",`

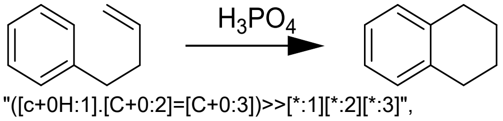


`"([c+0H:1].[C+0:2]=[C+0:3])>>[*:1][*:2][*:3]",`

### Electrophilic Aromatic Alkylation with Alcohols

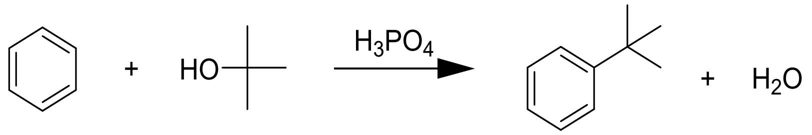


`"[c+0H:1].[C+0;!$(*=O):2][O+0H:3]>>[*:1][*:2].[*:3]",`

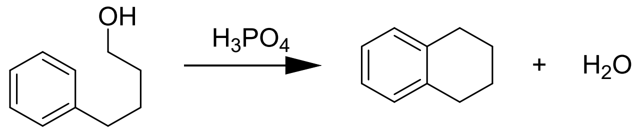


`"([c+0H:1].[C+0;!$(*=O):2][O+0H:3])>>[*:1][*:2].[*:3]",`

### Furan Carboxylation

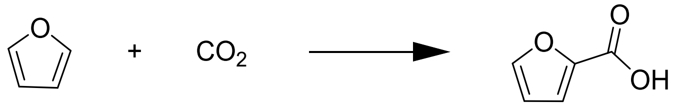


`"[o+0:1]1:[c+0H:2]:[c+0:3]:[c+0:4]:[c+0:5]:1.[O+0:8]=[C+0:9]=[O+0:10]>>[*:1]1:[*:2]([*:9](=[*:8])[*:10]):[*:3]:[*:4]:[*:5]:1",`

### Furan Carboxylation, 2-step

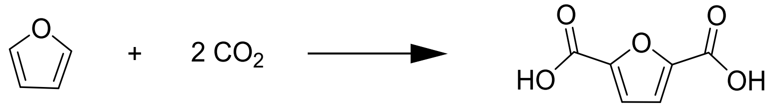


`"[o+0:1]1:[c+0H:2]:[c+0:3]:[c+0:4]:[c+0H:5]:1.[O+0:8]=[C+0:9]=[O+0:10]>>[*:1]1:[*:2]([*:9](=[*:8])[*:10]):[*:3]:[*:4]:[*:5]([C](=[O])[O]):1",`

### Friedel-Crafts Hydroxyalkylation

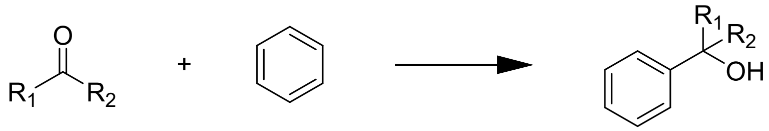


`"[c+0H:1].[CX3!$(*-O)+0:2]=[O+0:3]>>[*:1][*:2][*:3]",`

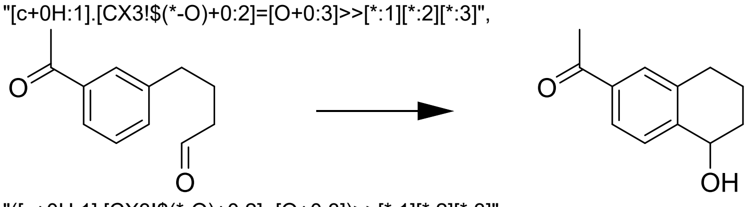


`"([c+0H:1].[CX3!$(*-O)+0:2]=[O+0:3])>>[*:1][*:2][*:3]",`

| | |
|---|---|
| | 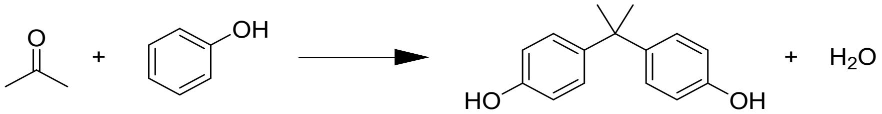<br>"[c+0H:1].[c+0H:2].[CX3!$(*-O)+0:3]=[O+0:4]>>[*:1][*:3][*:2].[*:4]", |
| Hock Process | 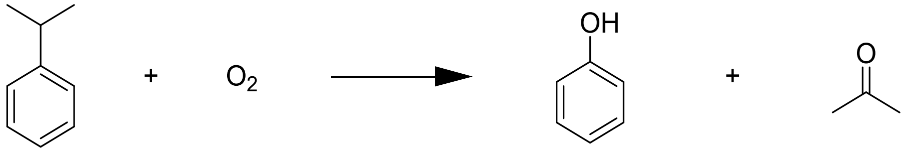<br>"[c+0:1][C+0H:2]([C+0H3:3])[C+0H3:4].[O+0:5]=[O+0:6]>>[*:1][*:5].[*:3][*:2](=[*:6])[*:4]", |
| Dehydrogenation of Ethylbenzene | 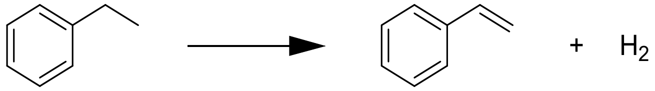<br>"[cH]1[cH][cH][cH][cH][c]1[CH2][CH3]>>[c]1[c][c][c][c][c]1[C]=[C].[H][H]", |
| **Carbonylation** | |
| Hydroformylation | 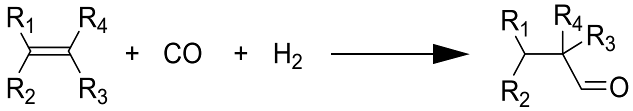<br>"[C+0:1]=[C+0:2].[C-1]#[O+1].[H][H]>>[*:1][*:2][C]=[O]", |
| Cativa Process | 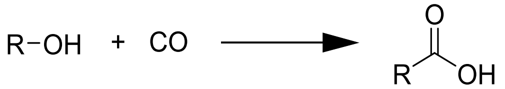<br>"[C!$(*=O)+0:1][O+0H:2].[C-1]#[O+1]>>[*:1][C](=[O])[*:2]", |
| Oxidative Carbonylation | 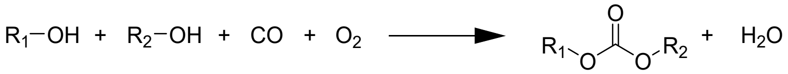<br>"[C!$(*=O)+0:1][O+0H:2].[C!$(*=O)+0:5][O+0H:6].[C-1]#[O+1].[O+0:7]=[O+0]>>[*:1][*:2][C](=[O])[*:6][*:5].[*:7]",<br>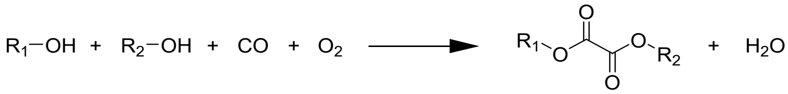<br>"[C!$(*=O)+0:1][O+0H:2].[C!$(*=O)+0:5][O+0H:6].[C-1]#[O+1].[O+0:7]=[O+0]>>[*:1][*:2][C](=[O])[C](=[O])[*:6][*:5].[*:7]", |
| Hydrocarboxylation, Hydroesterification (Carboalkoxylation) | 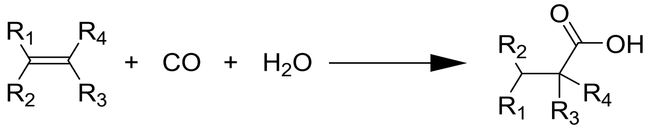<br>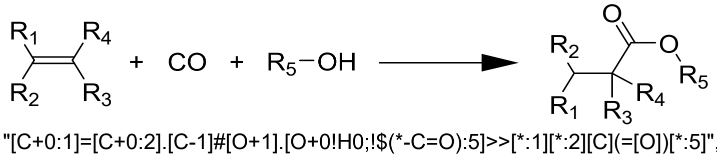<br>"[C+0:1]=[C+0:2].[C-1]#[O+1].[O+0!H0;!$(*-C=O):5]>>[*:1][*:2][C](=[O])[*:5]",<br>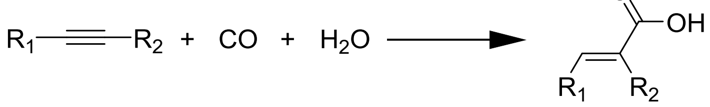<br>"[C+0:1]#[C+0:2].[C-1]#[O+1].[O+0H2:5]>>[*:1]=[*:2][C](=[O])[*:5]", |

**Hydrocarboxylation, 2-step**

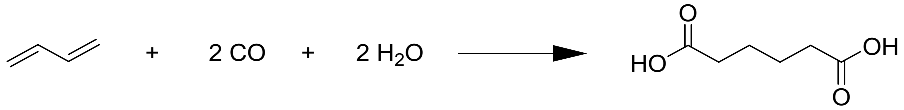

"([C+0:1]=[C+0:2].[C+0:3]=[C+0:4]).[C-1]#[O+1].[O+0!H0;!$(*-C=O):5]>>([*:1][*:2][C](=[O])[*:5].[*:3][*:4][C](=[O])[*:5])",

**Tennessee Eastman Acetic Anhydride Process**

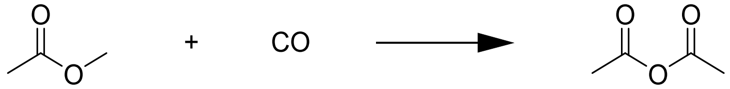

"[C+0:1](=[O+0:2])[O+0:3][C+0:4].[C-1]#[O+1]>>[*:1](=[*:2])[*:3][C](=[O])[*:4]",

**Reppe Process**

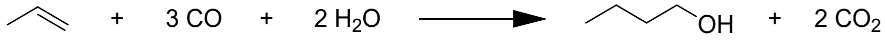

"[C+0:1]=[C+0:2].[C-1]#[O+1].[O+0H2:3]>>[*:1][*:2][C][*:3].[O]=[C]=[O]",

# Carbohydrates

**Oxidation of Pyranosides**

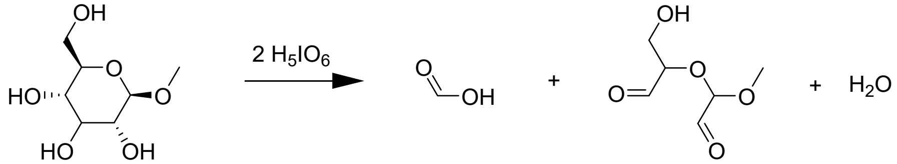

"[C+0:9][C+0H:1]([O+0H:2])@[C+0H:3]([O+0H:4])[C+0H:5]([O+0H:6])[C+0:10].[O+0:7]=[O+0:8]>>([*:9][*:1]=[*:2].[*:10][*:5]=[*:6]).[*:7][*:3]=[*:4].[*:8]",

# Amines, Amides

**Dehydration of Amides**

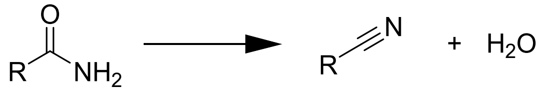

"[C+0:1](=[O+0:2])[N+0H2:3]>>[*:1]#[*:3].[*:2]",

**Ketone Reductive Amination**

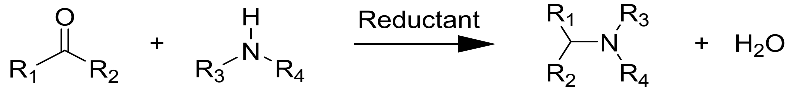

"[CX3!$(*[O,S,N])+0:2](=[O+0:3]).[N+0X3!H0:6].[H][H]>>[*:2][*:6].[*:3]",

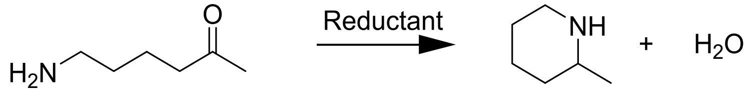

"([CX3!$(*[O,S,N])+0:2](=[O+0:3]).[N+0X3!H0:6]).[H][H]>>[*:2][*:6].[*:3]",

**Alkylation or Acylation of Amines (Including Buchwald–Hartwig Amination)**

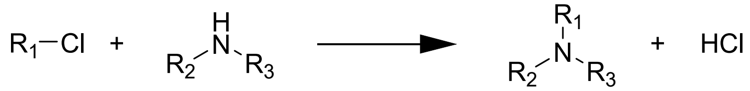

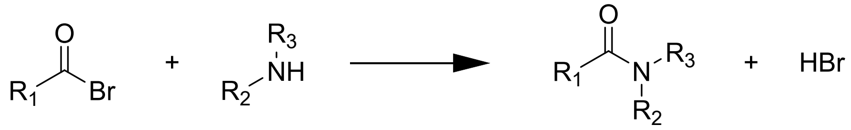

"[C,c;+0:1][F,Cl,Br,I;+0:2].[NX3!H0,nH;+0:3]>>[*:1][*:3].[*:2]",

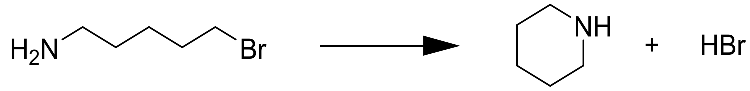

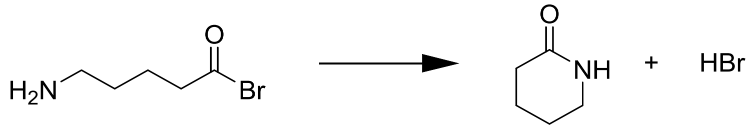

"([C,c;+0:1][F,Cl,Br,I;+0:2].[NX3!H0,nH;+0:3])>>[*:1][*:3].[*:2]",

Alkylation of Tertiary Amines

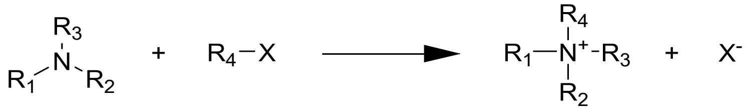

```
"[C,c;+0:1][F,Cl,Br,I;+0:2].[NX3H0+0:3]>>[*:1][*+1:3].[*-1:2]",
```

Amine Alkylation with Alcohols or Primary Amines

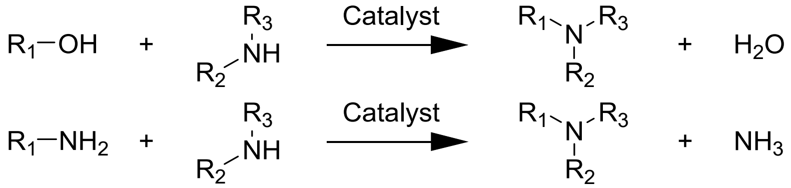

```
"[C,c;!$(*=O)+0:1][OH,NH2;+0:2].[NX3!H0,nH;+0:3]>>[*:1][*:3].[*:2]",
```

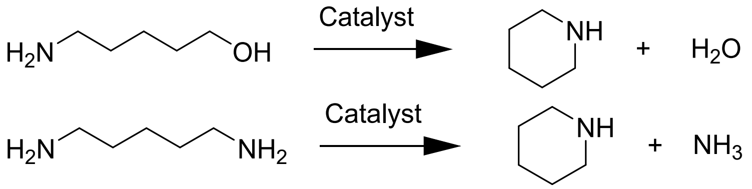

```
"([C,c;!$(*=O)+0:1][OH,NH2;+0:2].[NX3!H0,nH;+0:3])>>[*:1][*:3].[*:2]",
```

Synthesis of Amides with Carboxylic Acid

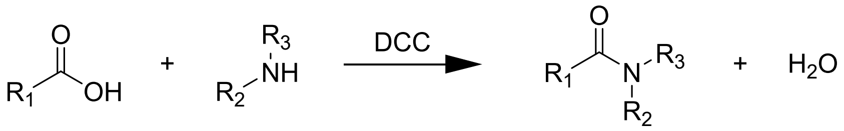

```
"[CX3+0:2](=[O+0:3])[O+0H:4].[N+0X3!H0:5]>>[*:2](=[*:3])[*:5].[*:4]",
```

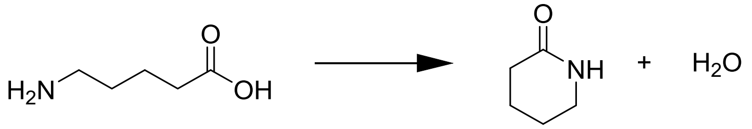

```
"([CX3+0:2](=[O+0:3])[O+0H:4].[N+0X3!H0:5])>>[*:2](=[*:3])[*:5].[*:4]",
```

Synthesis of Amides with Acid Anhydrides or Esters

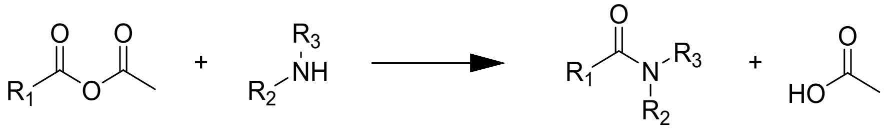

```
"[CX3+0:2](=[O+0:3])-!@[O+0:4][C,c;+0:6].[N+0X3!H0:5]>>[*:2](=[*:3])[*:5].[*:4][*:6]",
```

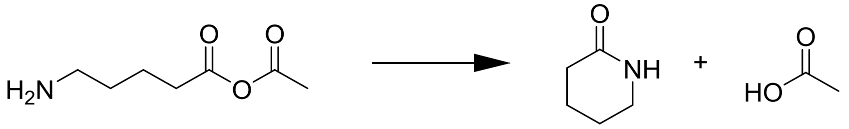

```
"([CX3+0:2](=[O+0:3])-!@[O+0:4][C,c;+0:6].[N+0X3!H0:5])>>[*:2](=[*:3])[*:5].[*:4][*:6]",
```

Hydrolysis of Amides

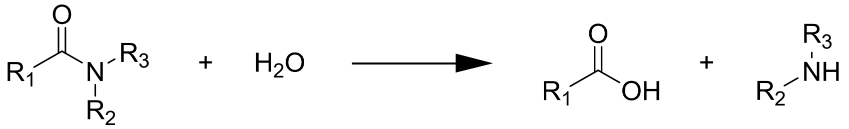

```
"[CX3!$(*[OH,SH])+0:2](=[O+0:3])-!@[N+0X3:5].[O+0H2:4]>>[*:2](=[*:3])[*:4].[*:5]",
```

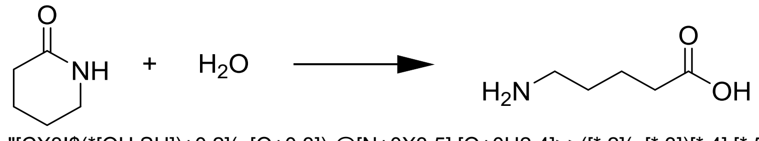

```
"[CX3!$(*[OH,SH])+0:2](=[O+0:3])-@[N+0X3:5].[O+0H2:4]>>([*:2](=[*:3])[*:4].[*:5])",
```

| | |
|---|---|
| Hofmann Elimination | 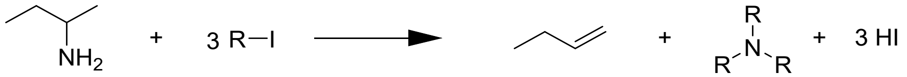<br>"[C+0X4!H0:1][CX4+0:2][N+0H2:3].[C+0:4][F,Cl,Br,I;+0:5]>>[*:1]=[*:2].[*:3]([*:4])([*:4])[*:4].[*:5]",<br>Hofmann<br>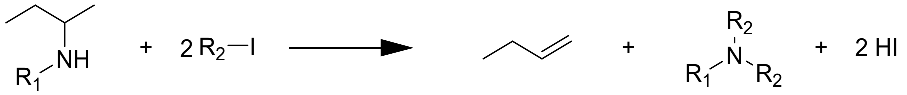<br>"[C+0X4!H0:1][CX4+0:2]-!@[N+0H:3].[C+0:4][F,Cl,Br,I;+0:5]>>[*:1]=[*:2].[*:3]([*:4])[*:4].[*:5]",<br>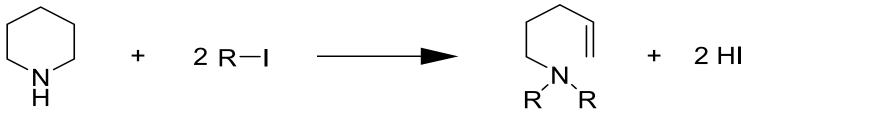<br>"[C+0X4!H0:1][CX4+0:2]-@[N+0H:3].[C+0:4][F,Cl,Br,I;+0:5]>>([*:1]=[*:2].[*:3]([*:4])[*:4]).[*:5]",<br>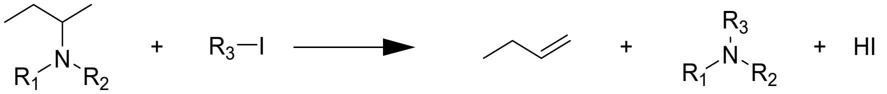<br>"[C+0X4!H0:1][CX4+0:2]-!@[N+0X3H0:3].[C+0:4][F,Cl,Br,I;+0:5]>>[*:1]=[*:2].[*:3][*:4].[*:5]",<br>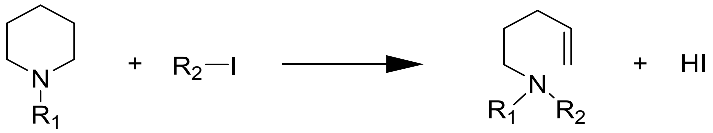<br>"[C+0X4!H0:1][CX4+0:2]-@[N+0X3H0:3].[C+0:4][F,Cl,Br,I;+0:5]>>([*:1]=[*:2].[*:3][*:4]).[*:5]", |
| Hofmann Rearrangement | 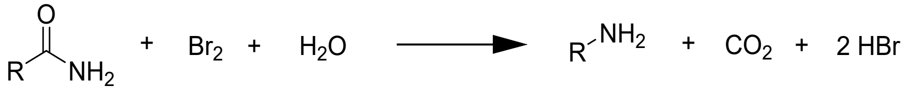<br>"[C,c;+0:1][C+0:2](=[O+0:3])[N+0H2:5].[F,Cl,Br,I;+0:6][F,Cl,Br,I;+0].[O+0H2:4]>>[*:1][*:5].[*:3]=[*:2]=[*:4].[*:6]",<br>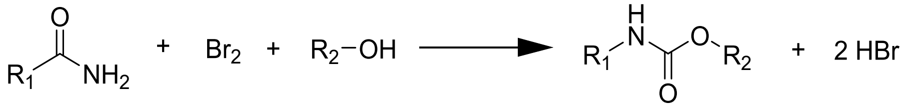<br>"[C,c;+0:1][C+0:2](=[O+0:3])[N+0H2:5].[F,Cl,Br,I;+0:6][F,Cl,Br,I;+0].[C+0!$(*=[O,S]):7][O+0H:4]>>[*:1][*:5][*:2](=[*:3])[*:4][*:7].[*:6]", |
| Imine-enamine Tautomerization (Covered in Keto-enol Tautomerization) | 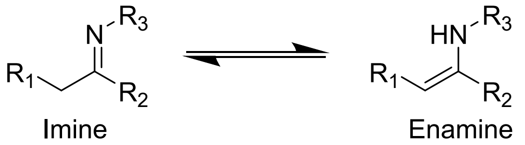 |
| Hydroamination of Alkenes | 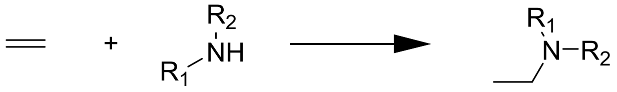<br>"[C+0:1]=[C+0:2].[N+0X3!H0:3]>>[*:1][*:2][*:3]",<br>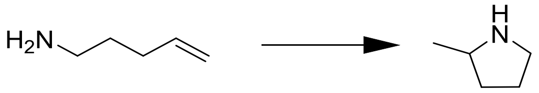<br>"([C+0:1]=[C+0:2].[N+0X3!H0:3])>>[*:1][*:2][*:3]", |
| Hydroamination of Alkynes | 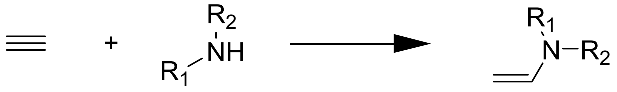<br>"[C+0:1]#[C+0:2].[N+0X3!H0:3]>>[*:1]=[*:2][*:3]", |

| | |
|---|---|
| | 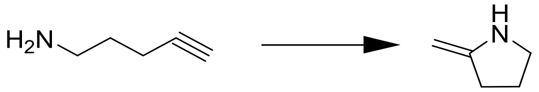<br>"([C+0:1]#[C+0:2].[N+0X3!H0:3])>>[*:1]=[*:2][*:3]", |
| Hydroamination of Dienes | 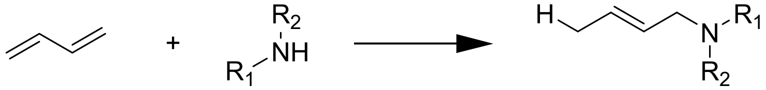<br>"[C+0:1]=[C+0:2][C+0:3]=[C+0:4].[N+0X3!H0:5]>>[*:1][*:2]=[*:3][*:4][*:5]",<br>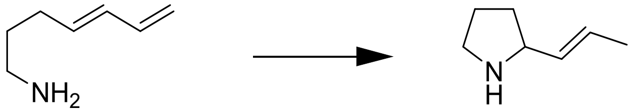<br>"([C+0:1]=[C+0:2][C+0:3]=[C+0:4].[N+0X3!H0:5])>>[*:1][*:2]=[*:3][*:4][*:5]", |
| Reduction of Amides to Amines (Covered in Reduction of Carbonyl Groups) | 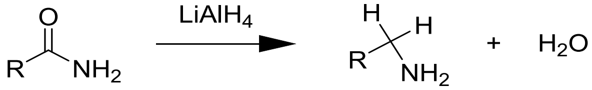 |
| Ring Opening of Epoxides by Amines | 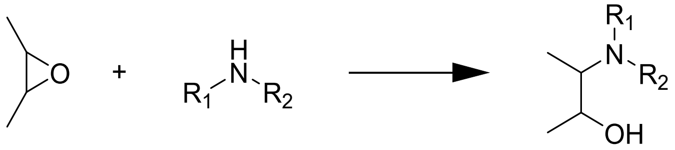<br>"[C+0:1]1[C+0:2][O+0:3]1.[N+0X3!H0:4]>>[*:4][*:1][*:2][*:3]", |
| Aromatic Nitrosation (with Amines and Hydroxyl Groups) | 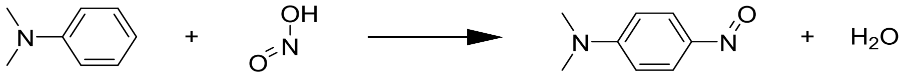<br>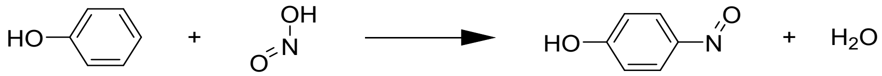<br>"[OH,N$(*([#6])([#6])[#6]);+0:10][c+0:1]1[c+0:2][c+0:3][c+0H:4][c+0:5][c+0:6]1.[O+0H:7][N+0:8]=[O+0:9]>>[*:10][*:1]1:[*:2]:[*:3]:[*:4]([*:8]=[*:9]):[*:5]:[*:6]:1.[*:7]", |
| Secondary Amines with Nitrous Acid | 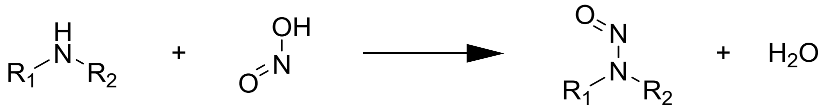<br>"[*+0:1][N+0H:2][*+0:3].[O+0H:4][N+0:5]=[O+0:6]>>[*:1][*:2]([*:5]=[*:6])[*:3].[*:4]", |
| Primary Amines with Nitrous Acid to Alcohols | 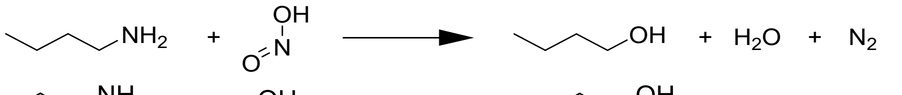<br>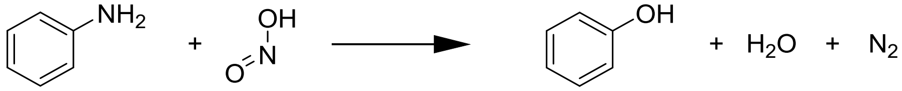<br>"[C,c;+0:1][N+0H2:2].[O+0H:3][N+0:4]=[O+0:5]>>[*:1][*:3].[*:5].[*:2]#[*:4]", |
| Primary Amines with Nitrous Acid to Halides (Sandmeyer Reaction) | 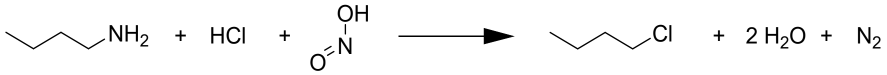<br>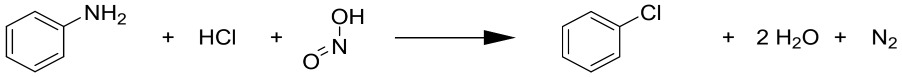<br>"[C,c;+0:1][N+0H2:2].[F,Cl,Br,I;+0H:3].[O+0H][N+0:5]=[O+0:6]>>[*:1][*:3].[*:6].[*:2]#[*:5]", |

| | |
|---|---|
| Primary Amines with Nitrous Acid to Alkenes | 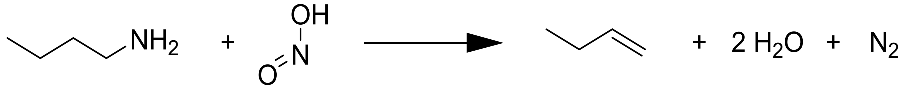<br>"[C+0!H0:6][C+0:1][N+0H2:2].[O+0H][N+0:4]=[O+0:5]>>[*:6]=[*:1].[*:5].[*:2]#[*:4]", |
| Tiffeneau–Demjanov Rearrangement | 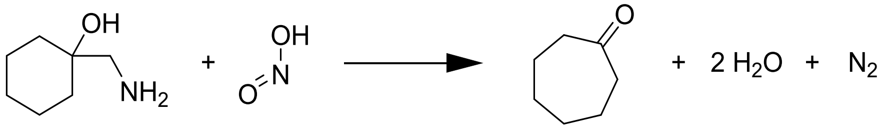<br>"[O+0H:1][C+0:2]([C+0:3])([C+0:4])[C+0:5][N+0H2:6].[O+0H][N+0:7]=[O+0:8]>>[*:1]=[*:2]([*:3])[*:5][*:4].[*:8].[*:6]#[*:7]", |
| Sandmeyer Cyanation | 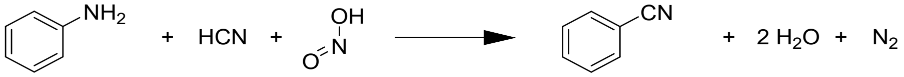<br>"[c+0:1][N+0H2:2].[C+0H:3]#[N+0:4].[O+0H][N+0:5]=[O+0:6]>>[*:1][*:3]#[*:4].[*:6].[*:2]#[*:5]", |
| Reduction of Primary Aromatic Amines | 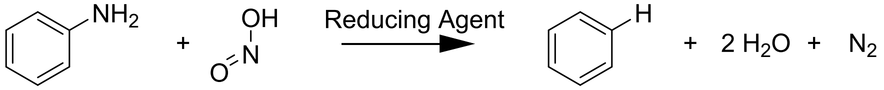<br>"[c+0:1][N+0H2:2].[H][H].[O+0H][N+0:3]=[O+0:4]>>[*:1].[*:4].[*:2]#[*:3]", |
| Cope Elimination | 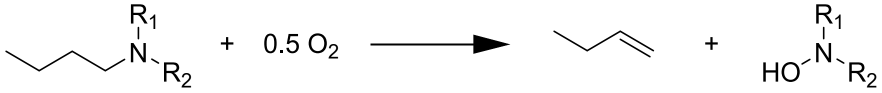<br>"[C+0!H0:1][C+0:2]!@[N+0:3]([C+0:4])[C+0:5].[O+0]=[O+0:6]>>[*:1]=[*:2].[*:6][*:3]([*:4])[*:5]",<br>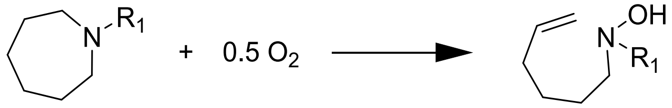<br>"[C+0!H0:1][C+0:2]@[N+0:3]([C+0:4])[C+0:5].[O+0]=[O+0:6]>>([*:1]=[*:2].[*:6][*:3]([*:4])[*:5])", |
| Hemiaminal Formation | 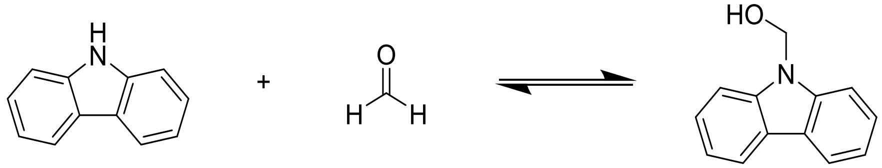<br>"[CX3!$(*O)+0:1]=[O+0:2].[N,n;+0X3!H0:3]>>[*:1]([*:2])[*:3]",<br>"[CX4!$(*(O)O)+0:1]([O+0H:2])-!@[N,n;+0X3:3]>>[*:1]=[*:2].[*:3]", |
| Hemiaminal Dehydration | 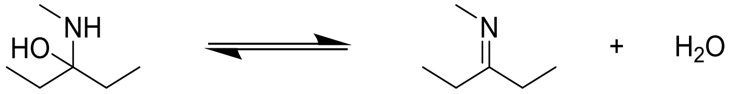<br>"[C+0:1]([O+0H:2])[N+0X3!H0:3]>>[*:1]=[*:3].[*:2]",<br>"[C+0:1]=[N+0:3].[O+0H2:2]>>[*:1]([*:2])[*:3]", |
| | **Nitroso, Nitro Compounds** |
| Reduction of Nitroso | 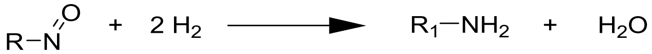<br>"[C,c;+0:1][N+0:2]=[O+0:3].[H][H]>>[*:1][*:2].[*:3]", |

**Reduction of Nitro Compounds**

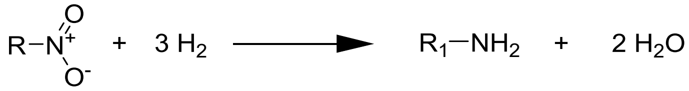

"[C,c;+0:1][N+1](=[O+0:3])[O-1H0].[H][H]>>[*:1][N].[*:3]",

**Henry Reaction**

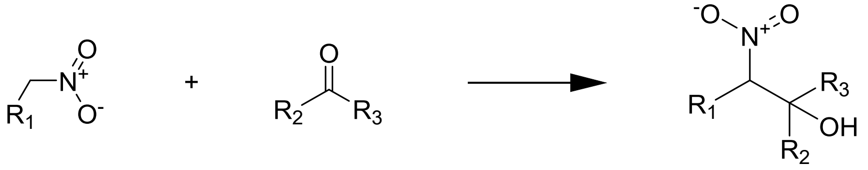

"[C+0!H0:1][N+1:2](=[O+0:3])[OX1-
1:4].[CX3!$(*([OH])[OH])+0:5]=[O+0:6]>>[*:6][*:5][*:1][*:2](=[*:3])[*:4]",

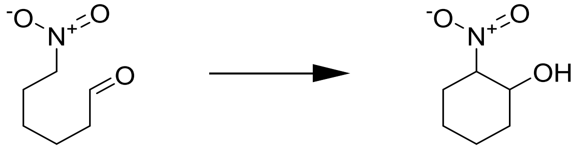

"([C+0!H0:1][N+1:2](=[O+0:3])[OX1-
1:4].[CX3!$(*([OH])[OH])+0:5]=[O+0:6])>>[*:6][*:5][*:1][*:2](=[*:3])[*:4]",

**Benzene Nitration**

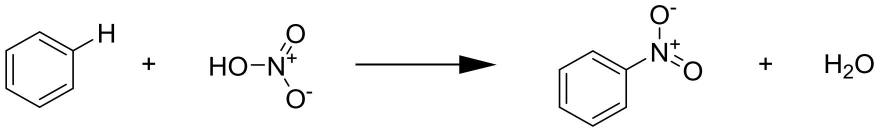

"[c+0H:1].[O+0H:2][N+1:3](=[O+0:4])[OX1-1:5]>>[*:1][*:3](=[*:4])[*:5].[*:2]",

**Oxidation of Nitroso**

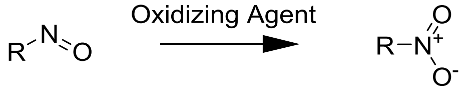

"[*+0:1][N+0]=[O+0].[O+0]=[O+0]>>[*:1][N+1](=[O])[O-1]",

**Esterification, Acid Anhydride Formation with Nitric Acid, Nitrate Esters Hydrolysis**

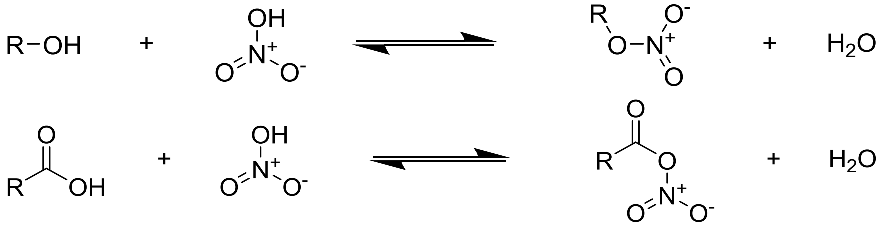

"[C,c;+0:1][O+0H:2].[O+0H:3][N+1:4](=[O+0:5])[OX1-1:6]>>[*:1][*:2][*:4](=[*:5])[*:6].[*:3]",
"[C,c;+0:1][O+0:2][N+1:4](=[O+0:5])[OX1-1:6].[O+0H2:3]>>[*:1][*:2].[*:3][*:4](=[*:5])[*:6]",

**Nitrogen Dioxide Disproportionation**

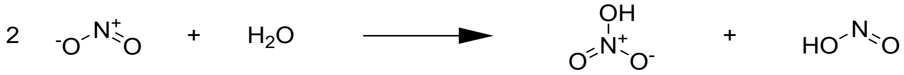

"[O-1][NX2+1]=[O].[O+0H2]>>[O-1][N+1](=[O])[O].[O][N]=[O]",

**Benzene Nitration, 2-step**

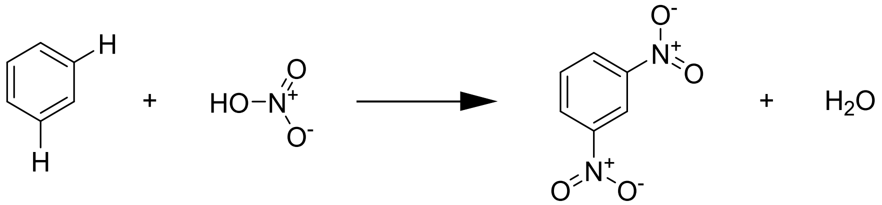

"[c!$(*aN)+0H:1][a+0:6][c!$(*aN)+0H:7].[O+0H:2][N+1:3](=[O+0:4])[OX1-
1:5]>>[*:1]([*:3](=[*:4])[*:5]):[*:6]:[*:7]([*:3](=[*:4])[*:5]).[*:2]",

| | |
|---|---|
| Reduction of Nitro Compounds, 2-step | 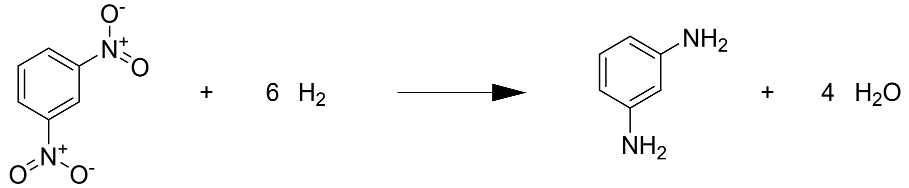<br>"([C,c;+0:1][N+1](=[O+0:3])[O-1H0].[C,c;+0:2][N+1](=[O+0])[O-1H0]).[H][H]>>([*:1][N].[*:2][N]).[*:3]", |
| Hydrogenation of Nitric Oxide | $NO + 1.5\ H_2 \xrightarrow{\text{Catalyst}} H_2N{-}OH$<br>"[NX1+0]=[O+0].[H][H]>>[N][O]", |

# Imines

| | |
|---|---|
| Imines from Aldehydes and Ketones | 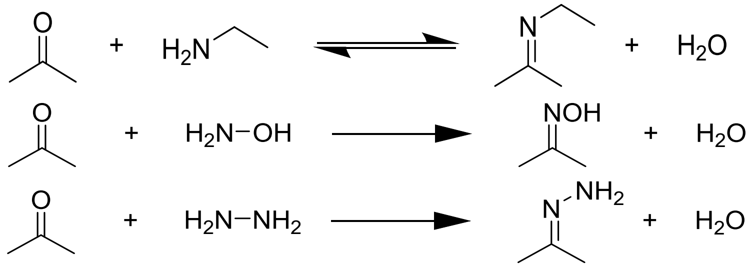<br>"[CX3!$(*[OH])+0:1]=[O+0:2].[N+0X3;H2,H3:3]>>[*:1]=[*:3].[*:2]",<br>"[CX3!$(*[OH])+0:1]=!@[N+0:3].[O+0H2:2]>>[*:1]=[*:2].[*:3]",<br>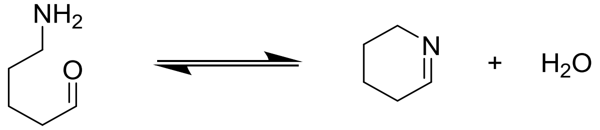<br>"([CX3!$(*[OH])+0:1]=[O+0:2].[N+0X3;H2,H3:3])>>[*:1]=[*:3].[*:2]",<br>"[CX3!$(*[OH])+0:1]=@[N+0:3].[O+0H2:2]>>([*:1]=[*:2].[*:3])", |
| Treatment of Aldehydes and Ketones with a Secondary Amine | 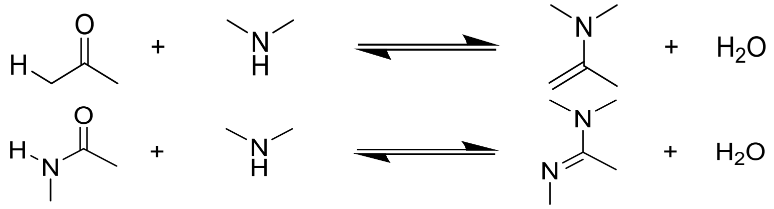<br>"[C,N;+0!H0:4][CX3!$(*[OH])+0:1]=[O+0:2].[N+0X3H:3]>>[*:4]=[*:1][*:3].[*:2]",<br>"[C,N;+0:4]=[CX3!$(*[OH])+0:1]-!@[NX3+0:3].[O+0H2:2]>>[*:4][*:1]=[*:2].[*:3]",<br>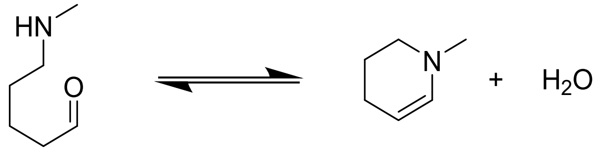<br>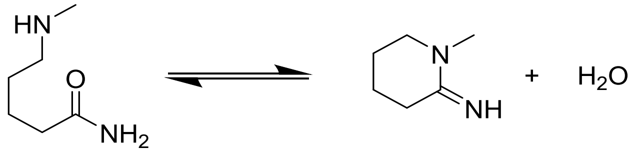<br>"([C,N;+0!H0:4][CX3!$(*[OH])+0:1]=[O+0:2].[N+0X3H:3])>>[*:4]=[*:1][*:3].[*:2]",<br>"[C,N;+0:4]=[CX3!$(*[OH])+0:1]-@[NX3+0:3].[O+0H2:2]>>([*:4][*:1]=[*:2].[*:3])", |

| | |
|---|---|
| Oxime-Nitroso Tautomerization | 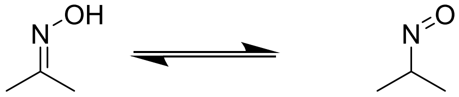<br>Oxime Nitroso<br>"[C+0:1]=[N+0:2][O+0H:3]>>[*:1][*:2]=[*:3]",<br>"[C+0!H0:1][N+0:2]=[O+0:3]>>[*:1]=[*:2][*:3]", |
| Reduction of Imines | 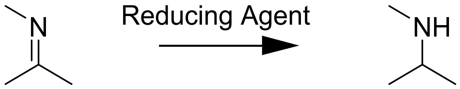<br><br>"[C+0:1]=[N+0:2].[H][H]>>[*:1][*:2]", |
| Beckmann Rearrangement | 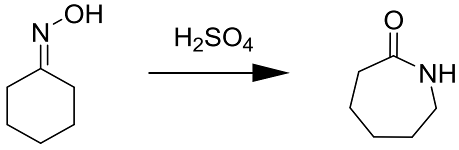<br>"[C,c;+0:1][C+0:2](=[N+0:3][O+0H:4])[C,c;+0:5]>>[*:1][*:2](=[*:4])[*:3][*:5]",<br>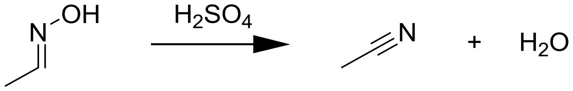<br>"[C,c;+0:1][C+0H:2](=[N+0:3][O+0H:4])>>[*:1][*:2]#[*:3].[*:4]", |
| Beckmann Rearrangement from Ketones and Aldehydes | 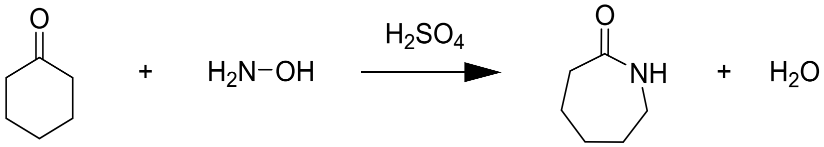<br>"[C,c;+0:1][C+0:2](=[O+0:3])[C,c;+0:4].[N+0H2:5][O+0H:6]>>[*:1][*:2](=[*:3])[*:5][*:4].[*:6]",<br>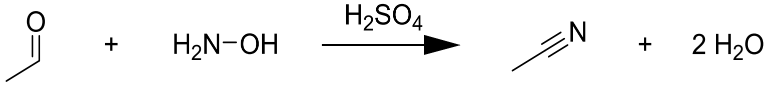<br>"[C,c;+0:1][C+0H:2]=[O+0:3].[N+0H2:4][O+0H]>>[*:1][*:2]#[*:4].[*:3]", |
| Beckmann Fragmentation | 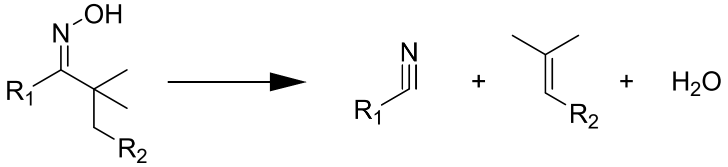<br>"[C,c;+0:1][C+0:2](=[N+0:3][O+0H:4])-!@[C+0:5]([C+0:6])([C+0:7])[*+0!H0:8]>>[*:1][*:2]#[*:3].[*:5]([*:6])([*:7])=[*:8].[*:4]",<br>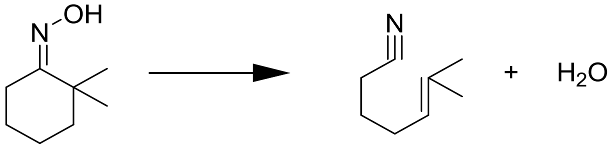<br>"[C,c;+0:1][C+0:2](=[N+0:3][O+0H:4])-@[C+0:5]([C+0:6])([C+0:7])[*+0!H0:8]>>([*:1][*:2]#[*:3].[*:5]([*:6])([*:7])=[*:8]).[*:4]", |
| Semmler–Wolff Reaction | 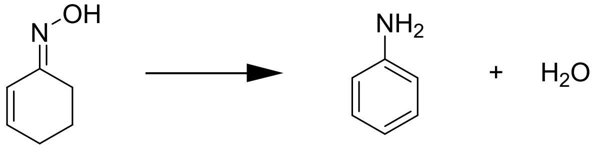<br>"[C+0!H0:1]1[C+0!H0:2][C+0!H0:3][C+0:4]=[C+0:5][C+0:6]1=[N+0:7][O+0H:8]>>[*:1]1[*:2]=[*:3][*:4]=[*:5][*:6]=1[*:7].[*:8]", |

| | |
|---|---|
| Transimination (Amine-Imine Exchange) | 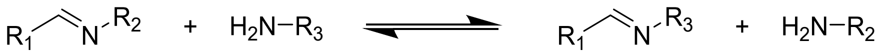  "[C,c;+0:1]=!@[N!$(*[O,S])+0:2].[N!$(*[O,S]);H2,H3;+0:3]>>[*:1]=[*:3].[*:2]", |
| Imine Metathesis (Imine-Imine Exchange) | 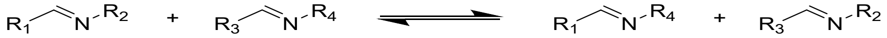  "[C,c;+0:1]=!@[N!$(*[O,S])+0:2].[C,c;+0:3]=!@[N!$(*[O,S])+0:4]>>[*:1]=[*:4].[*:3]=[*:2]", |
| Wolff–Kishner Reduction | 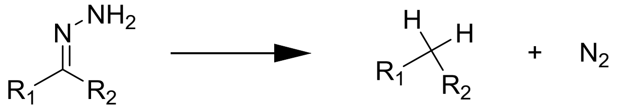  "[C,c;+0:1][C!$(*[O,S])+0:2](=[N+0:3][N+0H2:4])>>[*:1][*:2].[*:3]#[*:4]", |
| **Nitriles, Amino Acids** | |
| Cyanation (Kolbe Nitrile Synthesis) | 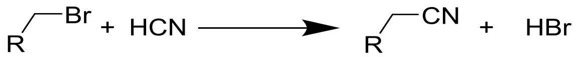  "[C,c+0:1][F,Cl,Br,I;+0:2].[C+0H:3]#[N+0:4]>>[*:1][*:3]#[*:4].[*:2]", |
| Cyanation of Ketones or Aldehydes (Cyanohydrin Reaction) | 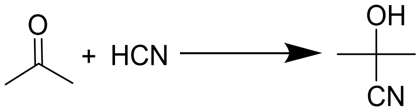  "[CX3!$(*[O,S,N])+0:1]=[O+0:2].[C+0H:3]#[N+0:4]>>[*:1]([*:3]#[*:4])[*:2]", |
| Alkyl Cyanides from Haloalkanes | 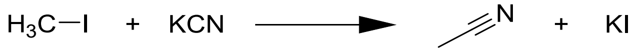  "[C+0:1][F,Cl,Br,I;+0:2].[C+0H:3]#[N+0:4]>>[*:1][*:3]#[*:4].[*:2]", |
| Nitrile from Primary Amides (Same as Dehydration of Amides) | 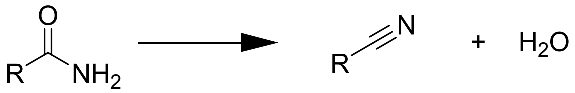  |
| Nitrile Hydrogenation to Amines | 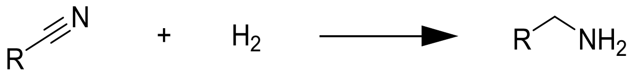  "[C+0:1]#[N+0:2].[H][H]>>[*:1][*:2]", |
| Amines Dehydrogenation to Nitriles | 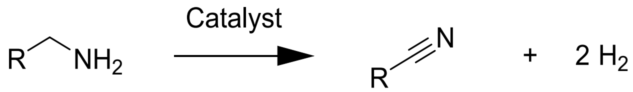  "[C+0;H2,H3:1]-[N+0H2:2]>>[*:1]#[*:2].[H][H]", |
| Amines Oxidative Dehydrogenation to Nitriles | 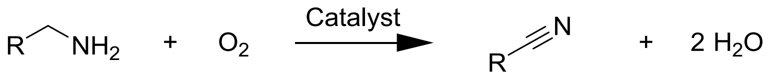  "[C+0;H2,H3:1]-[N+0H2:2].[O+0:3]=[O+0]>>[*:1]#[*:2].[*:3]", |

| | |
|---|---|
| Nitrile Hydrogenation to Aldehydes | 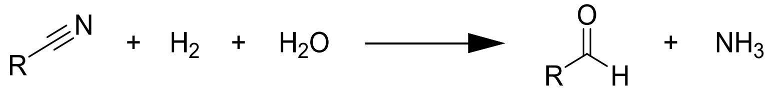 "[C+0:1]#[N+0:2].[O+0H2:3].[H][H]>>[*:1]=[*:3].[*:2]", |
| Nitrile Hydrogenation to Imines | 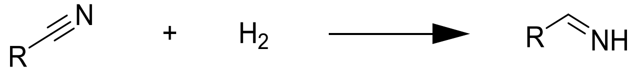 "[C+0:1]#[N+0:2].[H][H]>>[*:1]=[*:2]", |
| Nitrile Hydrogenation to Secondary Amines | 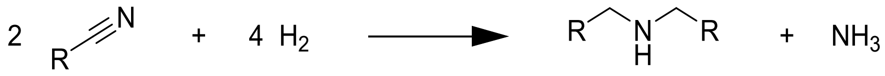 "[C+0:1]#[N+0:2].[C+0:3]#[N+0:4].[H][H]>>[*:1][*:2][*:3].[*:4]", 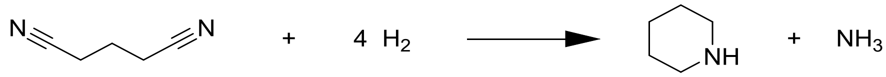 "([C+0:1]#[N+0:2].[C+0:3]#[N+0:4]).[H][H]>>[*:1][*:2][*:3].[*:4]", |
| Nitrile Hydrogenation to Tertiary Amines | 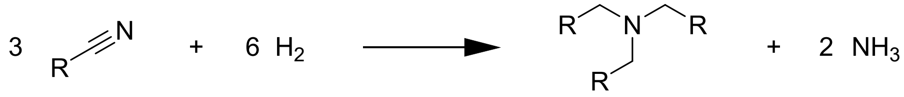 "[C+0:1]#[N+0:2].[C+0:3]#[N+0:4].[C+0:5]#[N+0].[H][H]>>[*:1][*:2]([*:3])[*:5].[*:4]", |
| Hydrolysis of Nitriles | 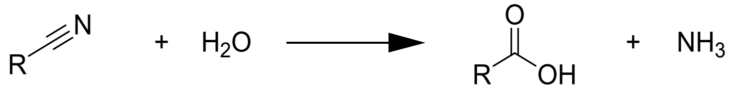 "[C+0:1]#[N+0:2].[O+0H2:3]>>[*:1](=[O])[*:3].[*:2]", |
| Nitriles to Ketones with Grignard Reagents (Combined with Grignard Reagent Formation) | 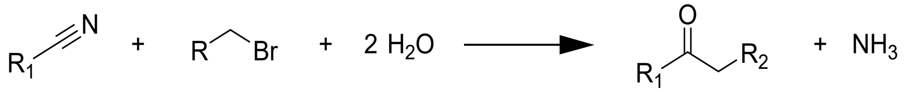 "[C+0:1]#[N+0:2].[c,C!$(*~O);!$(*#*);+0:3][F,Cl,Br,I;+0].[O+0H2:4]>>[*:1](=[*:4])[*:3].[*:2]", |
| Partial Hydrolysis of Nitriles | 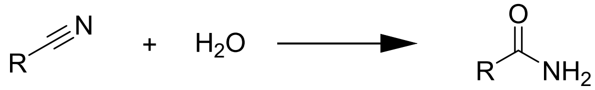 "[C+0:1]#[N+0:2].[O+0H2:3]>>[*:1](=[*:3])[*:2]", |
| Epoxides Ring Opening with Cyanides | 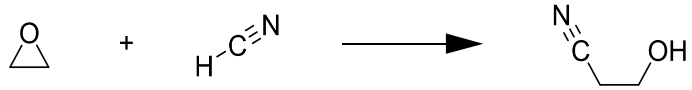 "[C+0:1]1[C+0:2][O+0:3]1.[C+0H:4]#[N+0:5]>>[*:5]#[*:4][*:1][*:2][*:3]", |
| Andrussow Process | $2\ CH_4 + 2\ NH_3 + 3\ O_2 \xrightarrow{\text{Catalyst}} 2\ HCN + 6\ H_2O$ "[C+0H4:1].[N+0H3:2].[O+0:3]=[O+0]>>[*:1]#[*:2].[*:3]", |
| Hydrocyanation | 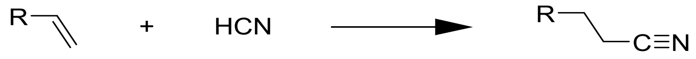 "[C+0:1]=[C+0:2].[C+0H:3]#[N+0:4]>>[*:1][*:2][*:3]#[*:4]", |

| | |
|---|---|
| Hydrocyanation, 2-step | 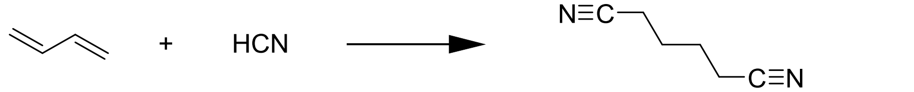 "([C+0:1]=[C+0:2].[C+0:3]=[C+0:4]).[C+0H:5]#[N+0:6]>>([*:1][*:2][*:5]#[*:6].[*:3][*:4][*:5]#[*:6])", |
| Ammoxidation | 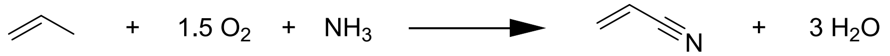 "[C+0:1]=[C+0:2][C+0H3:3].[O+0:4]=[O+0].[N+0H3:5]>>[*:1]=[*:2][*:3]#[*:5].[*:4]", |
| Bucherer–Bergs Reaction | 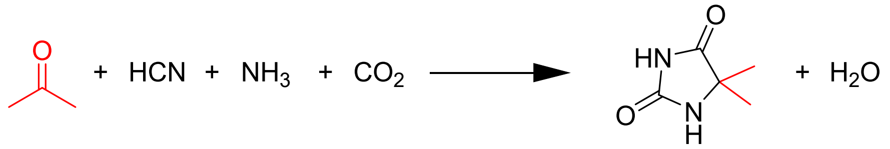 "[CX3!$(*-[O])+0:1]=[O+0:2].[C+0H:3]#[N+0:4].[N+0H3:5].[O+0:6]=[C+0:7]=[O+0:8]>>[*:1]1[*:3](=[*:2])[*:4][*:7](=[*:6])[*:5]1.[*:8]", |
| **Azides** | |
| Azide-Alkyne, Azide-Nitrile Cycloaddition | 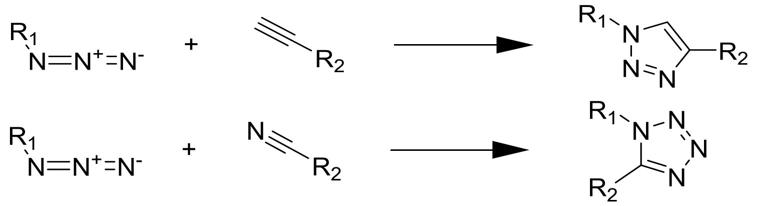 "[N+0:1]=[N+1]=[N-1].[C,N;+0:4]#[C+0:5]>>[*:1]1[N]=[N][*:4]=[*:5]1", |
| Azide Salts as Nucleophiles | 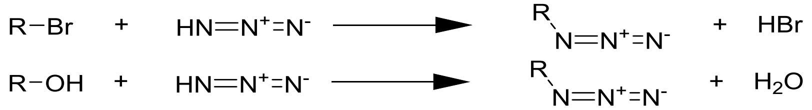 "[C+0:1][F,Cl,Br,I,OH;+0:2].[N+0H:3]=[N+1:4]=[N-1:5]>>[*:1][*:3]=[*:4]=[*:5].[*:2]", |
| Reduction of Azides | 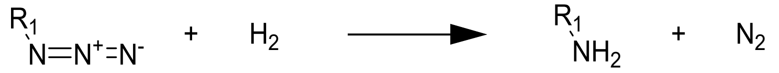 "[N+0:1]=[N+1]=[N-1].[H][H]>>[*:1].[N]#[N]", |
| Curtius Rearrangement | 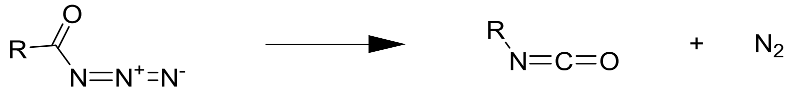 "[C,c;+0:1][C+0:2](=[O+0:3])[N+0:4]=[N+1]=[N-1]>>[*:1][*:4]=[*:2]=[*:3].[N]#[N]", |
| **Diazo** | |
| Esters from Diazoes and Carboxylic Acids | 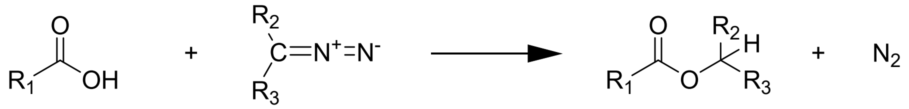 "[CX3!$(*([OH])[OH])+0:1](=[O+0:2])[O+0H:3].[CX3+0:4]=[N+1]=[N-1]>>[*:1](=[*:2])[*:3][*:4].[N]#[N]", |
| **Isocyanates, Isothiocyanate** | |

| | |
|---|---|
| Isocyanates, Isothiocyanate with Nucleophiles | 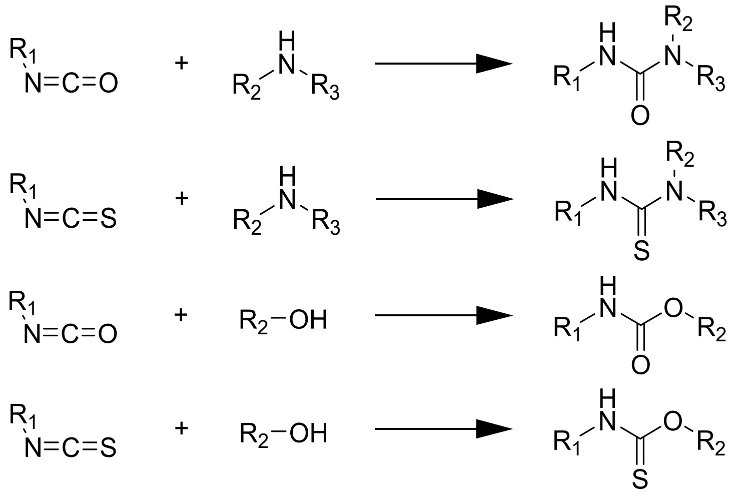<br>"[C,c;+0:1][N+0:2]=[C+0:3]=[O,S;+0:4].[NX3!H0,O!H0;+0:5]>>[*:1][*:2][*:3](=[*:4])[*:5]", |
| Isocyanates with Water | 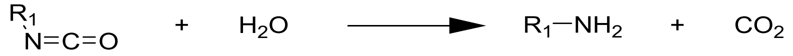<br>"[C,c;+0:1][N+0:2]=[C+0:3]=[O+0:4].[OH2+0:5]>>[*:1][*:2].[*:3](=[*:4])=[*:5]", |
| Isocyanates with Carboxylic Acid | 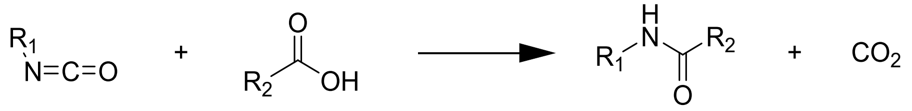<br>"[C,c;+0:1][N+0:2]=[C+0:3]=[O+0:4].[O+0H:5][C+0:6](=[O+0:7])[C,c;+0:8]>>[*:1][*:2][*:3](=[*:4])[*:8].[*:5]=[*:6]=[*:7]", |
| Synthesis of Isocyanates, Isothiocyanates | 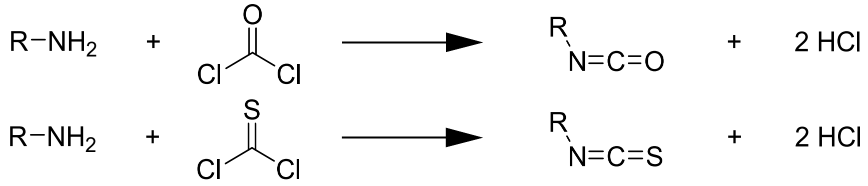<br>"[C,c;+0:1][N+0H2:2].[Cl+0][C+0:4](=[O,SX1;+0:5])[Cl+0:6]>>[*:1][*:2]=[*:4]=[*:5].[*:6]",<br>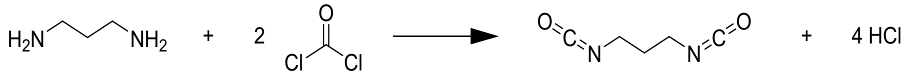<br>"([C,c;+0:1][N+0H2:2].[C,c;+0:7][N+0H2:8]).[Cl+0][C+0:4](=[O,SX1;+0:5])[Cl+0:6]>>([*:1][*:2]=[*:4]=[*:5].[*:7][*:8]=[C]=[O]).[*:6]", |
| Synthesis of Isothiocyanates | 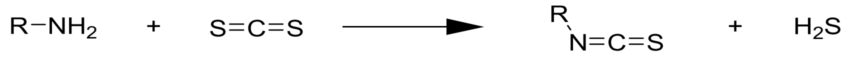<br>"[C,c;+0:1][N+0H2:2].[S+0:3]=[C+0:4]=[S+0:5]>>[*:1][*:2]=[*:4]=[*:5].[*:3]", |

# Thiols, Thioethers

| | |
|---|---|
| Hydrosulfide Anion Substitution with Alkyl Halides<br><br>(Ions) | 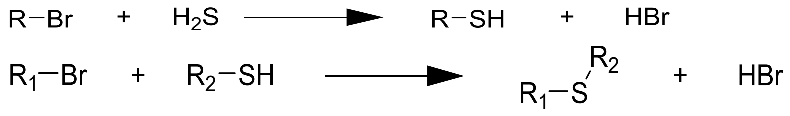<br>"[C,c;+0:1][F,Cl,Br,I;+0:2].[S!H0X2+0:3]>>[*:1][*:3].[*:2]",<br>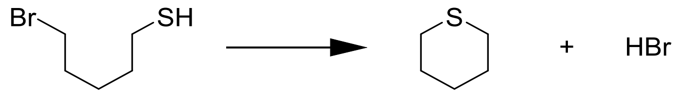<br>"([C,c;+0:1][F,Cl,Br,I;+0:2].[S!H0X2+0:3])>>[*:1][*:3].[*:2]", |

| | |
|---|---|
| Preparation of Sulfides<br><br>(Ions) | 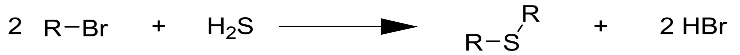<br>"[C,c;+0:1][F,Cl,Br,I;+0:2].[C,c;+0:3][F,Cl,Br,I;+0].[SH2X2+0:5]>>[*:1][*:5][*:3].[*:2]",<br>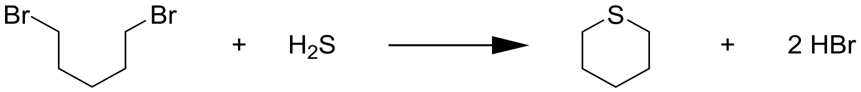<br>"([C,c;+0:1][F,Cl,Br,I;+0:2].[C,c;+0:3][F,Cl,Br,I;+0]).[SH2X2+0:5]>>[*:1][*:5][*:3].[*:2]", |
| Thiols from Thiourea<br><br>(Ions) | 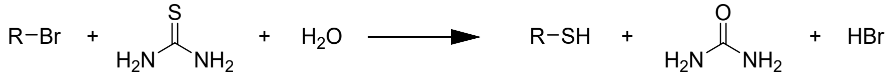<br>"[C,c;+0:1][F,Cl,Br,I;+0:2].[SX1+0:3]=[C+0:4]([N+0H2:5])[N+0H2:6].[O+0H2:7]>>[*:1][*:3].[*:7]=[*:4]([*:5])[*:6].[*:2]", |
| Thioacetals from Thiols and Aldehydes or Ketones | 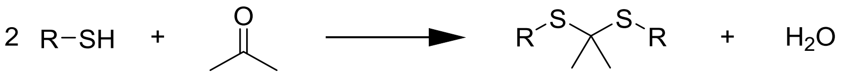<br>"[C,c;+0:1][SX2+0H:2].[C,c;+0:3][SX2+0H:4].[C!$(*O)X3+0:5]=[O+0:6]>>[*:1][*:2][*:5][*:4][*:3].[*:6]", |
| Cyclic Thioacetals from Dithiols | 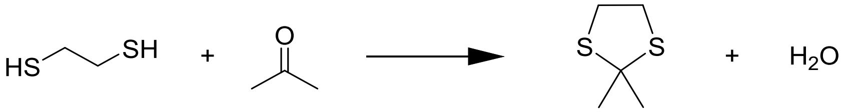<br>"([C,c;+0:1][SX2+0H:2].[C,c;+0:3][SX2+0H:4]).[C!$(*O)X3+0:5]=[O+0:6]>>[*:1][*:2][*:5][*:4][*:3].[*:6]", |
| Oxidation of Thiols, Reduction of Disulfides | 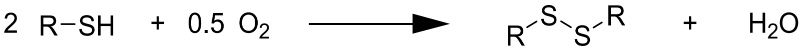<br>"[C,c;+0:1][SX2+0H:2].[C,c;+0:3][SX2+0H:4].[O+0:5]=[O+0]>>[*:1][*:2][*:4][*:3].[*:5]",<br>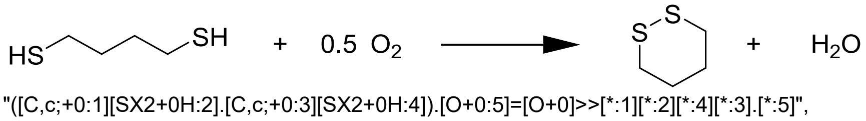<br>"([C,c;+0:1][SX2+0H:2].[C,c;+0:3][SX2+0H:4]).[O+0:5]=[O+0]>>[*:1][*:2][*:4][*:3].[*:5]",<br>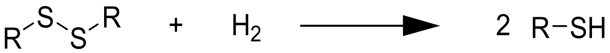<br>"[C,c;+0:1][SX2+0:2]-!@[SX2+0:4][C,c;+0:3].[H][H]>>[*:1][*:2].[*:3][*:4]",<br>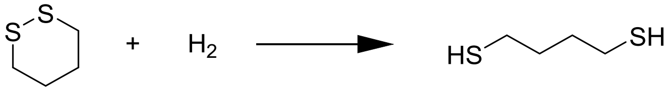<br>"[C,c;+0:1][SX2+0:2]-@[SX2+0:4][C,c;+0:3].[H][H]>>([*:1][*:2].[*:3][*:4])", |
| Thiol-ene Reaction | 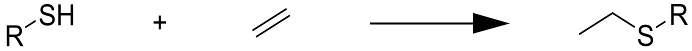<br>"[C,c;+0:1][SX2+0H:2].[C+0:3]=[C+0:4]>>[*:1][*:2][*:3][*:4]",<br>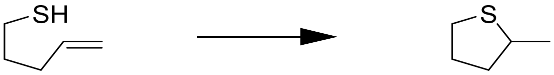<br>"([C,c;+0:1][SX2+0H:2].[C+0:3]=[C+0:4])>>[*:1][*:2][*:3][*:4]", |
| Sulfides Hydrogenolysis | 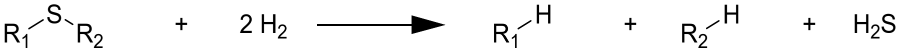<br>"[C,c;+0:1]-!@[SX2+0:2][C,c;+0:3].[H][H]>>[*:1].[*:3].[*:2]",<br>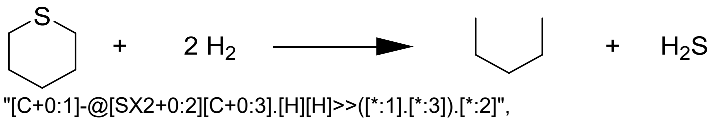<br>"[C+0:1]-@[SX2+0:2][C+0:3].[H][H]>>([*:1].[*:3]).[*:2]", |

| | |
|---|---|
| Oxidation of Disulfides | 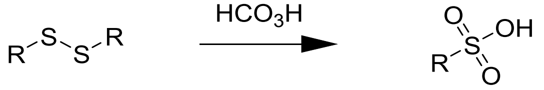<br>"[C,c;+0:1]-!@[SX2+0:2][SX2+0:3][C,c;+0:4].[O+0:5]=[O+0:6].[O+0H2:7]>>[*:1][*:2](=[*:5])(=[*:6])[*:7].[*:4][*:3](=[O])(=[O])[O]",<br>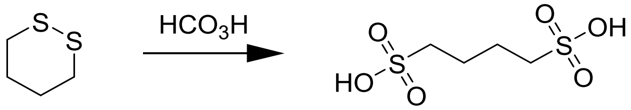<br>"[C,c;+0:1]-@[SX2+0:2][SX2+0:3][C,c;+0:4].[O+0:5]=[O+0:6].[O+0H2:7]>>([*:1][*:2](=[*:5])(=[*:6])[*:7].[*:4][*:3](=[O])(=[O])[O])", |
| Cleavage of Disulfides by Halogens | 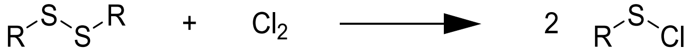<br>"[C,c;+0:1]-!@[SX2+0:2][SX2+0:3][C,c;+0:4].[F,Cl,Br,I;+0:5][F,Cl,Br,I;+0:6]>>[*:1][*:2][*:5].[*:4][*:3][*:6]",<br>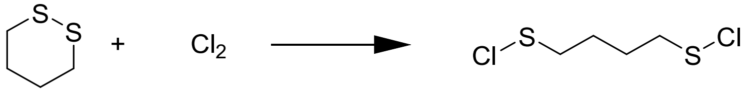<br>"[C,c;+0:1]-@[SX2+0:2][SX2+0:3][C,c;+0:4].[F,Cl,Br,I;+0:5][F,Cl,Br,I;+0:6]>>([*:1][*:2][*:5].[*:4][*:3][*:6])", |
| **Sulfoxides** | |
| Oxidation of Sulfides, Sulfoxides | 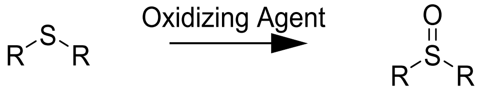<br>"[*+0:1][SX2!$(*[OH])+0:2][*+0:3].[O+0:4]=[O+0]>>[*:1][*:2](=[*:4])[*:3]",<br>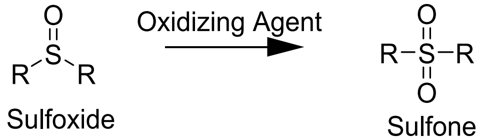<br>"[*+0:1][SX3+0:2](=[O+0:4])[*+0:3].[O+0:5]=[O+0]>>[*:1][*:2](=[*:4])(=[*:5])[*:3]", |
| Deoxygenation of Sulfoxides | 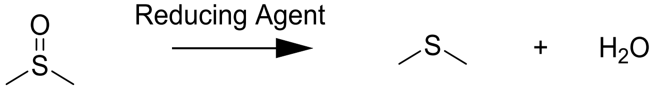<br>"[C,c;+0:1][SX3+0:2](=[O+0:4])[C,c;+0:3].[H][H]>>[*:1][*:2][*:3].[*:4]", |
| Thermal Elimination of Sulfoxides | 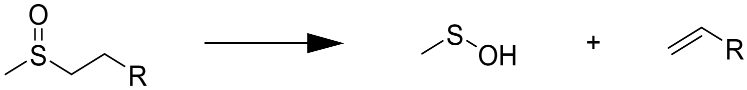<br>"[C,c;+0:1][SX3+0:2](=[O+0:3])-!@[C+0:4][C+0!H0:5]>>[*:1][*:2][*:3].[*:4]=[*:5]", |
| **Thiones** | |
| Thione-Thiol Tautomerization | 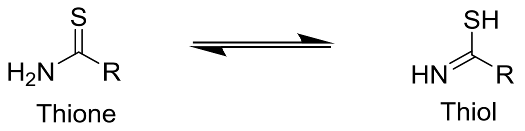<br>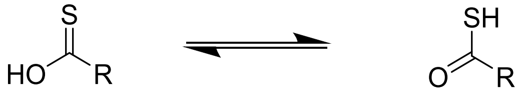<br>"[N,O;+0!H0:1][C+0:2]=[SX1+0:3]>>[*:1]=[*:2][*:3]",<br>"[N,O;+0:1]=[C+0:2][SX2+0H:3]>>[*:1][*:2]=[*:3]", |

# Sulfenic, Sulfinic, Sulfonic, Sulfuric Acids

## Sulfenic Acid Tautomerization

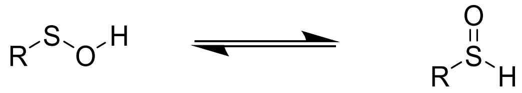

"[C,c;+0:1][SX2+0:2][O+0H:3]>>[*:1][*:2]=[*:3]",
"[C,c;+0:1][SX3+0H:2]=[O+0:3]>>[*:1][*:2][*:3]",

## Thiol Oxidation to Sulfenic Acid, Sulfinic Acid, Sulfonic Acid

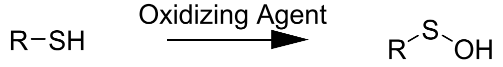


Sulfenic Acid

"[C,c;+0:1][SX2+0H:2].[O+0:3]=[O+0]>>[*:1][*:2][*:3]",

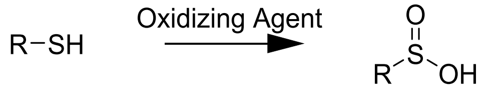


Sulfinic Acid

"[C,c;!$(*=O)+0:1][SX2+0H:2].[O+0:3]=[O+0:4]>>[*:1][*:2](=[*:3])[*:4]",

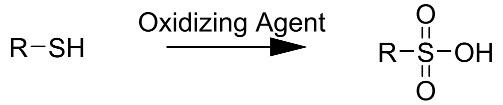


Sulfonic Acid

"[C,c;!$(*=O)+0:1][SX2+0H:2].[O+0:3]=[O+0:4]>>[*:1][*:2](=[*:3])(=[O])[*:4]",

## Sulfurous Acid Salts Addition to Oxiranes

(Ion)

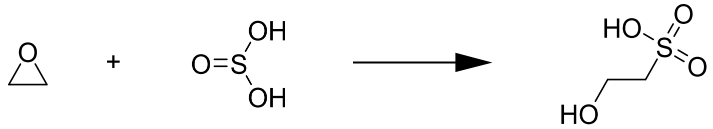

"[CX4+0:1]1[O+0:2][CX4+0:3]1.[O+0:4]=[SX3+0:5]([O+0H:6])[O+0H:7]>>[*:2][*:1][*:3][*:5](=[*:4])(=[*:6])[*:7]",

## Sulfuric Acid Esterification, Acid Anhydride Formation, Organosulfates Hydrolysis

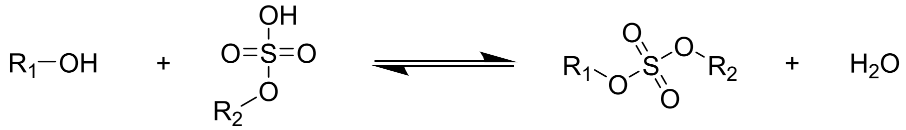

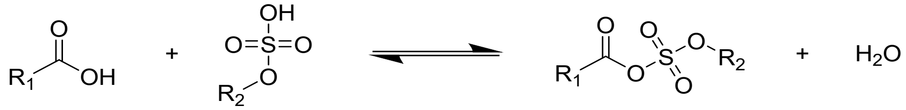

"[C,c;+0:1][O+0H:2].[O+0:3]=[SX4+0:4](=[O+0:5])([OX2+0:6])[O+0H:7]>>[*:1][*:2][*:4](=[*:3])(=[*:5])[*:6].[*:7]",
"[C,c;+0:1][O+0:2]-!@[SX4+0:4](=[O+0:3])(=[O+0:5])[OX2+0:6].[O+0H2:7]>>[*:1][*:2].[*:3]=[*:4](=[*:5])([*:6])[*:7]",

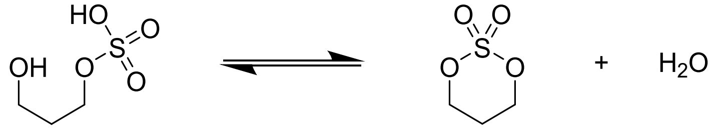

"([C,c;+0:1][O+0H:2].[O+0:3]=[SX4+0:4](=[O+0:5])([OX2+0:6])[O+0H:7])>>[*:1][*:2][*:4](=[*:3])(=[*:5])[*:6].[*:7]",
"[C,c;+0:1][O+0:2]-@[SX4+0:4](=[O+0:3])(=[O+0:5])[OX2+0:6].[O+0H2:7]>>([*:1][*:2].[*:3]=[*:4](=[*:5])([*:6])[*:7])",

| | |
|---|---|
| Organosulfates Reduction<br><br>(Ion) | 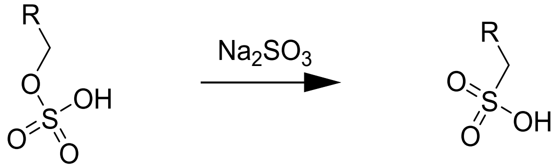<br><br>"[C!$(*=O)+0:1][O+0:2][SX4+0:3](=[O+0:4])(=[O+0:5])[O+0H:6].[H][H]>>[*:1][*:3](=[*:4])(=[*:5])[*:6].[*:2]", |
| Alkenes Addition by Sulfuric Acid | 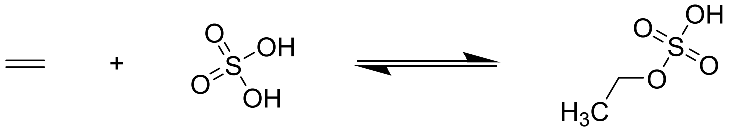<br><br>"[C+0:1]=[C+0:2].[O+0:3]=[SX4+0:4](=[O+0:5])([O+0H:6])[O+0H:7]>>[*:1][*:2][*:6][*:4](=[*:3])(=[*:5])[*:7]",<br>"[C+0!H0:1][C+0:2][O+0:6][SX4+0:4](=[O+0:3])(=[O+0:5])[O+0H:7]>>[*:1]=[*:2].[*:3]=[*:4](=[*:5])([*:6])[*:7]", |
| Alkenes Addition by Bisulfites<br><br>(Ion) | 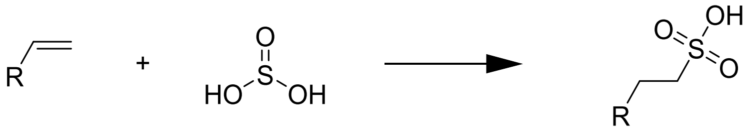<br><br>"[C+0:1]=[C+0:2].[O+0:3]=[SX3+0:4]([O+0H:5])[O+0H:6]>>[*:1][*:2][*:4](=[*:3])(=[*:5])[*:6]", |

| | |
|---|---|
| Esterification of Sulfonic Acids, Sulfonic Acid Esters Hydrolysis | 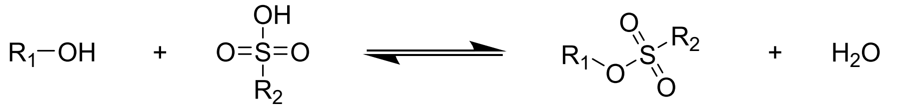<br>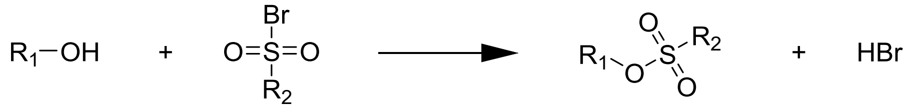<br>"[C,c;+0:1][O+0H:2].[O+0:3]=[SX4+0:4](=[O+0:5])([C,c,N;+0:6])[OH,F,Cl,Br,I;+0:7]>>[*:1][*:2][*:4](=[*:3])(=[*:5])[*:6].[*:7]",<br>"[C,c;+0:1][O+0:2]-!@[SX4+0:4](=[O+0:3])(=[O+0:5])[C,c,N;+0:6].[O+0H2:7]>>[*:1][*:2].[*:3]=[*:4](=[*:5])([*:6])[*:7]",<br>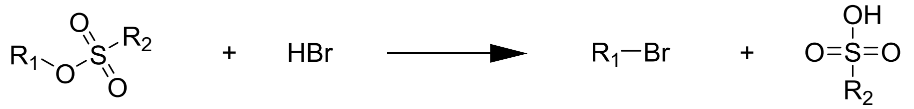<br>"[C,c;+0:1][O+0:2]-!@[SX4+0:4](=[O+0:3])(=[O+0:5])[C,c;+0:6].[F,Cl,Br,I;+0H:7]>>[*:1][*:7].[*:3]=[*:4](=[*:5])([*:6])[*:2]",<br>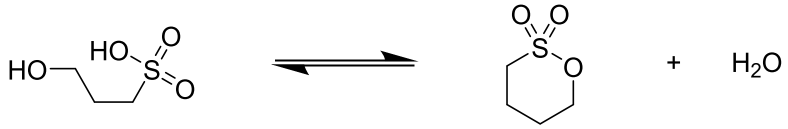<br>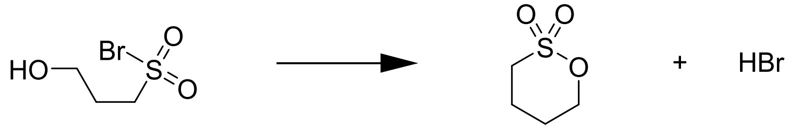<br>"([C,c;+0:1][O+0H:2].[O+0:3]=[SX4+0:4](=[O+0:5])([C,c,N;+0:6])[OH,F,Cl,Br,I;+0:7])>>[*:1][*:2][*:4](=[*:3])(=[*:5])[*:6].[*:7]",<br>"[C,c;+0:1][O+0:2]-@[SX4+0:4](=[O+0:3])(=[O+0:5])[C,c,N;+0:6].[O+0H2:7]>>([*:1][*:2].[*:3]=[*:4](=[*:5])([*:6])[*:7])",<br>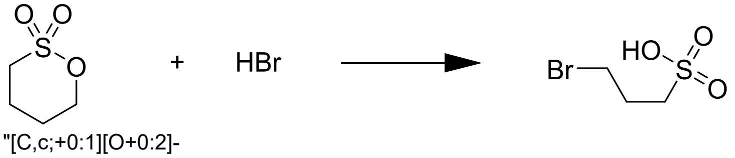<br>"[C,c;+0:1][O+0:2]-@[SX4+0:4](=[O+0:3])(=[O+0:5])[C,c;+0:6].[F,Cl,Br,I;+0H:7]>>([*:1][*:7].[*:3]=[*:4](=[*:5])([*:6])[*:2])", |
| Sulfonation of Benzene | 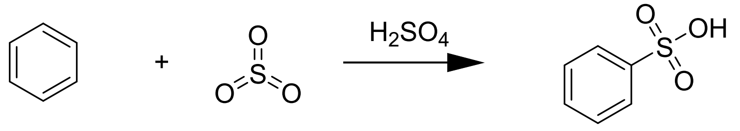<br>"[c+0H:1].[O+0:2]=[SX3+0:3](=[O+0:4])=[O+0:5]>>[*:1][*:3](=[*:4])(=[*:5])[*:2]", |
| Disproportionation of Aromatic Sulfinic Acids | 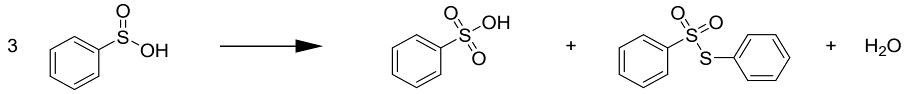<br>"[c+0:1][SX3+0:2](=[O+0])[O+0H]>>[*:1][*:2](=[O])(=[O])[O].[*:1][*:2](=[O])(=[O])[*:2][*:1].[O]", |
| Alkylation of Sulfinic Acids with Halides<br><br>(Ion) | 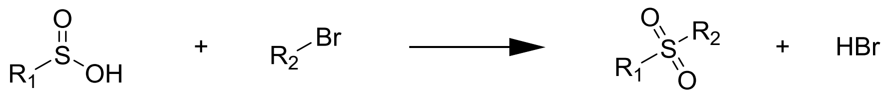<br>"[C,c;+0:1][SX3+0:2](=[O+0:3])[O+0H:4].[C,c;+0:5][F,Cl,Br,I;+0:6]>>[*:1][*:2](=[*:3])(=[*:4])[*:5].[*:6]", |

| | |
|---|---|
| Reduction of Aromatic Sulfonyl Chlorides to Thiols | 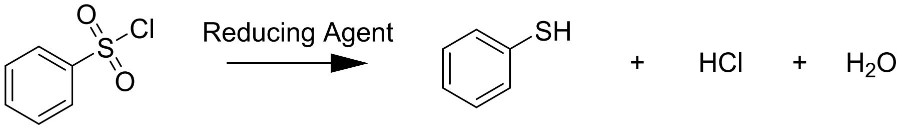<br><br>"[c+0:1][SX4+0:2](=[O+0:3])(=[O+0])[F,Cl,Br,I;+0:4].[H][H]>>[*:1][*:2].[*:4].[*:3]", |
| Reduction of Sulfonyl Chlorides to Sulfinic Acids<br><br>(Ion) | 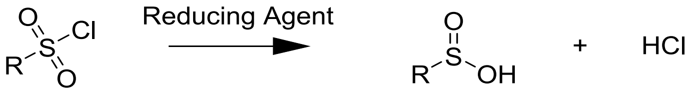<br><br>"[C,c;+0:1][SX4+0:2](=[O+0:3])(=[O+0:4])[F,Cl,Br,I;+0:5].[H][H]>>[*:1][*:2](=[*:3])[*:4].[*:5]", |
| Strecker Sulfite Alkylation<br><br>(Ion) | 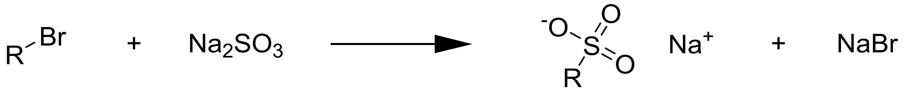<br><br>"[C,c;+0:1][F,Cl,Br,I;+0:2].[O+0:3]=[SX3+0:4]([O+0H:5])[O+0H:6]>>[*:1][*:4](=[*:3])(=[*:5])[*:6].[*:2]", |
| Hydrolysis of Sulfonyl Halides | 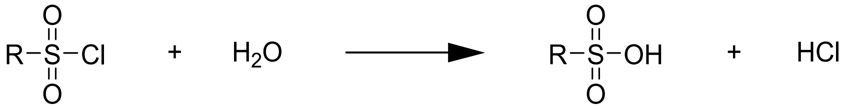<br><br>"[C,c;+0:1][SX4+0:2](=[O+0:3])(=[O+0:4])[F,Cl,Br,I;+0:5].[O+0H2:6]>>[*:1][*:2](=[*:3])(=[*:4])[*:6].[*:5]", |
| Reed Reaction | 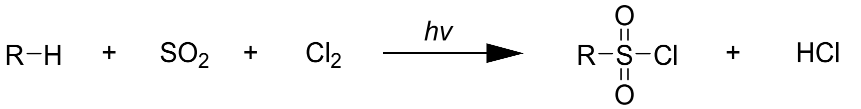<br><br>"[C,c;+0!H0:1].[SX2+0:2](=[O+0:3])(=[O+0:4]).[F,Cl,Br,I;+0:5][F,Cl,Br,I;+0:6]>>[*:1][*:2](=[*:3])(=[*:4])[*:6].[*:5]", |
| Sulfoxidation | 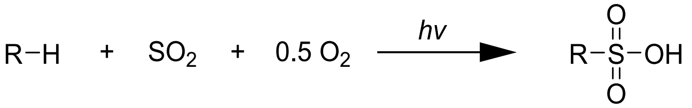<br><br>"[C+0!H0:1].[SX2+0:2](=[O+0:3])(=[O+0:4]).[O+0:5]=[O+0]>>[*:1][*:2](=[*:3])(=[*:4])[*:5]", |

## References


1. B. Elvers, *Ullmann's encyclopedia of industrial chemistry*, Verlag Chemie Hoboken, NJ, 1991.
2. Ethanol and unleaded gasoline average rack prices, https://dee.nebraska.gov/state-energy-information/energy-statistics/fuels/ethanol/ethanol-and-unleaded-gasoline-average-rack-prices, (accessed January 2015).
3. Z. Huang, R. G. Grim, J. A. Schaidle and L. Tao, *Energy & Environmental Science*, 2021, **14**, 3664-3678.

4. BusinessAnalytiq, Methanol price index, https://businessanalytiq.com/procurementanalytics/index/methanol-price-index/, (accessed January, 2025).
5. BusinessAnalytiq, Acetic Acid price index, https://businessanalytiq.com/procurementanalytics/index/acetic-acid-price-index/, (accessed January, 2025).
6. BusinessAnalytiq, Ethylene price index, https://businessanalytiq.com/procurementanalytics/index/ethylene-price-index/, (accessed January, 2025).
7. BusinessAnalytiq, Formaldehyde price index, https://businessanalytiq.com/procurementanalytics/index/formaldehyde-price-index/, (accessed January, 2025).
8. *Germany Pat.,* DE722707C, 1942.
9. S. Arsentev, *Russian Journal of Physical Chemistry A*, 2020, **94**, 1811-1815.
10. M. S. Alam, M. Camredon, A. R. Rickard, T. Carr, K. P. Wyche, K. E. Hornsby, P. S. Monks and W. J. Bloss, *Physical Chemistry Chemical Physics*, 2011, **13**, 11002-11015.
11. K. Maziarz, A. Tripp, G. Liu, M. Stanley, S. Xie, P. Gaiński, P. Seidl and M. H. Segler, *Faraday Discussions*, 2025, **256**, 568-586.
12. B. Delépine, T. Duigou, P. Carbonell and J.-L. Faulon, *Metabolic engineering*, 2018, **45**, 158-170.
13. T. Duigou, M. Du Lac, P. Carbonell and J.-L. Faulon, *Nucleic acids research*, 2019, **47**, D1229-D1235.
14. N. Schneider, N. Stiefl and G. A. Landrum, *Journal of chemical information and modeling*, 2016, **56**, 2336-2346.
15. P. Bansal, A. Morgat, K. B. Axelsen, V. Muthukrishnan, E. Coudert, L. Aimo, N. Hyka-Nouspikel, E. Gasteiger, A. Kerhornou and T. B. Neto, *Nucleic acids research*, 2022, **50**, D693-D700.
16. G. Bagnato, A. Iulianelli, A. Sanna and A. Basile, *Membranes*, 2017, **7**, 17.
17. T. C. Korosh, A. L. Markley, R. L. Clark, L. L. McGinley, K. D. McMahon and B. F. Pfleger, *Metabolic engineering*, 2017, **44**, 273-283.
